# Measurement of distributions sensitive to the underlying event in inclusive Z-boson production in $pp$ collisions at $\sqrt{s}=7$ TeV with the ATLAS detector

**DOI:** 10.1140/epjc/s10052-014-3195-6

**Published:** 2014-12-10

**Authors:** ATLAS Collaboration, G. Aad, B. Abbott, J. Abdallah, S. Abdel Khalek, O. Abdinov, R. Aben, B. Abi, M. Abolins, O. S. AbouZeid, H. Abramowicz, H. Abreu, R. Abreu, Y. Abulaiti, B. S. Acharya, L. Adamczyk, D. L. Adams, J. Adelman, S. Adomeit, T. Adye, T. Agatonovic-Jovin, J. A. Aguilar-Saavedra, M. Agustoni, S. P. Ahlen, F. Ahmadov, G. Aielli, H. Akerstedt, T. P. A. Åkesson, G. Akimoto, A. V. Akimov, G. L. Alberghi, J. Albert, S. Albrand, M. J. Alconada Verzini, M. Aleksa, I. N. Aleksandrov, C. Alexa, G. Alexander, G. Alexandre, T. Alexopoulos, M. Alhroob, G. Alimonti, L. Alio, J. Alison, B. M. M. Allbrooke, L. J. Allison, P. P. Allport, J. Almond, A. Aloisio, A. Alonso, F. Alonso, C. Alpigiani, A. Altheimer, B. Alvarez Gonzalez, M. G. Alviggi, K. Amako, Y. Amaral Coutinho, C. Amelung, D. Amidei, S. P. Amor Dos Santos, A. Amorim, S. Amoroso, N. Amram, G. Amundsen, C. Anastopoulos, L. S. Ancu, N. Andari, T. Andeen, C. F. Anders, G. Anders, K. J. Anderson, A. Andreazza, V. Andrei, X. S. Anduaga, S. Angelidakis, I. Angelozzi, P. Anger, A. Angerami, F. Anghinolfi, A. V. Anisenkov, N. Anjos, A. Annovi, A. Antonaki, M. Antonelli, A. Antonov, J. Antos, F. Anulli, M. Aoki, L. Aperio Bella, R. Apolle, G. Arabidze, I. Aracena, Y. Arai, J. P. Araque, A. T. H. Arce, J-F. Arguin, S. Argyropoulos, M. Arik, A. J. Armbruster, O. Arnaez, V. Arnal, H. Arnold, M. Arratia, O. Arslan, A. Artamonov, G. Artoni, S. Asai, N. Asbah, A. Ashkenazi, B. Åsman, L. Asquith, K. Assamagan, R. Astalos, M. Atkinson, N. B. Atlay, B. Auerbach, K. Augsten, M. Aurousseau, G. Avolio, G. Azuelos, Y. Azuma, M. A. Baak, A. Baas, C. Bacci, H. Bachacou, K. Bachas, M. Backes, M. Backhaus, J. Backus Mayes, E. Badescu, P. Bagiacchi, P. Bagnaia, Y. Bai, T. Bain, J. T. Baines, O. K. Baker, P. Balek, F. Balli, E. Banas, Sw. Banerjee, A. A. E. Bannoura, V. Bansal, H. S. Bansil, L. Barak, S. P. Baranov, E. L. Barberio, D. Barberis, M. Barbero, T. Barillari, M. Barisonzi, T. Barklow, N. Barlow, B. M. Barnett, R. M. Barnett, Z. Barnovska, A. Baroncelli, G. Barone, A. J. Barr, F. Barreiro, J. Barreiro Guimarães da Costa, R. Bartoldus, A. E. Barton, P. Bartos, V. Bartsch, A. Bassalat, A. Basye, R. L. Bates, J. R. Batley, M. Battaglia, M. Battistin, F. Bauer, H. S. Bawa, M. D. Beattie, T. Beau, P. H. Beauchemin, R. Beccherle, P. Bechtle, H. P. Beck, K. Becker, S. Becker, M. Beckingham, C. Becot, A. J. Beddall, A. Beddall, S. Bedikian, V. A. Bednyakov, C. P. Bee, L. J. Beemster, T. A. Beermann, M. Begel, K. Behr, C. Belanger-Champagne, P. J. Bell, W. H. Bell, G. Bella, L. Bellagamba, A. Bellerive, M. Bellomo, K. Belotskiy, O. Beltramello, O. Benary, D. Benchekroun, K. Bendtz, N. Benekos, Y. Benhammou, E. Benhar Noccioli, J. A. Benitez Garcia, D. P. Benjamin, J. R. Bensinger, K. Benslama, S. Bentvelsen, D. Berge, E. Bergeaas Kuutmann, N. Berger, F. Berghaus, J. Beringer, C. Bernard, P. Bernat, C. Bernius, F. U. Bernlochner, T. Berry, P. Berta, C. Bertella, G. Bertoli, F. Bertolucci, C. Bertsche, D. Bertsche, M. I. Besana, G. J. Besjes, O. Bessidskaia, M. Bessner, N. Besson, C. Betancourt, S. Bethke, W. Bhimji, R. M. Bianchi, L. Bianchini, M. Bianco, O. Biebel, S. P. Bieniek, K. Bierwagen, J. Biesiada, M. Biglietti, J. Bilbao De Mendizabal, H. Bilokon, M. Bindi, S. Binet, A. Bingul, C. Bini, C. W. Black, J. E. Black, K. M. Black, D. Blackburn, R. E. Blair, J.-B. Blanchard, T. Blazek, I. Bloch, C. Blocker, W. Blum, U. Blumenschein, G. J. Bobbink, V. S. Bobrovnikov, S. S. Bocchetta, A. Bocci, C. Bock, C. R. Boddy, M. Boehler, J. Boek, J. Boek, T. T. Boek, J. A. Bogaerts, A. G. Bogdanchikov, A. Bogouch, C. Bohm, J. Bohm, V. Boisvert, T. Bold, V. Boldea, A. S. Boldyrev, M. Bomben, M. Bona, M. Boonekamp, A. Borisov, G. Borissov, M. Borri, S. Borroni, J. Bortfeldt, V. Bortolotto, K. Bos, D. Boscherini, M. Bosman, H. Boterenbrood, J. Boudreau, J. Bouffard, E. V. Bouhova-Thacker, D. Boumediene, C. Bourdarios, N. Bousson, S. Boutouil, A. Boveia, J. Boyd, I. R. Boyko, J. Bracinik, A. Brandt, G. Brandt, O. Brandt, U. Bratzler, B. Brau, J. E. Brau, H. M. Braun, S. F. Brazzale, B. Brelier, K. Brendlinger, A. J. Brennan, R. Brenner, S. Bressler, K. Bristow, T. M. Bristow, D. Britton, F. M. Brochu, I. Brock, R. Brock, C. Bromberg, J. Bronner, G. Brooijmans, T. Brooks, W. K. Brooks, J. Brosamer, E. Brost, J. Brown, P. A. Bruckman de Renstrom, D. Bruncko, R. Bruneliere, S. Brunet, A. Bruni, G. Bruni, M. Bruschi, L. Bryngemark, T. Buanes, Q. Buat, F. Bucci, P. Buchholz, R. M. Buckingham, A. G. Buckley, S. I. Buda, I. A. Budagov, F. Buehrer, L. Bugge, M. K. Bugge, O. Bulekov, A. C. Bundock, H. Burckhart, S. Burdin, B. Burghgrave, S. Burke, I. Burmeister, E. Busato, D. Büscher, V. Büscher, P. Bussey, C. P. Buszello, B. Butler, J. M. Butler, A. I. Butt, C. M. Buttar, J. M. Butterworth, P. Butti, W. Buttinger, A. Buzatu, M. Byszewski, S. Cabrera Urbán, D. Caforio, O. Cakir, P. Calafiura, A. Calandri, G. Calderini, P. Calfayan, R. Calkins, L. P. Caloba, D. Calvet, S. Calvet, R. Camacho Toro, S. Camarda, D. Cameron, L. M. Caminada, R. Caminal Armadans, S. Campana, M. Campanelli, A. Campoverde, V. Canale, A. Canepa, M. Cano Bret, J. Cantero, R. Cantrill, T. Cao, M. D. M. Capeans Garrido, I. Caprini, M. Caprini, M. Capua, R. Caputo, R. Cardarelli, T. Carli, G. Carlino, L. Carminati, S. Caron, E. Carquin, G. D. Carrillo-Montoya, J. R. Carter, J. Carvalho, D. Casadei, M. P. Casado, M. Casolino, E. Castaneda-Miranda, A. Castelli, V. Castillo Gimenez, N. F. Castro, P. Catastini, A. Catinaccio, J. R. Catmore, A. Cattai, G. Cattani, V. Cavaliere, D. Cavalli, M. Cavalli-Sforza, V. Cavasinni, F. Ceradini, B. Cerio, K. Cerny, A. S. Cerqueira, A. Cerri, L. Cerrito, F. Cerutti, M. Cerv, A. Cervelli, S. A. Cetin, A. Chafaq, D. Chakraborty, I. Chalupkova, P. Chang, B. Chapleau, J. D. Chapman, D. Charfeddine, D. G. Charlton, C. C. Chau, C. A. Chavez Barajas, S. Cheatham, A. Chegwidden, S. Chekanov, S. V. Chekulaev, G. A. Chelkov, M. A. Chelstowska, C. Chen, H. Chen, K. Chen, L. Chen, S. Chen, X. Chen, Y. Chen, Y. Chen, H. C. Cheng, Y. Cheng, A. Cheplakov, R. Cherkaoui El Moursli, V. Chernyatin, E. Cheu, L. Chevalier, V. Chiarella, G. Chiefari, J. T. Childers, A. Chilingarov, G. Chiodini, A. S. Chisholm, R. T. Chislett, A. Chitan, M. V. Chizhov, S. Chouridou, B. K. B. Chow, D. Chromek-Burckhart, M. L. Chu, J. Chudoba, J. J. Chwastowski, L. Chytka, G. Ciapetti, A. K. Ciftci, R. Ciftci, D. Cinca, V. Cindro, A. Ciocio, P. Cirkovic, Z. H. Citron, M. Citterio, M. Ciubancan, A. Clark, P. J. Clark, R. N. Clarke, W. Cleland, J. C. Clemens, C. Clement, Y. Coadou, M. Cobal, A. Coccaro, J. Cochran, L. Coffey, J. G. Cogan, J. Coggeshall, B. Cole, S. Cole, A. P. Colijn, J. Collot, T. Colombo, G. Colon, G. Compostella, P. Conde Muiño, E. Coniavitis, M. C. Conidi, S. H. Connell, I. A. Connelly, S. M. Consonni, V. Consorti, S. Constantinescu, C. Conta, G. Conti, F. Conventi, M. Cooke, B. D. Cooper, A. M. Cooper-Sarkar, N. J. Cooper-Smith, K. Copic, T. Cornelissen, M. Corradi, F. Corriveau, A. Corso-Radu, A. Cortes-Gonzalez, G. Cortiana, G. Costa, M. J. Costa, D. Costanzo, D. Côté, G. Cottin, G. Cowan, B. E. Cox, K. Cranmer, G. Cree, S. Crépé-Renaudin, F. Crescioli, W. A. Cribbs, M. Crispin Ortuzar, M. Cristinziani, V. Croft, G. Crosetti, C.-M. Cuciuc, T. Cuhadar Donszelmann, J. Cummings, M. Curatolo, C. Cuthbert, H. Czirr, P. Czodrowski, Z. Czyczula, S. D’Auria, M. D’Onofrio, M. J. Da Cunha Sargedas De Sousa, C. Da Via, W. Dabrowski, A. Dafinca, T. Dai, O. Dale, F. Dallaire, C. Dallapiccola, M. Dam, A. C. Daniells, M. Dano Hoffmann, V. Dao, G. Darbo, S. Darmora, J. A. Dassoulas, A. Dattagupta, W. Davey, C. David, T. Davidek, E. Davies, M. Davies, O. Davignon, A. R. Davison, P. Davison, Y. Davygora, E. Dawe, I. Dawson, R. K. Daya-Ishmukhametova, K. De, R. de Asmundis, S. De Castro, S. De Cecco, N. De Groot, P. de Jong, H. De la Torre, F. De Lorenzi, L. De Nooij, D. De Pedis, A. De Salvo, U. De Sanctis, A. De Santo, J. B. De Vivie De Regie, W. J. Dearnaley, R. Debbe, C. Debenedetti, B. Dechenaux, D. V. Dedovich, I. Deigaard, J. Del Peso, T. Del Prete, F. Deliot, C. M. Delitzsch, M. Deliyergiyev, A. Dell’Acqua, L. Dell’Asta, M. Dell’Orso, M. Della Pietra, D. della Volpe, M. Delmastro, P. A. Delsart, C. Deluca, S. Demers, M. Demichev, A. Demilly, S. P. Denisov, D. Derendarz, J. E. Derkaoui, F. Derue, P. Dervan, K. Desch, C. Deterre, P. O. Deviveiros, A. Dewhurst, S. Dhaliwal, A. Di Ciaccio, L. Di Ciaccio, A. Di Domenico, C. Di Donato, A. Di Girolamo, B. Di Girolamo, A. Di Mattia, B. Di Micco, R. Di Nardo, A. Di Simone, R. Di Sipio, D. Di Valentino, F. A. Dias, M. A. Diaz, E. B. Diehl, J. Dietrich, T. A. Dietzsch, S. Diglio, A. Dimitrievska, J. Dingfelder, C. Dionisi, P. Dita, S. Dita, F. Dittus, F. Djama, T. Djobava, M. A. B. do Vale, A. Do Valle Wemans, D. Dobos, C. Doglioni, T. Doherty, T. Dohmae, J. Dolejsi, Z. Dolezal, B. A. Dolgoshein, M. Donadelli, S. Donati, P. Dondero, J. Donini, J. Dopke, A. Doria, M. T. Dova, A. T. Doyle, M. Dris, J. Dubbert, S. Dube, E. Dubreuil, E. Duchovni, G. Duckeck, O. A. Ducu, D. Duda, A. Dudarev, F. Dudziak, L. Duflot, L. Duguid, M. Dührssen, M. Dunford, H. Duran Yildiz, M. Düren, A. Durglishvili, M. Dwuznik, M. Dyndal, J. Ebke, W. Edson, N. C. Edwards, W. Ehrenfeld, T. Eifert, G. Eigen, K. Einsweiler, T. Ekelof, M. El Kacimi, M. Ellert, S. Elles, F. Ellinghaus, N. Ellis, J. Elmsheuser, M. Elsing, D. Emeliyanov, Y. Enari, O. C. Endner, M. Endo, R. Engelmann, J. Erdmann, A. Ereditato, D. Eriksson, G. Ernis, J. Ernst, M. Ernst, J. Ernwein, D. Errede, S. Errede, E. Ertel, M. Escalier, H. Esch, C. Escobar, B. Esposito, A. I. Etienvre, E. Etzion, H. Evans, A. Ezhilov, L. Fabbri, G. Facini, R. M. Fakhrutdinov, S. Falciano, R. J. Falla, J. Faltova, Y. Fang, M. Fanti, A. Farbin, A. Farilla, T. Farooque, S. Farrell, S. M. Farrington, P. Farthouat, F. Fassi, P. Fassnacht, D. Fassouliotis, A. Favareto, L. Fayard, P. Federic, O. L. Fedin, W. Fedorko, M. Fehling-Kaschek, S. Feigl, L. Feligioni, C. Feng, E. J. Feng, H. Feng, A. B. Fenyuk, S. Fernandez Perez, S. Ferrag, J. Ferrando, A. Ferrari, P. Ferrari, R. Ferrari, D. E. Ferreira de Lima, A. Ferrer, D. Ferrere, C. Ferretti, A. Ferretto Parodi, M. Fiascaris, F. Fiedler, A. Filipčič, M. Filipuzzi, F. Filthaut, M. Fincke-Keeler, K. D. Finelli, M. C. N. Fiolhais, L. Fiorini, A. Firan, A. Fischer, J. Fischer, W. C. Fisher, E. A. Fitzgerald, M. Flechl, I. Fleck, P. Fleischmann, S. Fleischmann, G. T. Fletcher, G. Fletcher, T. Flick, A. Floderus, L. R. Flores Castillo, A. C. Florez Bustos, M. J. Flowerdew, A. Formica, A. Forti, D. Fortin, D. Fournier, H. Fox, S. Fracchia, P. Francavilla, M. Franchini, S. Franchino, D. Francis, L. Franconi, M. Franklin, S. Franz, M. Fraternali, S. T. French, C. Friedrich, F. Friedrich, D. Froidevaux, J. A. Frost, C. Fukunaga, E. Fullana Torregrosa, B. G. Fulsom, J. Fuster, C. Gabaldon, O. Gabizon, A. Gabrielli, A. Gabrielli, S. Gadatsch, S. Gadomski, G. Gagliardi, P. Gagnon, C. Galea, B. Galhardo, E. J. Gallas, V. Gallo, B. J. Gallop, P. Gallus, G. Galster, K. K. Gan, J. Gao, Y. S. Gao, F. M. Garay Walls, F. Garberson, C. García, J. E. García Navarro, M. Garcia-Sciveres, R. W. Gardner, N. Garelli, V. Garonne, C. Gatti, G. Gaudio, B. Gaur, L. Gauthier, P. Gauzzi, I. L. Gavrilenko, C. Gay, G. Gaycken, E. N. Gazis, P. Ge, Z. Gecse, C. N. P. Gee, D. A. A. Geerts, Ch. Geich-Gimbel, K. Gellerstedt, C. Gemme, A. Gemmell, M. H. Genest, S. Gentile, M. George, S. George, D. Gerbaudo, A. Gershon, H. Ghazlane, N. Ghodbane, B. Giacobbe, S. Giagu, V. Giangiobbe, P. Giannetti, F. Gianotti, B. Gibbard, S. M. Gibson, M. Gilchriese, T. P. S. Gillam, D. Gillberg, G. Gilles, D. M. Gingrich, N. Giokaris, M. P. Giordani, R. Giordano, F. M. Giorgi, F. M. Giorgi, P. F. Giraud, D. Giugni, C. Giuliani, M. Giulini, B. K. Gjelsten, S. Gkaitatzis, I. Gkialas, L. K. Gladilin, C. Glasman, J. Glatzer, P. C. F. Glaysher, A. Glazov, G. L. Glonti, M. Goblirsch-Kolb, J. R. Goddard, J. Godlewski, C. Goeringer, S. Goldfarb, T. Golling, D. Golubkov, A. Gomes, L. S. Gomez Fajardo, R. Gonçalo, J. Goncalves Pinto Firmino Da Costa, L. Gonella, S. González de la Hoz, G. Gonzalez Parra, S. Gonzalez-Sevilla, L. Goossens, P. A. Gorbounov, H. A. Gordon, I. Gorelov, B. Gorini, E. Gorini, A. Gorišek, E. Gornicki, A. T. Goshaw, C. Gössling, M. I. Gostkin, M. Gouighri, D. Goujdami, M. P. Goulette, A. G. Goussiou, C. Goy, S. Gozpinar, H. M. X. Grabas, L. Graber, I. Grabowska-Bold, P. Grafström, K-J. Grahn, J. Gramling, E. Gramstad, S. Grancagnolo, V. Grassi, V. Gratchev, H. M. Gray, E. Graziani, O. G. Grebenyuk, Z. D. Greenwood, K. Gregersen, I. M. Gregor, P. Grenier, J. Griffiths, A. A. Grillo, K. Grimm, S. Grinstein, Ph. Gris, Y. V. Grishkevich, J.-F. Grivaz, J. P. Grohs, A. Grohsjean, E. Gross, J. Grosse-Knetter, G. C. Grossi, J. Groth-Jensen, Z. J. Grout, L. Guan, F. Guescini, D. Guest, O. Gueta, C. Guicheney, E. Guido, T. Guillemin, S. Guindon, U. Gul, C. Gumpert, J. Gunther, J. Guo, S. Gupta, P. Gutierrez, N. G. Gutierrez Ortiz, C. Gutschow, N. Guttman, C. Guyot, C. Gwenlan, C. B. Gwilliam, A. Haas, C. Haber, H. K. Hadavand, N. Haddad, P. Haefner, S. Hageböeck, Z. Hajduk, H. Hakobyan, M. Haleem, D. Hall, G. Halladjian, K. Hamacher, P. Hamal, K. Hamano, M. Hamer, A. Hamilton, S. Hamilton, G. N. Hamity, P. G. Hamnett, L. Han, K. Hanagaki, K. Hanawa, M. Hance, P. Hanke, R. Hann, J. B. Hansen, J. D. Hansen, P. H. Hansen, K. Hara, A. S. Hard, T. Harenberg, F. Hariri, S. Harkusha, D. Harper, R. D. Harrington, O. M. Harris, P. F. Harrison, F. Hartjes, M. Hasegawa, S. Hasegawa, Y. Hasegawa, A. Hasib, S. Hassani, S. Haug, M. Hauschild, R. Hauser, M. Havranek, C. M. Hawkes, R. J. Hawkings, A. D. Hawkins, T. Hayashi, D. Hayden, C. P. Hays, H. S. Hayward, S. J. Haywood, S. J. Head, T. Heck, V. Hedberg, L. Heelan, S. Heim, T. Heim, B. Heinemann, L. Heinrich, J. Hejbal, L. Helary, C. Heller, M. Heller, S. Hellman, D. Hellmich, C. Helsens, J. Henderson, Y. Heng, R. C. W. Henderson, C. Hengler, A. Henrichs, A. M. Henriques Correia, S. Henrot-Versille, C. Hensel, G. H. Herbert, Y. Hernández Jiménez, R. Herrberg-Schubert, G. Herten, R. Hertenberger, L. Hervas, G. G. Hesketh, N. P. Hessey, R. Hickling, E. Higón-Rodriguez, E. Hill, J. C. Hill, K. H. Hiller, S. Hillert, S. J. Hillier, I. Hinchliffe, E. Hines, M. Hirose, D. Hirschbuehl, J. Hobbs, N. Hod, M. C. Hodgkinson, P. Hodgson, A. Hoecker, M. R. Hoeferkamp, F. Hoenig, J. Hoffman, D. Hoffmann, J. I. Hofmann, M. Hohlfeld, T. R. Holmes, T. M. Hong, L. Hooft van Huysduynen, W. H. Hopkins, Y. Horii, J-Y. Hostachy, S. Hou, A. Hoummada, J. Howard, J. Howarth, M. Hrabovsky, I. Hristova, J. Hrivnac, T. Hryn’ova, C. Hsu, P. J. Hsu, S.-C. Hsu, D. Hu, X. Hu, Y. Huang, Z. Hubacek, F. Hubaut, F. Huegging, T. B. Huffman, E. W. Hughes, G. Hughes, M. Huhtinen, T. A. Hülsing, M. Hurwitz, N. Huseynov, J. Huston, J. Huth, G. Iacobucci, G. Iakovidis, I. Ibragimov, L. Iconomidou-Fayard, E. Ideal, P. Iengo, O. Igonkina, T. Iizawa, Y. Ikegami, K. Ikematsu, M. Ikeno, Y. Ilchenko, D. Iliadis, N. Ilic, Y. Inamaru, T. Ince, P. Ioannou, M. Iodice, K. Iordanidou, V. Ippolito, A. Irles Quiles, C. Isaksson, M. Ishino, M. Ishitsuka, R. Ishmukhametov, C. Issever, S. Istin, J. M. Iturbe Ponce, R. Iuppa, J. Ivarsson, W. Iwanski, H. Iwasaki, J. M. Izen, V. Izzo, B. Jackson, M. Jackson, P. Jackson, M. R. Jaekel, V. Jain, K. Jakobs, S. Jakobsen, T. Jakoubek, J. Jakubek, D. O. Jamin, D. K. Jana, E. Jansen, H. Jansen, J. Janssen, M. Janus, G. Jarlskog, N. Javadov, T. Javůrek, L. Jeanty, J. Jejelava, G.-Y. Jeng, D. Jennens, P. Jenni, J. Jentzsch, C. Jeske, S. Jézéquel, H. Ji, J. Jia, Y. Jiang, M. Jimenez Belenguer, S. Jin, A. Jinaru, O. Jinnouchi, M. D. Joergensen, K. E. Johansson, P. Johansson, K. A. Johns, K. Jon-And, G. Jones, R. W. L. Jones, T. J. Jones, J. Jongmanns, P. M. Jorge, K. D. Joshi, J. Jovicevic, X. Ju, C. A. Jung, R. M. Jungst, P. Jussel, A. Juste Rozas, M. Kaci, A. Kaczmarska, M. Kado, H. Kagan, M. Kagan, E. Kajomovitz, C. W. Kalderon, S. Kama, A. Kamenshchikov, N. Kanaya, M. Kaneda, S. Kaneti, V. A. Kantserov, J. Kanzaki, B. Kaplan, A. Kapliy, D. Kar, K. Karakostas, N. Karastathis, M. J. Kareem, M. Karnevskiy, S. N. Karpov, Z. M. Karpova, K. Karthik, V. Kartvelishvili, A. N. Karyukhin, L. Kashif, G. Kasieczka, R. D. Kass, A. Kastanas, Y. Kataoka, A. Katre, J. Katzy, V. Kaushik, K. Kawagoe, T. Kawamoto, G. Kawamura, S. Kazama, V. F. Kazanin, M. Y. Kazarinov, R. Keeler, R. Kehoe, M. Keil, J. S. Keller, J. J. Kempster, H. Keoshkerian, O. Kepka, B. P. Kerševan, S. Kersten, K. Kessoku, J. Keung, F. Khalil-zada, H. Khandanyan, A. Khanov, A. Khodinov, A. Khomich, T. J. Khoo, G. Khoriauli, A. Khoroshilov, V. Khovanskiy, E. Khramov, J. Khubua, H. Y. Kim, H. Kim, S. H. Kim, N. Kimura, O. Kind, B. T. King, M. King, R. S. B. King, S. B. King, J. Kirk, A. E. Kiryunin, T. Kishimoto, D. Kisielewska, F. Kiss, T. Kittelmann, K. Kiuchi, E. Kladiva, M. Klein, U. Klein, K. Kleinknecht, P. Klimek, A. Klimentov, R. Klingenberg, J. A. Klinger, T. Klioutchnikova, P. F. Klok, E.-E. Kluge, P. Kluit, S. Kluth, E. Kneringer, E. B. F. G. Knoops, A. Knue, D. Kobayashi, T. Kobayashi, M. Kobel, M. Kocian, P. Kodys, P. Koevesarki, T. Koffas, E. Koffeman, L. A. Kogan, S. Kohlmann, Z. Kohout, T. Kohriki, T. Koi, H. Kolanoski, I. Koletsou, J. Koll, A. A. Komar, Y. Komori, T. Kondo, N. Kondrashova, K. Köneke, A. C. König, S. König, T. Kono, R. Konoplich, N. Konstantinidis, R. Kopeliansky, S. Koperny, L. Köpke, A. K. Kopp, K. Korcyl, K. Kordas, A. Korn, A. A. Korol, I. Korolkov, E. V. Korolkova, V. A. Korotkov, O. Kortner, S. Kortner, V. V. Kostyukhin, V. M. Kotov, A. Kotwal, C. Kourkoumelis, V. Kouskoura, A. Koutsman, R. Kowalewski, T. Z. Kowalski, W. Kozanecki, A. S. Kozhin, V. Kral, V. A. Kramarenko, G. Kramberger, D. Krasnopevtsev, M. W. Krasny, A. Krasznahorkay, J. K. Kraus, A. Kravchenko, S. Kreiss, M. Kretz, J. Kretzschmar, K. Kreutzfeldt, P. Krieger, K. Kroeninger, H. Kroha, J. Kroll, J. Kroseberg, J. Krstic, U. Kruchonak, H. Krüger, T. Kruker, N. Krumnack, Z. V. Krumshteyn, A. Kruse, M. C. Kruse, M. Kruskal, T. Kubota, S. Kuday, S. Kuehn, A. Kugel, A. Kuhl, T. Kuhl, V. Kukhtin, Y. Kulchitsky, S. Kuleshov, M. Kuna, J. Kunkle, A. Kupco, H. Kurashige, Y. A. Kurochkin, R. Kurumida, V. Kus, E. S. Kuwertz, M. Kuze, J. Kvita, A. La Rosa, L. La Rotonda, C. Lacasta, F. Lacava, J. Lacey, H. Lacker, D. Lacour, V. R. Lacuesta, E. Ladygin, R. Lafaye, B. Laforge, T. Lagouri, S. Lai, H. Laier, L. Lambourne, S. Lammers, C. L. Lampen, W. Lampl, E. Lançon, U. Landgraf, M. P. J. Landon, V. S. Lang, A. J. Lankford, F. Lanni, K. Lantzsch, S. Laplace, C. Lapoire, J. F. Laporte, T. Lari, F. Lasagni Manghi, M. Lassnig, P. Laurelli, W. Lavrijsen, A. T. Law, P. Laycock, O. Le Dortz, E. Le Guirriec, E. Le Menedeu, T. LeCompte, F. Ledroit-Guillon, C. A. Lee, H. Lee, J. S. H. Lee, S. C. Lee, L. Lee, G. Lefebvre, M. Lefebvre, F. Legger, C. Leggett, A. Lehan, M. Lehmacher, G. Lehmann Miotto, X. Lei, W. A. Leight, A. Leisos, A. G. Leister, M. A. L. Leite, R. Leitner, D. Lellouch, B. Lemmer, K. J. C. Leney, T. Lenz, G. Lenzen, B. Lenzi, R. Leone, S. Leone, C. Leonidopoulos, S. Leontsinis, C. Leroy, C. G. Lester, C. M. Lester, M. Levchenko, J. Levêque, D. Levin, L. J. Levinson, M. Levy, A. Lewis, G. H. Lewis, A. M. Leyko, M. Leyton, B. Li, B. Li, H. Li, H. L. Li, L. Li, L. Li, S. Li, Y. Li, Z. Liang, H. Liao, B. Liberti, P. Lichard, K. Lie, J. Liebal, W. Liebig, C. Limbach, A. Limosani, S. C. Lin, T. H. Lin, F. Linde, B. E. Lindquist, J. T. Linnemann, E. Lipeles, A. Lipniacka, M. Lisovyi, T. M. Liss, D. Lissauer, A. Lister, A. M. Litke, B. Liu, D. Liu, J. B. Liu, K. Liu, L. Liu, M. Liu, M. Liu, Y. Liu, M. Livan, S. S. A. Livermore, A. Lleres, J. Llorente Merino, S. L. Lloyd, F. Lo Sterzo, E. Lobodzinska, P. Loch, W. S. Lockman, T. Loddenkoetter, F. K. Loebinger, A. E. Loevschall-Jensen, A. Loginov, T. Lohse, K. Lohwasser, M. Lokajicek, V. P. Lombardo, B. A. Long, J. D. Long, R. E. Long, L. Lopes, D. Lopez Mateos, B. Lopez Paredes, I. Lopez Paz, J. Lorenz, N. Lorenzo Martinez, M. Losada, P. Loscutoff, X. Lou, A. Lounis, J. Love, P. A. Love, A. J. Lowe, F. Lu, N. Lu, H. J. Lubatti, C. Luci, A. Lucotte, F. Luehring, W. Lukas, L. Luminari, O. Lundberg, B. Lund-Jensen, M. Lungwitz, D. Lynn, R. Lysak, E. Lytken, H. Ma, L. L. Ma, G. Maccarrone, A. Macchiolo, J. Machado Miguens, D. Macina, D. Madaffari, R. Madar, H. J. Maddocks, W. F. Mader, A. Madsen, M. Maeno, T. Maeno, A. Maevskiy, E. Magradze, K. Mahboubi, J. Mahlstedt, S. Mahmoud, C. Maiani, C. Maidantchik, A. A. Maier, A. Maio, S. Majewski, Y. Makida, N. Makovec, P. Mal, B. Malaescu, Pa. Malecki, V. P. Maleev, F. Malek, U. Mallik, D. Malon, C. Malone, S. Maltezos, V. M. Malyshev, S. Malyukov, J. Mamuzic, B. Mandelli, L. Mandelli, I. Mandić, R. Mandrysch, J. Maneira, A. Manfredini, L. Manhaes de Andrade Filho, J. A. Manjarres Ramos, A. Mann, P. M. Manning, A. Manousakis-Katsikakis, B. Mansoulie, R. Mantifel, L. Mapelli, L. March, J. F. Marchand, G. Marchiori, M. Marcisovsky, C. P. Marino, M. Marjanovic, C. N. Marques, F. Marroquim, S. P. Marsden, Z. Marshall, L. F. Marti, S. Marti-Garcia, B. Martin, B. Martin, T. A. Martin, V. J. Martin, B. Martin dit Latour, H. Martinez, M. Martinez, S. Martin-Haugh, A. C. Martyniuk, M. Marx, F. Marzano, A. Marzin, L. Masetti, T. Mashimo, R. Mashinistov, J. Masik, A. L. Maslennikov, I. Massa, L. Massa, N. Massol, P. Mastrandrea, A. Mastroberardino, T. Masubuchi, P. Mättig, J. Mattmann, J. Maurer, S. J. Maxfield, D. A. Maximov, R. Mazini, L. Mazzaferro, G. Mc Goldrick, S. P. Mc Kee, A. McCarn, R. L. McCarthy, T. G. McCarthy, N. A. McCubbin, K. W. McFarlane, J. A. Mcfayden, G. Mchedlidze, S. J. McMahon, R. A. McPherson, J. Mechnich, M. Medinnis, S. Meehan, S. Mehlhase, A. Mehta, K. Meier, C. Meineck, B. Meirose, C. Melachrinos, B. R. Mellado Garcia, F. Meloni, A. Mengarelli, S. Menke, E. Meoni, K. M. Mercurio, S. Mergelmeyer, N. Meric, P. Mermod, L. Merola, C. Meroni, F. S. Merritt, H. Merritt, A. Messina, J. Metcalfe, A. S. Mete, C. Meyer, C. Meyer, J-P. Meyer, J. Meyer, R. P. Middleton, S. Migas, L. Mijović, G. Mikenberg, M. Mikestikova, M. Mikuž, A. Milic, D. W. Miller, C. Mills, A. Milov, D. A. Milstead, D. Milstein, A. A. Minaenko, I. A. Minashvili, A. I. Mincer, B. Mindur, M. Mineev, Y. Ming, L. M. Mir, G. Mirabelli, T. Mitani, J. Mitrevski, V. A. Mitsou, S. Mitsui, A. Miucci, P. S. Miyagawa, J. U. Mjörnmark, T. Moa, K. Mochizuki, S. Mohapatra, W. Mohr, S. Molander, R. Moles-Valls, K. Mönig, C. Monini, J. Monk, E. Monnier, J. Montejo Berlingen, F. Monticelli, S. Monzani, R. W. Moore, N. Morange, D. Moreno, M. Moreno Llácer, P. Morettini, M. Morgenstern, M. Morii, S. Moritz, A. K. Morley, G. Mornacchi, J. D. Morris, L. Morvaj, H. G. Moser, M. Mosidze, J. Moss, K. Motohashi, R. Mount, E. Mountricha, S. V. Mouraviev, E. J. W. Moyse, S. Muanza, R. D. Mudd, F. Mueller, J. Mueller, K. Mueller, T. Mueller, T. Mueller, D. Muenstermann, Y. Munwes, J. A. Murillo Quijada, W. J. Murray, H. Musheghyan, E. Musto, A. G. Myagkov, M. Myska, O. Nackenhorst, J. Nadal, K. Nagai, R. Nagai, Y. Nagai, K. Nagano, A. Nagarkar, Y. Nagasaka, M. Nagel, A. M. Nairz, Y. Nakahama, K. Nakamura, T. Nakamura, I. Nakano, H. Namasivayam, G. Nanava, R. Narayan, T. Nattermann, T. Naumann, G. Navarro, R. Nayyar, H. A. Neal, P. Yu. Nechaeva, T. J. Neep, P. D. Nef, A. Negri, G. Negri, M. Negrini, S. Nektarijevic, A. Nelson, T. K. Nelson, S. Nemecek, P. Nemethy, A. A. Nepomuceno, M. Nessi, M. S. Neubauer, M. Neumann, R. M. Neves, P. Nevski, P. R. Newman, D. H. Nguyen, R. B. Nickerson, R. Nicolaidou, B. Nicquevert, J. Nielsen, N. Nikiforou, A. Nikiforov, V. Nikolaenko, I. Nikolic-Audit, K. Nikolics, K. Nikolopoulos, P. Nilsson, Y. Ninomiya, A. Nisati, R. Nisius, T. Nobe, L. Nodulman, M. Nomachi, I. Nomidis, S. Norberg, M. Nordberg, O. Novgorodova, S. Nowak, M. Nozaki, L. Nozka, K. Ntekas, G. Nunes Hanninger, T. Nunnemann, E. Nurse, F. Nuti, B. J. O’Brien, F. O’grady, D. C. O’Neil, V. O’Shea, F. G. Oakham, H. Oberlack, T. Obermann, J. Ocariz, A. Ochi, M. I. Ochoa, S. Oda, S. Odaka, H. Ogren, A. Oh, S. H. Oh, C. C. Ohm, H. Ohman, W. Okamura, H. Okawa, Y. Okumura, T. Okuyama, A. Olariu, A. G. Olchevski, S. A. Olivares Pino, D. Oliveira Damazio, E. Oliver Garcia, A. Olszewski, J. Olszowska, A. Onofre, P. U. E. Onyisi, C. J. Oram, M. J. Oreglia, Y. Oren, D. Orestano, N. Orlando, C. Oropeza Barrera, R. S. Orr, B. Osculati, R. Ospanov, G. Otero y Garzon, H. Otono, M. Ouchrif, E. A. Ouellette, F. Ould-Saada, A. Ouraou, K. P. Oussoren, Q. Ouyang, A. Ovcharova, M. Owen, V. E. Ozcan, N. Ozturk, K. Pachal, A. Pacheco Pages, C. Padilla Aranda, M. Pagáčová, S. Pagan Griso, E. Paganis, C. Pahl, F. Paige, P. Pais, K. Pajchel, G. Palacino, S. Palestini, M. Palka, D. Pallin, A. Palma, J. D. Palmer, Y. B. Pan, E. Panagiotopoulou, J. G. Panduro Vazquez, P. Pani, N. Panikashvili, S. Panitkin, D. Pantea, L. Paolozzi, Th. D. Papadopoulou, K. Papageorgiou, A. Paramonov, D. Paredes Hernandez, M. A. Parker, F. Parodi, J. A. Parsons, U. Parzefall, E. Pasqualucci, S. Passaggio, A. Passeri, F. Pastore, Fr. Pastore, G. Pásztor, S. Pataraia, N. D. Patel, J. R. Pater, S. Patricelli, T. Pauly, J. Pearce, L. E. Pedersen, M. Pedersen, S. Pedraza Lopez, R. Pedro, S. V. Peleganchuk, D. Pelikan, H. Peng, B. Penning, J. Penwell, D. V. Perepelitsa, E. Perez Codina, M. T. Pérez García-Estañ, V. Perez Reale, L. Perini, H. Pernegger, S. Perrella, R. Perrino, R. Peschke, V. D. Peshekhonov, K. Peters, R. F. Y. Peters, B. A. Petersen, T. C. Petersen, E. Petit, A. Petridis, C. Petridou, E. Petrolo, F. Petrucci, N. E. Pettersson, R. Pezoa, P. W. Phillips, G. Piacquadio, E. Pianori, A. Picazio, E. Piccaro, M. Piccinini, R. Piegaia, D. T. Pignotti, J. E. Pilcher, A. D. Pilkington, J. Pina, M. Pinamonti, A. Pinder, J. L. Pinfold, A. Pingel, B. Pinto, S. Pires, M. Pitt, C. Pizio, L. Plazak, M.-A. Pleier, V. Pleskot, E. Plotnikova, P. Plucinski, S. Poddar, F. Podlyski, R. Poettgen, L. Poggioli, D. Pohl, M. Pohl, G. Polesello, A. Policicchio, R. Polifka, A. Polini, C. S. Pollard, V. Polychronakos, K. Pommès, L. Pontecorvo, B. G. Pope, G. A. Popeneciu, D. S. Popovic, A. Poppleton, X. Portell Bueso, S. Pospisil, K. Potamianos, I. N. Potrap, C. J. Potter, C. T. Potter, G. Poulard, J. Poveda, V. Pozdnyakov, P. Pralavorio, A. Pranko, S. Prasad, R. Pravahan, S. Prell, D. Price, J. Price, L. E. Price, D. Prieur, M. Primavera, M. Proissl, K. Prokofiev, F. Prokoshin, E. Protopapadaki, S. Protopopescu, J. Proudfoot, M. Przybycien, H. Przysiezniak, E. Ptacek, D. Puddu, E. Pueschel, D. Puldon, M. Purohit, P. Puzo, J. Qian, G. Qin, Y. Qin, A. Quadt, D. R. Quarrie, W. B. Quayle, M. Queitsch-Maitland, D. Quilty, A. Qureshi, V. Radeka, V. Radescu, S. K. Radhakrishnan, P. Radloff, P. Rados, F. Ragusa, G. Rahal, S. Rajagopalan, M. Rammensee, A. S. Randle-Conde, C. Rangel-Smith, K. Rao, F. Rauscher, T. C. Rave, T. Ravenscroft, M. Raymond, A. L. Read, N. P. Readioff, D. M. Rebuzzi, A. Redelbach, G. Redlinger, R. Reece, K. Reeves, L. Rehnisch, H. Reisin, M. Relich, C. Rembser, H. Ren, Z. L. Ren, A. Renaud, M. Rescigno, S. Resconi, O. L. Rezanova, P. Reznicek, R. Rezvani, R. Richter, M. Ridel, P. Rieck, J. Rieger, M. Rijssenbeek, A. Rimoldi, L. Rinaldi, E. Ritsch, I. Riu, F. Rizatdinova, E. Rizvi, S. H. Robertson, A. Robichaud-Veronneau, D. Robinson, J. E. M. Robinson, A. Robson, C. Roda, L. Rodrigues, S. Roe, O. Røhne, S. Rolli, A. Romaniouk, M. Romano, E. Romero Adam, N. Rompotis, M. Ronzani, L. Roos, E. Ros, S. Rosati, K. Rosbach, M. Rose, P. Rose, P. L. Rosendahl, O. Rosenthal, V. Rossetti, E. Rossi, L. P. Rossi, R. Rosten, M. Rotaru, I. Roth, J. Rothberg, D. Rousseau, C. R. Royon, A. Rozanov, Y. Rozen, X. Ruan, F. Rubbo, I. Rubinskiy, V. I. Rud, C. Rudolph, M. S. Rudolph, F. Rühr, A. Ruiz-Martinez, Z. Rurikova, N. A. Rusakovich, A. Ruschke, J. P. Rutherfoord, N. Ruthmann, Y. F. Ryabov, M. Rybar, G. Rybkin, N. C. Ryder, A. F. Saavedra, S. Sacerdoti, A. Saddique, I. Sadeh, H. F-W. Sadrozinski, R. Sadykov, F. Safai Tehrani, H. Sakamoto, Y. Sakurai, G. Salamanna, A. Salamon, M. Saleem, D. Salek, P. H. Sales De Bruin, D. Salihagic, A. Salnikov, J. Salt, D. Salvatore, F. Salvatore, A. Salvucci, A. Salzburger, D. Sampsonidis, A. Sanchez, J. Sánchez, V. Sanchez Martinez, H. Sandaker, R. L. Sandbach, H. G. Sander, M. P. Sanders, M. Sandhoff, T. Sandoval, C. Sandoval, R. Sandstroem, D. P. C. Sankey, A. Sansoni, C. Santoni, R. Santonico, H. Santos, I. Santoyo Castillo, K. Sapp, A. Sapronov, J. G. Saraiva, B. Sarrazin, G. Sartisohn, O. Sasaki, Y. Sasaki, G. Sauvage, E. Sauvan, P. Savard, D. O. Savu, C. Sawyer, L. Sawyer, D. H. Saxon, J. Saxon, C. Sbarra, A. Sbrizzi, T. Scanlon, D. A. Scannicchio, M. Scarcella, V. Scarfone, J. Schaarschmidt, P. Schacht, D. Schaefer, R. Schaefer, S. Schaepe, S. Schaetzel, U. Schäfer, A. C. Schaffer, D. Schaile, R. D. Schamberger, V. Scharf, V. A. Schegelsky, D. Scheirich, M. Schernau, M. I. Scherzer, C. Schiavi, J. Schieck, C. Schillo, M. Schioppa, S. Schlenker, E. Schmidt, K. Schmieden, C. Schmitt, S. Schmitt, B. Schneider, Y. J. Schnellbach, U. Schnoor, L. Schoeffel, A. Schoening, B. D. Schoenrock, A. L. S. Schorlemmer, M. Schott, D. Schouten, J. Schovancova, S. Schramm, M. Schreyer, C. Schroeder, N. Schuh, M. J. Schultens, H.-C. Schultz-Coulon, H. Schulz, M. Schumacher, B. A. Schumm, Ph. Schune, C. Schwanenberger, A. Schwartzman, T. A. Schwarz, Ph. Schwegler, Ph. Schwemling, R. Schwienhorst, J. Schwindling, T. Schwindt, M. Schwoerer, F. G. Sciacca, E. Scifo, G. Sciolla, W. G. Scott, F. Scuri, F. Scutti, J. Searcy, G. Sedov, E. Sedykh, S. C. Seidel, A. Seiden, F. Seifert, J. M. Seixas, G. Sekhniaidze, S. J. Sekula, K. E. Selbach, D. M. Seliverstov, G. Sellers, N. Semprini-Cesari, C. Serfon, L. Serin, L. Serkin, T. Serre, R. Seuster, H. Severini, T. Sfiligoj, F. Sforza, A. Sfyrla, E. Shabalina, M. Shamim, L. Y. Shan, R. Shang, J. T. Shank, M. Shapiro, P. B. Shatalov, K. Shaw, C. Y. Shehu, P. Sherwood, L. Shi, S. Shimizu, C. O. Shimmin, M. Shimojima, M. Shiyakova, A. Shmeleva, M. J. Shochet, D. Short, S. Shrestha, E. Shulga, M. A. Shupe, S. Shushkevich, P. Sicho, O. Sidiropoulou, D. Sidorov, A. Sidoti, F. Siegert, Dj. Sijacki, J. Silva, Y. Silver, D. Silverstein, S. B. Silverstein, V. Simak, O. Simard, Lj. Simic, S. Simion, E. Simioni, B. Simmons, R. Simoniello, M. Simonyan, P. Sinervo, N. B. Sinev, V. Sipica, G. Siragusa, A. Sircar, A. N. Sisakyan, S. Yu. Sivoklokov, J. Sjölin, T. B. Sjursen, H. P. Skottowe, K. Yu. Skovpen, P. Skubic, M. Slater, T. Slavicek, K. Sliwa, V. Smakhtin, B. H. Smart, L. Smestad, S. Yu. Smirnov, Y. Smirnov, L. N. Smirnova, O. Smirnova, K. M. Smith, M. Smizanska, K. Smolek, A. A. Snesarev, G. Snidero, S. Snyder, R. Sobie, F. Socher, A. Soffer, D. A. Soh, C. A. Solans, M. Solar, J. Solc, E. Yu. Soldatov, U. Soldevila, A. A. Solodkov, A. Soloshenko, O. V. Solovyanov, V. Solovyev, P. Sommer, H. Y. Song, N. Soni, A. Sood, A. Sopczak, B. Sopko, V. Sopko, V. Sorin, M. Sosebee, R. Soualah, P. Soueid, A. M. Soukharev, D. South, S. Spagnolo, F. Spanò, W. R. Spearman, F. Spettel, R. Spighi, G. Spigo, L. A. Spiller, M. Spousta, T. Spreitzer, B. Spurlock, R. D. St. Denis, S. Staerz, J. Stahlman, R. Stamen, S. Stamm, E. Stanecka, R. W. Stanek, C. Stanescu, M. Stanescu-Bellu, M. M. Stanitzki, S. Stapnes, E. A. Starchenko, J. Stark, P. Staroba, P. Starovoitov, R. Staszewski, P. Stavina, P. Steinberg, B. Stelzer, H. J. Stelzer, O. Stelzer-Chilton, H. Stenzel, S. Stern, G. A. Stewart, J. A. Stillings, M. C. Stockton, M. Stoebe, G. Stoicea, P. Stolte, S. Stonjek, A. R. Stradling, A. Straessner, M. E. Stramaglia, J. Strandberg, S. Strandberg, A. Strandlie, E. Strauss, M. Strauss, P. Strizenec, R. Ströhmer, D. M. Strom, R. Stroynowski, A. Struebig, S. A. Stucci, B. Stugu, N. A. Styles, D. Su, J. Su, R. Subramaniam, A. Succurro, Y. Sugaya, C. Suhr, M. Suk, V. V. Sulin, S. Sultansoy, T. Sumida, S. Sun, X. Sun, J. E. Sundermann, K. Suruliz, G. Susinno, M. R. Sutton, Y. Suzuki, M. Svatos, S. Swedish, M. Swiatlowski, I. Sykora, T. Sykora, D. Ta, C. Taccini, K. Tackmann, J. Taenzer, A. Taffard, R. Tafirout, N. Taiblum, H. Takai, R. Takashima, H. Takeda, T. Takeshita, Y. Takubo, M. Talby, A. A. Talyshev, J. Y. C. Tam, K. G. Tan, J. Tanaka, R. Tanaka, S. Tanaka, S. Tanaka, A. J. Tanasijczuk, B. B. Tannenwald, N. Tannoury, S. Tapprogge, S. Tarem, F. Tarrade, G. F. Tartarelli, P. Tas, M. Tasevsky, T. Tashiro, E. Tassi, A. Tavares Delgado, Y. Tayalati, F. E. Taylor, G. N. Taylor, W. Taylor, F. A. Teischinger, M. Teixeira Dias Castanheira, P. Teixeira-Dias, K. K. Temming, H. Ten Kate, P. K. Teng, J. J. Teoh, S. Terada, K. Terashi, J. Terron, S. Terzo, M. Testa, R. J. Teuscher, J. Therhaag, T. Theveneaux-Pelzer, J. P. Thomas, J. Thomas-Wilsker, E. N. Thompson, P. D. Thompson, P. D. Thompson, R. J. Thompson, A. S. Thompson, L. A. Thomsen, E. Thomson, M. Thomson, W. M. Thong, R. P. Thun, F. Tian, M. J. Tibbetts, V. O. Tikhomirov, Yu. A. Tikhonov, S. Timoshenko, E. Tiouchichine, P. Tipton, S. Tisserant, T. Todorov, S. Todorova-Nova, B. Toggerson, J. Tojo, S. Tokár, K. Tokushuku, K. Tollefson, E. Tolley, L. Tomlinson, M. Tomoto, L. Tompkins, K. Toms, N. D. Topilin, E. Torrence, H. Torres, E. Torró Pastor, J. Toth, F. Touchard, D. R. Tovey, H. L. Tran, T. Trefzger, L. Tremblet, A. Tricoli, I. M. Trigger, S. Trincaz-Duvoid, M. F. Tripiana, W. Trischuk, B. Trocmé, C. Troncon, M. Trottier-McDonald, M. Trovatelli, P. True, M. Trzebinski, A. Trzupek, C. Tsarouchas, J. C-L. Tseng, P. V. Tsiareshka, D. Tsionou, G. Tsipolitis, N. Tsirintanis, S. Tsiskaridze, V. Tsiskaridze, E. G. Tskhadadze, I. I. Tsukerman, V. Tsulaia, S. Tsuno, D. Tsybychev, A. Tudorache, V. Tudorache, A. N. Tuna, S. A. Tupputi, S. Turchikhin, D. Turecek, I. Turk Cakir, R. Turra, P. M. Tuts, A. Tykhonov, M. Tylmad, M. Tyndel, K. Uchida, I. Ueda, R. Ueno, M. Ughetto, M. Ugland, M. Uhlenbrock, F. Ukegawa, G. Unal, A. Undrus, G. Unel, F. C. Ungaro, Y. Unno, C. Unverdorben, D. Urbaniec, P. Urquijo, G. Usai, A. Usanova, L. Vacavant, V. Vacek, B. Vachon, N. Valencic, S. Valentinetti, A. Valero, L. Valery, S. Valkar, E. Valladolid Gallego, S. Vallecorsa, J. A. Valls Ferrer, W. Van Den Wollenberg, P. C. Van Der Deijl, R. van der Geer, H. van der Graaf, R. Van Der Leeuw, D. van der Ster, N. van Eldik, P. van Gemmeren, J. Van Nieuwkoop, I. van Vulpen, M. C. van Woerden, M. Vanadia, W. Vandelli, R. Vanguri, A. Vaniachine, P. Vankov, F. Vannucci, G. Vardanyan, R. Vari, E. W. Varnes, T. Varol, D. Varouchas, A. Vartapetian, K. E. Varvell, F. Vazeille, T. Vazquez Schroeder, J. Veatch, F. Veloso, S. Veneziano, A. Ventura, D. Ventura, M. Venturi, N. Venturi, A. Venturini, V. Vercesi, M. Verducci, W. Verkerke, J. C. Vermeulen, A. Vest, M. C. Vetterli, O. Viazlo, I. Vichou, T. Vickey, O. E. Vickey Boeriu, G. H. A. Viehhauser, S. Viel, R. Vigne, M. Villa, M. Villaplana Perez, E. Vilucchi, M. G. Vincter, V. B. Vinogradov, J. Virzi, I. Vivarelli, F. Vives Vaque, S. Vlachos, D. Vladoiu, M. Vlasak, A. Vogel, M. Vogel, P. Vokac, G. Volpi, M. Volpi, H. von der Schmitt, H. von Radziewski, E. von Toerne, V. Vorobel, K. Vorobev, M. Vos, R. Voss, J. H. Vossebeld, N. Vranjes, M. Vranjes Milosavljevic, V. Vrba, M. Vreeswijk, T. Vu Anh, R. Vuillermet, I. Vukotic, Z. Vykydal, P. Wagner, W. Wagner, H. Wahlberg, S. Wahrmund, J. Wakabayashi, J. Walder, R. Walker, W. Walkowiak, R. Wall, P. Waller, B. Walsh, C. Wang, C. Wang, F. Wang, H. Wang, H. Wang, J. Wang, J. Wang, K. Wang, R. Wang, S. M. Wang, T. Wang, X. Wang, C. Wanotayaroj, A. Warburton, C. P. Ward, D. R. Wardrope, M. Warsinsky, A. Washbrook, C. Wasicki, P. M. Watkins, A. T. Watson, I. J. Watson, M. F. Watson, G. Watts, S. Watts, B. M. Waugh, S. Webb, M. S. Weber, S. W. Weber, J. S. Webster, A. R. Weidberg, P. Weigell, B. Weinert, J. Weingarten, C. Weiser, H. Weits, P. S. Wells, T. Wenaus, D. Wendland, Z. Weng, T. Wengler, S. Wenig, N. Wermes, M. Werner, P. Werner, M. Wessels, J. Wetter, K. Whalen, A. White, M. J. White, R. White, S. White, D. Whiteson, D. Wicke, F. J. Wickens, W. Wiedenmann, M. Wielers, P. Wienemann, C. Wiglesworth, L. A. M. Wiik-Fuchs, P. A. Wijeratne, A. Wildauer, M. A. Wildt, H. G. Wilkens, J. Z. Will, H. H. Williams, S. Williams, C. Willis, S. Willocq, A. Wilson, J. A. Wilson, I. Wingerter-Seez, F. Winklmeier, B. T. Winter, M. Wittgen, T. Wittig, J. Wittkowski, S. J. Wollstadt, M. W. Wolter, H. Wolters, B. K. Wosiek, J. Wotschack, M. J. Woudstra, K. W. Wozniak, M. Wright, M. Wu, S. L. Wu, X. Wu, Y. Wu, E. Wulf, T. R. Wyatt, B. M. Wynne, S. Xella, M. Xiao, D. Xu, L. Xu, B. Yabsley, S. Yacoob, R. Yakabe, M. Yamada, H. Yamaguchi, Y. Yamaguchi, A. Yamamoto, K. Yamamoto, S. Yamamoto, T. Yamamura, T. Yamanaka, K. Yamauchi, Y. Yamazaki, Z. Yan, H. Yang, H. Yang, U. K. Yang, Y. Yang, S. Yanush, L. Yao, W-M. Yao, Y. Yasu, E. Yatsenko, K. H. Yau Wong, J. Ye, S. Ye, I. Yeletskikh, A. L. Yen, E. Yildirim, M. Yilmaz, R. Yoosoofmiya, K. Yorita, R. Yoshida, K. Yoshihara, C. Young, C. J. S. Young, S. Youssef, D. R. Yu, J. Yu, J. M. Yu, J. Yu, L. Yuan, A. Yurkewicz, I. Yusuff, B. Zabinski, R. Zaidan, A. M. Zaitsev, A. Zaman, S. Zambito, L. Zanello, D. Zanzi, C. Zeitnitz, M. Zeman, A. Zemla, K. Zengel, O. Zenin, T. Ženiš, D. Zerwas, G. Zevi della Porta, D. Zhang, F. Zhang, H. Zhang, J. Zhang, L. Zhang, X. Zhang, Z. Zhang, Z. Zhao, A. Zhemchugov, J. Zhong, B. Zhou, L. Zhou, N. Zhou, C. G. Zhu, H. Zhu, J. Zhu, Y. Zhu, X. Zhuang, K. Zhukov, A. Zibell, D. Zieminska, N. I. Zimine, C. Zimmermann, R. Zimmermann, S. Zimmermann, S. Zimmermann, Z. Zinonos, M. Ziolkowski, G. Zobernig, A. Zoccoli, M. zur Nedden, G. Zurzolo, V. Zutshi, L. Zwalinski

**Affiliations:** 1 Department of Physics, University of Adelaide, Adelaide, Australia; 2 Physics Department, SUNY Albany, Albany, NY USA; 3 Department of Physics, University of Alberta, Edmonton, AB Canada; 4 Department of Physics, Ankara University, Ankara, Turkey; Department of Physics, Gazi University, Ankara, Turkey; Division of Physics, TOBB University of Economics and Technology, Ankara, Turkey; Turkish Atomic Energy Authority, Ankara, Turkey; 5 LAPP, CNRS/IN2P3 and Université de Savoie, Annecy-le-Vieux, France; 6 High Energy Physics Division, Argonne National Laboratory, Argonne, IL USA; 7 Department of Physics, University of Arizona, Tucson, AZ USA; 8 Department of Physics, The University of Texas at Arlington, Arlington, TX USA; 9 Physics Department, University of Athens, Athens, Greece; 10 Physics Department, National Technical University of Athens, Zografou, Greece; 11 Institute of Physics, Azerbaijan Academy of Sciences, Baku, Azerbaijan; 12 Institut de Física d’Altes Energies and Departament de Física de la Universitat Autònoma de Barcelona, Barcelona, Spain; 13 Institute of Physics, University of Belgrade, Belgrade, Serbia; Vinca Institute of Nuclear Sciences, University of Belgrade, Belgrade, Serbia; 14 Department for Physics and Technology, University of Bergen, Bergen, Norway; 15 Physics Division, Lawrence Berkeley National Laboratory and University of California, Berkeley, CA USA; 16 Department of Physics, Humboldt University, Berlin, Germany; 17 Albert Einstein Center for Fundamental Physics and Laboratory for High Energy Physics, University of Bern, Bern, Switzerland; 18 School of Physics and Astronomy, University of Birmingham, Birmingham, UK; 19 Department of Physics, Bogazici University, Istanbul, Turkey; Department of Physics, Dogus University, Istanbul, Turkey; Department of Physics Engineering, Gaziantep University, Gaziantep, Turkey; 20 INFN Sezione di Bologna, Bologna, Italy; Dipartimento di Fisica e Astronomia, Università di Bologna, Bologna, Italy; 21 Physikalisches Institut, University of Bonn, Bonn, Germany; 22 Department of Physics, Boston University, Boston, MA USA; 23 Department of Physics, Brandeis University, Waltham, MA USA; 24 Universidade Federal do Rio De Janeiro COPPE/EE/IF, Rio de Janeiro, Brazil; Federal University of Juiz de Fora (UFJF), Juiz de Fora, Brazil; Federal University of Sao Joao del Rei (UFSJ), Sao Joao del Rei, Brazil; Instituto de Fisica, Universidade de Sao Paulo, São Paulo, Brazil; 25 Physics Department, Brookhaven National Laboratory, Upton, NY USA; 26 National Institute of Physics and Nuclear Engineering, Bucharest, Romania; Physics Department, National Institute for Research and Development of Isotopic and Molecular Technologies, Cluj Napoca, Romania; University Politehnica Bucharest, Bucharest, Romania; West University in Timisoara, Timisoara, Romania; 27 Departamento de Física, Universidad de Buenos Aires, Buenos Aires, Argentina; 28 Cavendish Laboratory, University of Cambridge, Cambridge, UK; 29 Department of Physics, Carleton University, Ottawa, ON Canada; 30 CERN, Geneva, Switzerland; 31 Enrico Fermi Institute, University of Chicago, Chicago, IL USA; 32 Departamento de Física, Pontificia Universidad Católica de Chile, Santiago, Chile; Departamento de Física, Universidad Técnica Federico Santa María, Valparaiso, Chile; 33 Institute of High Energy Physics, Chinese Academy of Sciences, Beijing, China; Department of Modern Physics, University of Science and Technology of China, Hefei, Anhui, China; Department of Physics, Nanjing University, Nanjing, Jiangsu, China; School of Physics, Shandong University, Jinan, Shandong, China; Physics Department, Shanghai Jiao Tong University, Shanghai, China; 34 Laboratoire de Physique Corpusculaire, Clermont Université and Université Blaise Pascal and CNRS/IN2P3, Clermont-Ferrand, France; 35 Nevis Laboratory, Columbia University, Irvington, NY USA; 36 Niels Bohr Institute, University of Copenhagen, Copenhagen, Denmark; 37 INFN Gruppo Collegato di Cosenza, Laboratori Nazionali di Frascati, Frascati, Italy; Dipartimento di Fisica, Università della Calabria, Rende, Italy; 38 Faculty of Physics and Applied Computer Science, AGH University of Science and Technology, Kraków, Poland; Marian Smoluchowski Institute of Physics, Jagiellonian University, Kraków, Poland; 39 The Henryk Niewodniczanski Institute of Nuclear Physics, Polish Academy of Sciences, Kraków, Poland; 40 Physics Department, Southern Methodist University, Dallas, TX USA; 41 Physics Department, University of Texas at Dallas, Richardson, TX USA; 42 DESY, Hamburg and Zeuthen, Germany; 43 Institut für Experimentelle Physik IV, Technische Universität Dortmund, Dortmund, Germany; 44 Institut für Kern- und Teilchenphysik, Technische Universität Dresden, Dresden, Germany; 45 Department of Physics, Duke University, Durham, NC USA; 46 SUPA-School of Physics and Astronomy, University of Edinburgh, Edinburgh, UK; 47 INFN Laboratori Nazionali di Frascati, Frascati, Italy; 48 Fakultät für Mathematik und Physik, Albert-Ludwigs-Universität, Freiburg, Germany; 49 Section de Physique, Université de Genève, Geneva, Switzerland; 50 INFN Sezione di Genova, Genoa, Italy; Dipartimento di Fisica, Università di Genova, Genova, Italy; 51 E. Andronikashvili Institute of Physics, Iv. Javakhishvili Tbilisi State University, Tbilisi, Georgia; High Energy Physics Institute, Tbilisi State University, Tbilisi, Georgia; 52 II Physikalisches Institut, Justus-Liebig-Universität Giessen, Giessen, Germany; 53 SUPA-School of Physics and Astronomy, University of Glasgow, Glasgow, UK; 54 II Physikalisches Institut, Georg-August-Universität, Göttingen, Germany; 55 Laboratoire de Physique Subatomique et de Cosmologie, Université Grenoble-Alpes, CNRS/IN2P3, Grenoble, France; 56 Department of Physics, Hampton University, Hampton, VA USA; 57 Laboratory for Particle Physics and Cosmology, Harvard University, Cambridge, MA USA; 58 Kirchhoff-Institut für Physik, Ruprecht-Karls-Universität Heidelberg, Heidelberg, Germany; Physikalisches Institut, Ruprecht-Karls-Universität Heidelberg, Heidelberg, Germany; ZITI Institut für technische Informatik, Ruprecht-Karls-Universität Heidelberg, Mannheim, Germany; 59 Faculty of Applied Information Science, Hiroshima Institute of Technology, Hiroshima, Japan; 60 Department of Physics, Indiana University, Bloomington, IN USA; 61 Institut für Astro- und Teilchenphysik, Leopold-Franzens-Universität, Innsbruck, Austria; 62 University of Iowa, Iowa City, IA USA; 63 Department of Physics and Astronomy, Iowa State University, Ames, IA USA; 64 Joint Institute for Nuclear Research, JINR Dubna, Dubna, Russia; 65 KEK, High Energy Accelerator Research Organization, Tsukuba, Japan; 66 Graduate School of Science, Kobe University, Kobe, Japan; 67 Faculty of Science, Kyoto University, Kyoto, Japan; 68 Kyoto University of Education, Kyoto, Japan; 69 Department of Physics, Kyushu University, Fukuoka, Japan; 70 Instituto de Física La Plata, Universidad Nacional de La Plata and CONICET, La Plata, Argentina; 71 Physics Department, Lancaster University, Lancaster, UK; 72 INFN Sezione di Lecce, Lecce, Italy; Dipartimento di Matematica e Fisica, Università del Salento, Lecce, Italy; 73 Oliver Lodge Laboratory, University of Liverpool, Liverpool, UK; 74 Department of Physics, Jožef Stefan Institute and University of Ljubljana, Ljubljana, Slovenia; 75 School of Physics and Astronomy, Queen Mary University of London, London, UK; 76 Department of Physics, Royal Holloway University of London, Surrey, UK; 77 Department of Physics and Astronomy, University College London, London, UK; 78 Louisiana Tech University, Ruston, LA USA; 79 Laboratoire de Physique Nucléaire et de Hautes Energies, UPMC and Université Paris-Diderot and CNRS/IN2P3, Paris, France; 80 Fysiska institutionen, Lunds universitet, Lund, Sweden; 81 Departamento de Fisica Teorica C-15, Universidad Autonoma de Madrid, Madrid, Spain; 82 Institut für Physik, Universität Mainz, Mainz, Germany; 83 School of Physics and Astronomy, University of Manchester, Manchester, UK; 84 CPPM, Aix-Marseille Université and CNRS/IN2P3, Marseille, France; 85 Department of Physics, University of Massachusetts, Amherst, MA USA; 86 Department of Physics, McGill University, Montreal, QC Canada; 87 School of Physics, University of Melbourne, Parkville, VIC Australia; 88 Department of Physics, The University of Michigan, Ann Arbor, MI USA; 89 Department of Physics and Astronomy, Michigan State University, East Lansing, MI USA; 90 INFN Sezione di Milano, Milan, Italy; Dipartimento di Fisica, Università di Milano, Milan, Italy; 91 B.I. Stepanov Institute of Physics, National Academy of Sciences of Belarus, Minsk, Republic of Belarus; 92 National Scientific and Educational Centre for Particle and High Energy Physics, Minsk, Republic of Belarus; 93 Department of Physics, Massachusetts Institute of Technology, Cambridge, MA USA; 94 Group of Particle Physics, University of Montreal, Montreal, QC Canada; 95 P.N. Lebedev Institute of Physics, Academy of Sciences, Moscow, Russia; 96 Institute for Theoretical and Experimental Physics (ITEP), Moscow, Russia; 97 Moscow Engineering and Physics Institute (MEPhI), Moscow, Russia; 98 D.V. Skobeltsyn Institute of Nuclear Physics, M.V. Lomonosov Moscow State University, Moscow, Russia; 99 Fakultät für Physik, Ludwig-Maximilians-Universität München, Munich, Germany; 100 Max-Planck-Institut für Physik (Werner-Heisenberg-Institut), Munich, Germany; 101 Nagasaki Institute of Applied Science, Nagasaki, Japan; 102 Graduate School of Science and Kobayashi-Maskawa Institute, Nagoya University, Nagoya, Japan; 103 INFN Sezione di Napoli, Naples, Italy; Dipartimento di Fisica, Università di Napoli, Naples, Italy; 104 Department of Physics and Astronomy, University of New Mexico, Albuquerque, NM USA; 105 Institute for Mathematics, Astrophysics and Particle Physics, Radboud University Nijmegen/Nikhef, Nijmegen, The Netherlands; 106 Nikhef National Institute for Subatomic Physics and University of Amsterdam, Amsterdam, The Netherlands; 107 Department of Physics, Northern Illinois University, DeKalb, IL USA; 108 Budker Institute of Nuclear Physics, SB RAS, Novosibirsk, Russia; 109 Department of Physics, New York University, New York, NY USA; 110 Ohio State University, Columbus, OH USA; 111 Faculty of Science, Okayama University, Okayama, Japan; 112 Homer L. Dodge Department of Physics and Astronomy, University of Oklahoma, Norman, OK USA; 113 Department of Physics, Oklahoma State University, Stillwater, OK USA; 114 Palacký University, RCPTM, Olomouc, Czech Republic; 115 Center for High Energy Physics, University of Oregon, Eugene, OR USA; 116 LAL, Université Paris-Sud and CNRS/IN2P3, Orsay, France; 117 Graduate School of Science, Osaka University, Osaka, Japan; 118 Department of Physics, University of Oslo, Oslo, Norway; 119 Department of Physics, Oxford University, Oxford, UK; 120 INFN Sezione di Pavia, Pavia, Italy; Dipartimento di Fisica, Università di Pavia, Pavia, Italy; 121 Department of Physics, University of Pennsylvania, Philadelphia, PA USA; 122 Petersburg Nuclear Physics Institute, Gatchina, Russia; 123 INFN Sezione di Pisa, Pisa, Italy; Dipartimento di Fisica E. Fermi, Università di Pisa, Pisa, Italy; 124 Department of Physics and Astronomy, University of Pittsburgh, Pittsburgh, PA USA; 125 Laboratorio de Instrumentacao e Fisica Experimental de Particulas-LIP, Lisbon, Portugal; Faculdade de Ciências, Universidade de Lisboa, Lisbon, Portugal; Department of Physics, University of Coimbra, Coimbra, Portugal; Centro de Física Nuclear da Universidade de Lisboa, Lisbon, Portugal; Departamento de Fisica, Universidade do Minho, Braga, Portugal; Departamento de Fisica Teorica y del Cosmos and CAFPE, Universidad de Granada, Granada, Spain; Dep Fisica and CEFITEC of Faculdade de Ciencias e Tecnologia, Universidade Nova de Lisboa, Caparica, Portugal; 126 Institute of Physics, Academy of Sciences of the Czech Republic, Prague, Czech Republic; 127 Czech Technical University in Prague, Prague, Czech Republic; 128 Faculty of Mathematics and Physics, Charles University in Prague, Prague, Czech Republic; 129 State Research Center Institute for High Energy Physics, Protvino, Russia; 130 Particle Physics Department, Rutherford Appleton Laboratory, Didcot, UK; 131 Physics Department, University of Regina, Regina, SK Canada; 132 Ritsumeikan University, Kusatsu, Shiga Japan; 133 INFN Sezione di Roma, Rome, Italy; Dipartimento di Fisica, Sapienza Università di Roma, Rome, Italy; 134 INFN Sezione di Roma Tor Vergata, Rome, Italy; Dipartimento di Fisica, Università di Roma Tor Vergata, Rome, Italy; 135 INFN Sezione di Roma Tre, Rome, Italy; Dipartimento di Matematica e Fisica, Università Roma Tre, Rome, Italy; 136 Faculté des Sciences Ain Chock, Réseau Universitaire de Physique des Hautes Energies-Université Hassan II, Casablanca, Morocco; Centre National de l’Energie des Sciences Techniques Nucleaires, Rabat, Morocco; Faculté des Sciences Semlalia, Université Cadi Ayyad, LPHEA-Marrakech, Marrakech, Morocco; Faculté des Sciences, Université Mohamed Premier and LPTPM, Oujda, Morocco; Faculté des Sciences, Université Mohammed V-Agdal, Rabat, Morocco; 137 DSM/IRFU (Institut de Recherches sur les Lois Fondamentales de l’Univers), CEA Saclay (Commissariat à l’Energie Atomique et aux Energies Alternatives), Gif-sur-Yvette, France; 138 Santa Cruz Institute for Particle Physics, University of California Santa Cruz, Santa Cruz, CA USA; 139 Department of Physics, University of Washington, Seattle, WA USA; 140 Department of Physics and Astronomy, University of Sheffield, Sheffield, UK; 141 Department of Physics, Shinshu University, Nagano, Japan; 142 Fachbereich Physik, Universität Siegen, Siegen, Germany; 143 Department of Physics, Simon Fraser University, Burnaby, BC Canada; 144 SLAC National Accelerator Laboratory, Stanford, CA USA; 145 Faculty of Mathematics, Physics and Informatics, Comenius University, Bratislava, Slovak Republic; Department of Subnuclear Physics, Institute of Experimental Physics of the Slovak Academy of Sciences, Kosice, Slovak Republic; 146 Department of Physics, University of Cape Town, Cape Town, South Africa; Department of Physics, University of Johannesburg, Johannesburg, South Africa; School of Physics, University of the Witwatersrand, Johannesburg, South Africa; 147 Department of Physics, Stockholm University, Stockholm, Sweden; The Oskar Klein Centre, Stockholm, Sweden; 148 Physics Department, Royal Institute of Technology, Stockholm, Sweden; 149 Departments of Physics and Astronomy and Chemistry, Stony Brook University, Stony Brook, NY USA; 150 Department of Physics and Astronomy, University of Sussex, Brighton, UK; 151 School of Physics, University of Sydney, Sydney, Australia; 152 Institute of Physics, Academia Sinica, Taipei, Taiwan; 153 Department of Physics, Technion: Israel Institute of Technology, Haifa, Israel; 154 Raymond and Beverly Sackler School of Physics and Astronomy, Tel Aviv University, Tel Aviv, Israel; 155 Department of Physics, Aristotle University of Thessaloniki, Thessaloniki, Greece; 156 International Center for Elementary Particle Physics and Department of Physics, The University of Tokyo, Tokyo, Japan; 157 Graduate School of Science and Technology, Tokyo Metropolitan University, Tokyo, Japan; 158 Department of Physics, Tokyo Institute of Technology, Tokyo, Japan; 159 Department of Physics, University of Toronto, Toronto, ON Canada; 160 TRIUMF, Vancouver, BC, Canada; Department of Physics and Astronomy, York University, Toronto, ON Canada; 161 Faculty of Pure and Applied Sciences, University of Tsukuba, Tsukuba, Japan; 162 Department of Physics and Astronomy, Tufts University, Medford, MA USA; 163 Centro de Investigaciones, Universidad Antonio Narino, Bogota, Colombia; 164 Department of Physics and Astronomy, University of California Irvine, Irvine, CA USA; 165 INFN Gruppo Collegato di Udine, Sezione di Trieste, Udine, Italy; ICTP, Trieste, Italy; Dipartimento di Chimica, Fisica e Ambiente, Università di Udine, Udine, Italy; 166 Department of Physics, University of Illinois, Urbana, IL USA; 167 Department of Physics and Astronomy, University of Uppsala, Uppsala, Sweden; 168 Instituto de Física Corpuscular (IFIC) and Departamento de Física Atómica, Molecular y Nuclear and Departamento de Ingeniería Electrónica and Instituto de Microelectrónica de Barcelona (IMB-CNM), University of Valencia and CSIC, Valencia, Spain; 169 Department of Physics, University of British Columbia, Vancouver, BC Canada; 170 Department of Physics and Astronomy, University of Victoria, Victoria, BC Canada; 171 Department of Physics, University of Warwick, Coventry, UK; 172 Waseda University, Tokyo, Japan; 173 Department of Particle Physics, The Weizmann Institute of Science, Rehovot, Israel; 174 Department of Physics, University of Wisconsin, Madison, WI USA; 175 Fakultät für Physik und Astronomie, Julius-Maximilians-Universität, Würzburg, Germany; 176 Fachbereich C Physik, Bergische Universität Wuppertal, Wuppertal, Germany; 177 Department of Physics, Yale University, New Haven, CT USA; 178 Yerevan Physics Institute, Yerevan, Armenia; 179 Centre de Calcul de l’Institut National de Physique Nucléaire et de Physique des Particules (IN2P3), Villeurbanne, France; 180 CERN, 1211 Geneva 23, Switzerland

## Abstract

A measurement of charged-particle distributions sensitive to the properties of the underlying event is presented for an inclusive sample of events containing a $Z$-boson, decaying to an electron or muon pair. The measurement is based on data collected using the ATLAS detector at the LHC in proton–proton collisions at a centre-of-mass energy of $7$ TeV with an integrated luminosity of $4.6$ fb$^{-1}$. Distributions of the charged particle multiplicity and of the charged particle transverse momentum are measured in regions of azimuthal angle defined with respect to the $Z$-boson direction. The measured distributions are compared to similar distributions measured in jet events, and to the predictions of various Monte Carlo generators implementing different underlying event models.

## Introduction

In order to perform precise Standard Model measurements or to search for new physics phenomena at hadron colliders, it is important to have a good understanding of not only the short-distance *hard* scattering process, but also of the accompanying activity – collectively termed the *underlying event* (UE). This includes partons not participating in the hard-scattering process (beam remnants), and additional hard scatters in the same proton–proton collision, termed multiple parton interactions (MPI). Initial and final state gluon radiation (ISR, FSR) also contribute to the UE activity. It is impossible to unambiguously separate the UE from the hard scattering process on an event-by-event basis. However, distributions can be measured that are sensitive to the properties of the UE.

The soft interactions contributing to the UE cannot be calculated reliably using perturbative quantum chromodynamics (pQCD) methods, and are generally described using different phenomenological models, usually implemented in Monte Carlo (MC) event generators. These models contain many parameters whose values and energy dependences are not known a priori. Therefore, the model parameters must be tuned to experimental data to obtain insight into the nature of soft QCD processes and to optimise the description of UE contributions for studies of hard-process physics.

Measurements of distributions sensitive to the properties of the UE have been performed in proton–proton ($pp$) collisions at $\sqrt{s}=900\;\text {GeV} \,$ and $7\;\text {TeV}$  in ATLAS [1–5], ALICE [6] and CMS [7, 8]. They have also been performed in $p\bar{p}$ collisions in events with jets and in Drell–Yan events at CDF  [9, 10] at centre-of-mass energies of $\sqrt{s}=1.8\;\text {TeV} \,$ and $1.96\;\text {TeV}$ .

This paper reports a measurement of distributions sensitive to the UE, performed with the ATLAS detector [11] at the LHC in $pp$ collisions at a centre-of-mass energy of $7$ TeV. The full dataset acquired during 2011 is used, corresponding to an integrated luminosity of $4.64 \pm 0.08\;{\rm fb}^-1$. Events with a $Z$-boson candidate decaying into an electron or muon pair were selected, and observables constructed from the final state charged particles (after excluding the lepton pair) were studied as a function of the transverse momentum^1^ of the $Z$-boson candidate, $p_\mathrm {{T}}^\mathrm {{Z}}$.

**Fig. 1:**
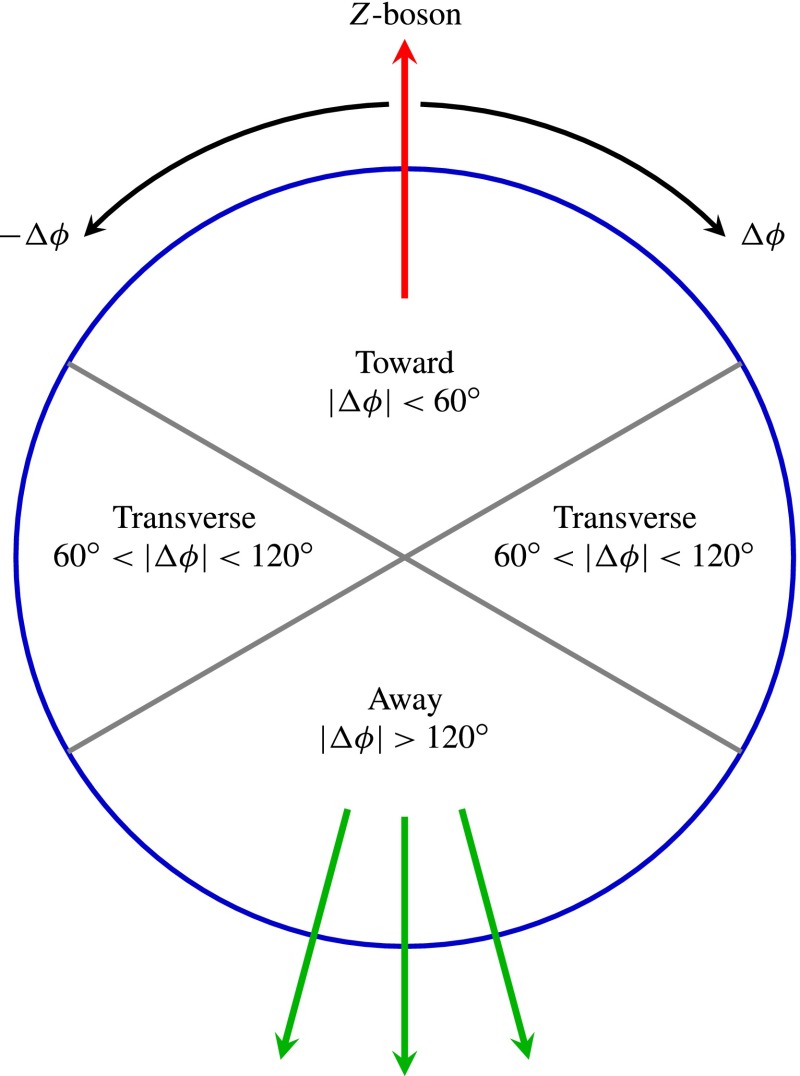
Definition of UE regions as a function of the azimuthal angle with respect to the $Z$-boson

This paper is organised as follows: the definitions of the underlying event observables are given in Sect. 2. The ATLAS detector is described in Sect. 3. In Sect. 4, the MC models used in this analysis are discussed. Sections 5 and 6  describe the event selection, and the correction for the effect of multiple proton–proton interactions in the same bunch crossing (termed pile-up). The correction of the data to the particle level, and the combination of the electron and muon channel results are described in Sect. 7. Section 8 contains the estimation of the systematic uncertainties. The results are discussed in Sect. 9 and finally the conclusions are presented in Sect. 10.

## Underlying event observables

Since there is no final-state gluon radiation associated with a $Z$-boson, lepton-pair production consistent with $Z$-boson decays provides a cleaner final-state environment than jet production for measuring the characteristics of the underlying event in certain regions of phase space. The direction of the $Z$-boson candidate is used to define regions in the azimuthal plane that have different sensitivity to the UE, a concept first used in [12]. As illustrated in Fig. 1, the azimuthal angular difference between charged tracks and the $Z$-boson, $|\Delta \phi |=|\phi -\phi _\text {Z-boson}|$, is used to define the following three azimuthal UE regions:

- $|\Delta \phi | < 60^{\circ }$, the *toward* region,
- $60^{\circ } < |\Delta \phi | < 120^{\circ }$, the *transverse* region, and
- $|\Delta \phi | > 120^{\circ }$, the *away* region.

These regions are well defined only when the measured $p_\mathrm {{T}}^\mathrm {{Z}}$ is large enough that, taking into account detector resolution, it can be used to define a direction. The away region is dominated by particles balancing the momentum of the $Z$-boson except at low values of $p_\mathrm {{T}}^\mathrm {{Z}}$. The transverse region is sensitive to the underlying event, since it is by construction perpendicular to the direction of the $Z$-boson and hence it is expected to have a lower level of activity from the hard scattering process compared to the away region. The two opposite transverse regions may be distinguished on an event-by-event basis through their amount of activity, as measured by the sum of the charged-particle transverse momenta in each of them. The more or less-active transverse regions are then referred to as *trans-max* and* trans-min*, respectively, with the difference between them on an event-by-event basis for a given observable defined as *trans-diff* [13, 14]. The activity in the toward region, which is similarly unaffected by additional activity from the hard scatter, is measured in this analysis, in contrast to the underlying event analysis in dijet events [5].

The observables measured in this analysis are derived from the number, $N_\text {ch}$, and transverse momenta, $p_\mathrm {T}$, of stable charged particles in each event. They have been studied both as one-dimensional distributions, inclusive in the properties of the hard process, and as *profile* histograms which present the dependence of the mean value of each observable (and its uncertainty) on $p_\mathrm {{T}}^\mathrm {{Z}}$. The observables are summarised in Table 1. The mean charged-particle transverse momentum is constructed on an event-by-event basis and is then averaged over all events to calculate the observable mean $p_\mathrm {T}$.

**Table 1:** Definition of the measured observables

Observable	Definition
$p_\mathrm {{T}}^\mathrm {{Z}}$	Transverse momentum of the $Z$-boson
$N_\text {ch}/\delta \eta \,\delta \phi$	Number of stable charged particles per unit $\eta$–$\phi$
$\sum \!p_\mathrm {T}/\delta \eta \,\delta \phi$	Scalar $p_\mathrm {T}$ sum of stable charged particles per unit $\eta$–$\phi$
Mean $p_\mathrm {T}$	Average $p_\mathrm {T}$ of stable charged particles

These are defined for each azimuthal region under consideration except for $p_\mathrm {{T}}^\mathrm {{Z}}$

## The ATLAS detector

The ATLAS detector [11] covers almost the full solid angle around the collision point. The components that are relevant for this analysis are the tracking detectors, the liquid-argon (LAr) electromagnetic sampling calorimeters and the muon spectrometer.

The inner tracking detector (ID) has full coverage in azimuthal angle $\phi$ and covers the pseudorapidity range $|\eta | ~ < 2.5$. It consists of a silicon pixel detector (pixel), a semiconductor tracker (SCT) and a straw-tube transition radiation tracker (TRT). These detectors are located at a radial distance from the beam line of 50.5–150, 299–$560\;a$ nd 563–$1{,}066\;\text {mm}$ , respectively, and are contained within a 2 T axial magnetic field. The inner detector barrel (end-cap) consists of 3 ($2 \times 3$) pixel layers, 4 ($2 \times 9$) layers of double-sided silicon strip modules, and 73 ($2 \times 160$) layers of TRT straw-tubes. These detectors have position resolutions typically of 10, $17\;a$ nd $130\;$ $\,\upmu$m for the $r$–$\phi$ coordinates (only for TRT barrel), respectively. The pixel and SCT detectors provide measurements of the $r$–$z$ coordinates with typical resolutions of $115\;a$ nd $580\;\,$ $\upmu$m, respectively. The TRT acceptance is $|\eta | < 2.0$. A track traversing the barrel typically has 11 silicon hits (3 pixel clusters and 8 strip clusters) and more than 30 straw-tube hits.

A high-granularity lead, liquid-argon electromagnetic sampling calorimeter [15] covers the pseudorapidity range $|\eta | < 3.2$. Hadronic calorimetry in the range $|\eta | < 1.7$ is provided by an iron scintillator-tile calorimeter, consisting of a central barrel and two smaller extended barrel cylinders, one on either side of the central barrel. In the end-caps ($|\eta | > 1.5$), the acceptance of the LAr hadronic calorimeters matches the outer $|\eta |$ limits of the end-cap electromagnetic calorimeters. The LAr forward calorimeters provide both electromagnetic and hadronic energy measurements, and extend the coverage to $|\eta | < 4.9$.

The muon spectrometer (MS) measures the deflection of muon tracks in the large superconducting air-core toroid magnets in the pseudorapidity range $|\eta | < 2.7$. It is instrumented with separate trigger and high-precision tracking chambers. Over most of the $\eta$-range, a precision measurement of the track coordinates in the principal bending direction of the magnetic field is provided by monitored drift tubes. At large pseudorapidities, cathode strip chambers with higher granularity are used in the innermost plane over the range $2.0 < |\eta | < 2.7$.

**Table 2:** Main features of the Monte-Carlo models used. The abbreviations ME, PS, MPI, LO and NLO respectively stand for matrix element, parton shower, multiple parton interactions, leading order and next to leading order in QCD

Generator	Type	Version	PDF	Tune
Pythia 6	LO PS	6.425	CTEQ6L1 [29]	Perugia2011C [30]
Pythia 8	LO PS	8.165	CTEQ6L1	AU2 [31]
Herwig++	LO PS	2.5.1	MRST LO$${**}$$ [32]	UE-EE-3 [33]
Sherpa	LO multi-leg	1.4.0	CT10 [34]	Default
	ME + PS	/1.3.1		
Alpgen	LO multi-leg ME	2.14	CTEQ6L1	
+ Herwig	+ PS	6.520	MRST$${**}$$	AUET2 [35]
+Jimmy	(adds MPI)	4.31		
Powheg	NLO ME	–	CT10	
+ Pythia 8	+ PS	8.165	CT10	AU2

The ATLAS trigger system consists of a hardware-based Level-1 (L1) trigger and a software-based High Level Trigger, subdivided into the Level-2 (L2) and Event-Filter (EF) [16] stages. In L1, electrons are selected by requiring adjacent electromagnetic (EM) trigger towers exceed a certain $E_\mathrm {T}$ threshold, depending on the detector $\eta$. The EF uses the offline reconstruction and identification algorithms to apply the final electron selection in the trigger. The $Z \rightarrow e^+ e^-$ events are selected in this analysis by using a dielectron trigger in the region $|\eta | < 2.5$ with an electron transverse energy, $E_\mathrm {T}$, threshold of $12$ GeV. The muon trigger system, which covers the pseudorapidity range $|\eta | < 2.4$, consists of resistive plate chambers in the barrel ($|\eta | < 1.05$) and thin gap chambers in the end cap regions ($1.05 < |\eta | < 2.4$). Muons are reconstructed in the EF combining L1 and L2 information. The $Z \rightarrow \mu ^+ \mu ^-$ events in this analysis are selected with a first-level trigger that requires the presence of a muon candidate reconstructed in the muon spectrometer with transverse momentum of at least $18$ GeV. The trigger efficiency for the events selected as described in Sect. 5 is very close to $100\,\%$.

## Monte Carlo simulations

Monte Carlo event samples including a simulation of the ATLAS detector response are used to correct the measurements for detector effects, and to estimate systematic uncertainties. In addition, predictions of different phenomenological models implemented in the MC generators are compared to the data corrected to the particle level. Samples of inclusive $Z \rightarrow e^+ e^-$ and $Z \rightarrow \mu ^+ \mu ^-$ events were produced using the leading order (LO) Pythia 6 [17], Pythia 8  [18], Herwig++  [19, 20], Sherpa  [21], Alpgen  [22] and next to leading order (NLO) Powheg  [23] event generators, including various parton density function (PDF) parametrisations. The Alpgen and Sherpa matrix elements are generated for up to five additional partons, thereby filling the phase space with sufficient statistics for the full set of measured observables. It should be noted, that since the measurements are all reported in bins of $p_\mathrm {{T}}^\mathrm {{Z}}$, the results presented in this paper are not sensitive to the predicted shape of the $p_\mathrm {{T}}^\mathrm {{Z}}$ spectrum, even though they are sensitive to jet activity in the event. Table 2 lists the different MC models used in this paper.

Pythia 6, Pythia 8 and Herwig++ are all leading-logarithmic parton shower (PS) models matched to leading-order matrix element (ME) calculations, but with different ordering algorithms for parton showering, and different hadronization models. In scattering processes modelled by lowest-order perturbative QCD two-to-two parton scatters, with a sufficiently low $p_\mathrm {T}$ threshold, the partonic jet cross-section exceeds that of the total hadronic cross-section. This can be interpreted in terms of MPI. In this picture, the ratio of the partonic jet cross-section to the total cross-section is interpreted as the mean number of parton interactions per event. This is implemented using phenomenological models [24], which include (non-exhaustively) further low-$p_\mathrm {T}$ screening of the partonic differential cross-section, and use of phenomenological transverse matter-density profiles inside the hadrons. The connection of colour lines between partons, and the rearrangement of the colour structure of an event by reconnection of the colour strings, are implemented in different ways in these phenomenological models.

The Pythia 6 and Pythia 8 generators both use $p_\mathrm {T}$-ordered parton showers, and a hadronisation model based on the fragmentation of colour strings. The Pythia 8 generator adds to the Pythia 6 MPI model by interleaving not only the ISR emission sequence with the MPI scatters, but also the FSR emissions. The Herwig++   generator implements a cluster hadronization scheme with parton showering ordered by emission angle. The Sherpa generator uses LO matrix elements with a model for MPI similar to that of Pythia 6 and a cluster hadronisation model similar to that of Herwig++. In Alpgen the showering is performed with the Herwig generator. The original Fortran Herwig  [25] generator does not simulate multiple partonic interactions; these are added by the Jimmy  [26] package. The Alpgen generator provides leading-order multi-leg matrix element events: it includes more complex hard process topologies than those used by the other generators, but does not include loop-diagram contributions. The Alpgen partonic events are showered and hadronised by the Herwig+Jimmygenerator combination, making use of MLM matching [22] between the matrix element and parton shower to avoid double-counting of jet production mechanisms. A related matching process is used to interface Pythia 6 to the next-to-leading-order (NLO) Powheg generator, where the matching scheme avoids both double-counting and NLO subtraction singularities [27, 28].

Different settings of model parameters, tuned to reproduce existing experimental data, have been used for the MC generators. The Pythia 6, Pythia 8, Herwig + Jimmy, Herwig++ and Sherpa tunes have been performed using mostly Tevatron and early LHC data. The parton shower generators used with Alpgen and Powheg do not use optimised tunes specific to their respective parton shower matching schemes.

For the purpose of correcting the data for detector effects, samples generated with Sherpa (with the CTEQ6L1 PDF and the corresponding UE tune), and Pythia 8 tune 4C [36] were passed through ATLFAST2 [37], a fast detector simulation software package, which used full simulation in the ID and MS and a fast simulation of the calorimeters. Comparisons between MC events at the reconstructed and particle level are then used to correct the data for detector effects. Since the effect of multiple proton–proton interactions is corrected using a data-driven technique (as described in Sect. 6), only single proton–proton interactions are simulated in these MC samples.

## Event selection

The event sample was collected during stable beam conditions, with all detector subsystems operational. To reject contributions from cosmic-ray muons and other non-collision backgrounds, events are required to have a primary vertex (PV). The PV is defined as the reconstructed vertex in the event with the highest $\sum p_\mathrm {T} ^2$ of the associated tracks, consistent with the beam-spot position (spatial region inside the detector where collisions take place) and with at least two associated tracks with $p_\mathrm {T} > 400$ MeV.

Electrons are reconstructed from energy deposits measured in the EM calorimeter and associated to ID tracks. They are required to satisfy $p_\mathrm {T} > 20$ GeV and $|\eta | < 2.4$, excluding the transition region $1.37 < |\eta | < 1.52$ between the barrel and end-cap electromagnetic calorimeter sections. Electron identification uses shower shape, track-cluster association and TRT criteria [38]. Muons are reconstructed from track segments in the MS associated to ID tracks [39]. They are required to have $p_\mathrm {T} > 20$ GeV and $|\eta | < 2.4$. Both electrons and muons are required to have longitudinal impact parameter multiplied by $\sin \theta$ of the ID track, $|z_0|\sin \theta < 10$ mm with respect to the PV. The dilepton invariant mass of oppositely charged leptons, $m_{\mathrm {ll}}$, is required to be in the region $66 < m_{\mathrm {ll}}< 116$ GeV at this stage. No explicit isolation requirement is applied to the muons, but in the case of electrons, some isolation is implied by the identification algorithm. The correction for this effect is discussed in Sect. 7.3.

The tracks in the calculation of UE observables satisfy the following criteria [40]:

- $$p_\mathrm {T} > 0.5$$ GeV and $$|\eta | < 2.5$$;
- a minimum of one pixel and six SCT hits;
- a hit in the innermost pixel layer, if the corresponding pixel module was active;
- transverse and longitudinal impact parameters with respect to the PV, $|d_0| < 1.5$ mm and $|z_0|\sin \theta < 1.5$ mm, respectively;
- for tracks with $p_\mathrm {T} > 10$ GeV, a goodness of fit probability greater than $0.01$ in order to remove mis-measured tracks.

The tracks corresponding to the leptons forming the $Z$-boson candidate are excluded.

## Correction for pile-up

The average expected number of pile-up events per hard-scattering interaction ($\mu$) was typically in the range $3{-}12$ in the 2011 dataset. Of the tracks selected by the procedure described above and compatible with the PV of the hard-scattering event, up to $15\,\%$ originate from pile-up, as described below. Due to the difficulty in modelling accurately the soft interactions in $pp$ collisions and the fact that pile-up conditions vary significantly over the data-taking period, a data-driven procedure has been derived to correct the measured observables for the pile-up contribution.

The measured distribution of any track-based observable can be expressed as the convolution of the distribution of this variable for the tracks originating from the $Z$-boson production vertex, with the distribution resulting from the superimposed pile-up interactions. The pile-up contribution is estimated from data by sampling tracks originating from a vertex well separated from the hard-scattering PV. In each event, the pile-up contribution to a given observable is derived from tracks selected with the same longitudinal and transverse impact parameter requirements as the PV tracks, but with respect to two points located at $z$ distances of $+2$ cm and $-2$ cm from the hard-scattering PV. The shift of $2$ cm relative to the PV introduces a bias in the density of the pile-up interactions. This is corrected on the basis of the shape of the distribution of the $z$ distance between pairs of interactions in the same bunch crossing. This distribution is well approximated by a Gaussian with variance $\sigma = \sqrt{2}\sigma _{BS}$, where $\sigma _{BS} \approx 6$ cm is the effective longitudinal variance of the interaction region averaged over all events. Pile-up distributions are thus obtained for each observable and are deconvoluted from the corresponding measured distributions at the hard-scattering PV.

**Fig. 2:**
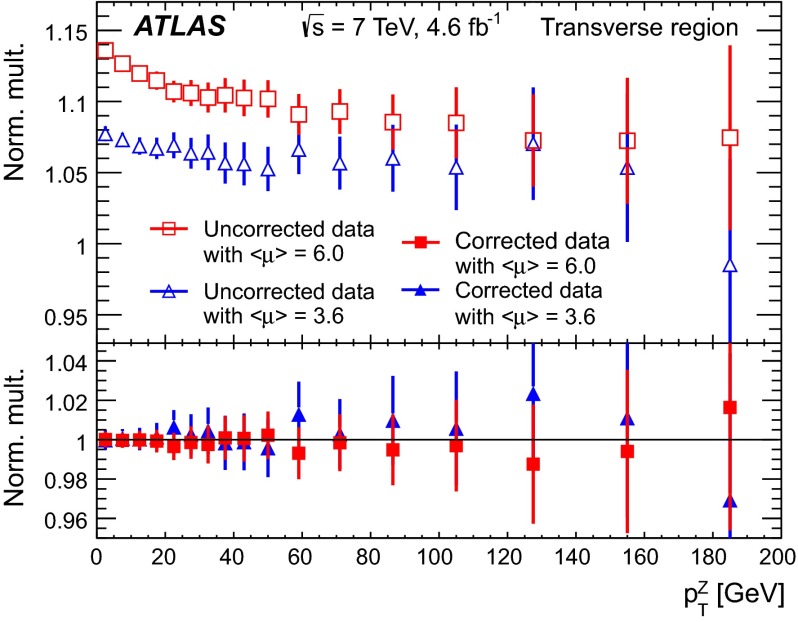
Average charged particle multiplicity density, $\langle N_\text {ch}/\delta \eta \,\delta \phi \rangle$ in the transverse region for two samples with different average numbers of interactions, $\langle \mu \rangle$, normalised to the average density in the full sample after pile-up correction, before (*top*) and after (*bottom*) pile-up correction. The data are shown as a function of the transverse momentum of the $Z$-boson, $p_\mathrm {{T}}^\mathrm {{Z}}$. Only statistical uncertainties are shown

The stability of the pile-up correction for different beam conditions is demonstrated in Fig. 2. The figure compares the distributions of the average charged particle multiplicity density, $\langle N_\text {ch}/\delta \eta \,\delta \phi \rangle$ as a function of $p_\mathrm {{T}}^\mathrm {{Z}}$, before and after pile-up correction, for two sub-samples with an average of $3.6$ and $6$ interactions per bunch crossing ($\langle \mu \rangle$), respectively. Each distribution is normalised to that obtained for the full sample after pile-up correction. The dependence of the normalised charged multiplicity distributions on $p_\mathrm {{T}}^\mathrm {{Z}}$ which can be seen before correction in Fig. 2 reflects the fact that actual contributions to this observable depend on $p_\mathrm {{T}}^\mathrm {{Z}}$, while the pile-up contribution is independent of $p_\mathrm {{T}}^\mathrm {{Z}}$. The pile-up corrected results agree to better than $2\,\%$, a value much smaller than the size of the correction, which may be as large as $20\,\%$ for this observable in low $p_\mathrm {{T}}^\mathrm {{Z}}$ bins for the data-taking periods with the highest values of $\langle \mu \rangle$. The systematic uncertainty arising from this procedure is discussed in Sect. 8.

## Unfolding to particle level, background corrections and channel combination

After correcting for pile-up, an iterative Bayesian unfolding [41] of all the measured observables to the particle level is performed. This is followed by a correction of the unfolded distributions for the small amount of background from other physics processes. At this point, the electron and muon measurements are combined to produce the final results.

### Unfolding

The measurements are presented in the fiducial region defined by the $Z$-boson reconstructed from a pair of oppositely charged electrons or muons each with $p_\mathrm {T} > 20$ GeV and $|\eta | < 2.4$ and with a lepton pair invariant mass in the range $66 < m_{\mathrm {ll}}< 116$ GeV.

The results in Sect. 9 are presented in the Born approximation, using the leptons before QED FSR to reconstruct the $Z$-boson. These results are also provided in HEPDATA [42] using *dressed* leptons. These are defined by adding vectorially to the $4$-momentum of each lepton after QED FSR the $4$-momenta of any photons not produced in hadronic decays and found within a cone of $\Delta R = 0.1$ around the lepton, where the angular separation $\Delta R$ is given by $\sqrt{(\Delta \eta )^2 + (\Delta \phi )^2}$.

The UE observables are constructed from stable charged particles with $p_\mathrm {T} > 0.5$ GeV and $|\eta | < 2.5$, excluding $Z$-boson decay products. Stable charged particles are defined as those with a proper lifetime $\tau > 0.3 \times 10^{-10}\;{\!\!}$s, either directly produced in $pp$ interactions or from the subsequent decay of particles with a shorter lifetime.

Bayesian iterative unfolding was used to correct for residual detector resolution effects. This method requires two inputs: an input distribution of the observable (the MC generator-level distribution is used for this), and a detector response matrix which relates the uncorrected measured distribution in this observable to that defined at the event generator level, also termed the particle level. The detector response matrix element, $S_{ij}$ is the probability that a particular event from bin $i$ of the particle-level distribution is found in bin $j$ of the corresponding reconstructed distribution, and is obtained using simulation. For the profile histogram observables in this paper, a two-dimensional (2D) histogram was created with a fine binning for the observable of interest, such that each unfolding bin corresponds to a region in the 2D space.

The unfolding process is iterated to avoid dependence on the input distribution: the corrected data distribution produced in each iteration is used as the input for the next. In this analysis, four iterations were performed since this resulted only in a small residual bias when tested on MC samples while keeping the statistical uncertainties small. The unfolding uses the Sherpa simulation for the input distributions and unfolding matrix. In the muon channel, the MC events are reweighted at the particle level in terms of a multi-variable distribution constructed for each distribution of interest using the ratio of data to detector-level MC, so that the detector-level MC closely matches the data. This additional step is omitted in the electron channel for the reasons discussed in Sect. 7.3.

The dominant correction to the data is that related to track reconstruction and selection efficiencies, in particular at low-$p_\mathrm {T}$. After the selection described in Sect. 5, the rate of fake tracks (those constructed from tracker noise and/or hits which were not produced by a single particle) is found to be very small. This, as well as a small contribution of secondaries (i.e. tracks arising from hadronic interactions, photon conversions to electron–positron pairs, and decays of long-lived particles) is corrected for by the unfolding procedure.

### Backgrounds

The background to the $Z$-boson signal decaying into a lepton pair consists of a dominant component from multijet production, smaller components from other physics sources, and a very small component from non-collision backgrounds. A fully data-driven correction procedure has been developed and applied directly to the unfolded distributions to take into account the influence of the backgrounds.

The primary vertex requirement removes almost all of the beam-induced non-collision background events. Similarly, the impact parameter requirements on the leptons reduce the cosmic-ray background to a level below $0.1\,\%$ of the signal. These residual backgrounds were considered as negligible in the analysis.

The $pp$ collision backgrounds to $Z \rightarrow e^+ e^-$ or $Z \rightarrow \mu ^+ \mu ^-$ decays were found to be of the order of a few percent of the signal in the mass window [43]. The *resonant* backgrounds from $WZ$, $ZZ$ and $Z\gamma$ pair production with a $Z$ boson decaying into leptons were estimated from simulated samples and found to amount to less than 0.2 % of the selected events. Their impact on the underlying event observables is negligible and they were not considered further here.

The contribution from the *non-resonant* backgrounds (*i.e.* from all other $pp$ collision processes) is larger, typically between $1$ and $2\,\%$ of the signal, depending on the $p_\mathrm {{T}}^\mathrm {{Z}}$ range considered, and is dominated by multijet production with a combination of light-flavour jets misidentified as electrons and heavy-flavour jets with a subsequent semileptonic decay of a charm or beauty hadron. This contribution is estimated to correspond to 0.5 % of the signal for $Z \rightarrow e^+ e^-$ decays and to 1–2 $\%$ of the signal for $Z \rightarrow \mu ^+ \mu ^-$ decays. The background in the electron channel is somewhat lower because of the implicit isolation requirement imposed on the electrons through the electron identification requirements. Smaller contributions to the non-resonant background arise from diboson, $t\bar{t}$ and single top production and amount to less than 0.3 % of the signal, increasing to $1\,\%$ at $p_\mathrm {{T}}^\mathrm {{Z}} > 50$ GeV. The still smaller contributions from processes such as $W$ or $Z$ production with jets, where a jet is misidentified as a lepton, are treated in the same way as the multijet background. These contributions amount to less than $0.1\,\%$ of the signal sample.

**Fig. 3:**
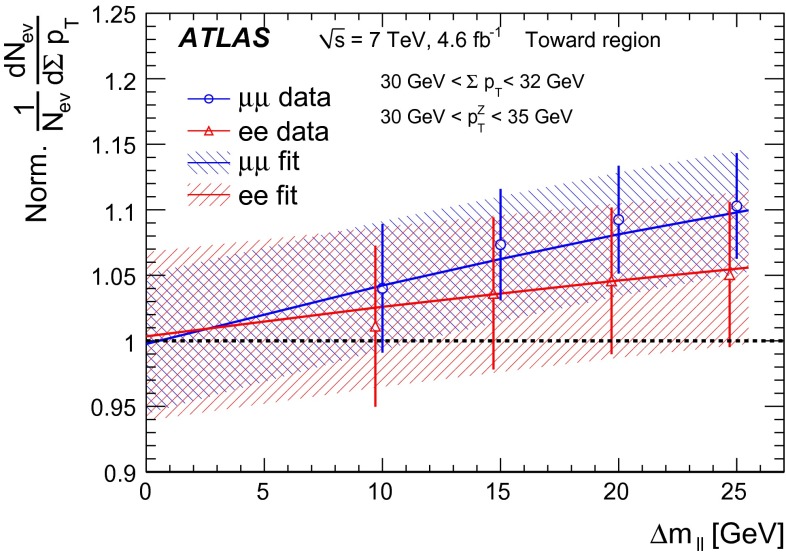
Impact of non-resonant backgrounds on the measurement of $\sum p_\mathrm {T}$ in the bin $30$ GeV $< \Sigma p_\mathrm {T} < 32$ GeV and in the toward region for $30$ GeV $< p_\mathrm {{T}}^\mathrm {{Z}} < 35$ GeV. This is shown separately for the electron and muon channels as a function of the window applied to the dilepton mass $|m_{\mathrm {ll}}- M_{\mathrm {Z}}| < \Delta m_{\mathrm {ll}}$. The unfolded value for each channel is normalised to the corrected combined result. The statistical uncertainties at individual $\Delta m_{\mathrm {ll}}$ points are strongly correlated within each channel. The uncertainty range of the linear fit is shown by hatched bands for each channel. This includes the statistical and systematic uncertainties from the fit itself, as well as the relevant correlations. The *vertical line* at $\Delta m_{\ell \ell } = 0$ marks the points to which the extrapolations are made

The non-resonant background is corrected for by studying the UE observables as a function of $\Delta m_{\mathrm {ll}}$, the half-width of the mass window around the $Z$-boson signal peak. Since the distributions of UE observables in non-resonant background processes are found to be approximately constant as a function of the dilepton mass and the background shape under the $Z$-boson mass peak is approximately linear, the background contribution to any UE observable is approximately proportional to $\Delta m_{\mathrm {ll}}$. Thus, the background contribution can be corrected for by calculating the UE observables for different values of $\Delta m_{\mathrm {ll}}$, chosen here to be between $10$ and $25$ GeV, and extracting the results which could be measured for a pure signal with $\Delta m_{\mathrm {ll}}\rightarrow 0$. This procedure is performed separately for each bin of the distributions of interest.

The validity of the linear approximation for the $\Delta m_{\mathrm {ll}}$ dependence of the background contribution was checked for all observables studied in this analysis. An example is presented in Fig. 3, where the $\Delta m_{\mathrm {ll}}$ dependence is shown for one bin of the $\sum p_\mathrm {T}$ differential distribution, as obtained in the toward region for $30 < p_\mathrm {{T}}^\mathrm {{Z}} < 35$ GeV and shown separately for the electron and muon channels. The values plotted in Fig. 3 are normalised to the corrected combined value. The values of the observables in the muon channel increase linearly with $\Delta m_{\mathrm {ll}}$. The difference in the slope observed between the muon and the electron samples is due to the larger background in the muon channel, as discussed above. A straight line is fitted through the points obtained for the various $\Delta m_{\mathrm {ll}}$ values shown in Fig. 3 for each channel. For each bin in the observable and $p_\mathrm {{T}}^\mathrm {{Z}}$ , the muon and electron channels values agree with each other after extrapolating to $\Delta m_{\mathrm {ll}}= 0$ within the uncertainties of the fit procedure, which are represented by the shaded areas and include the statistical and systematic uncertainties from the fit itself (as discussed in Sect. 8, as well as the relevant correlations.

The effect of the background on the unfolded distributions can be summarised as follows: in the case of the electron channel, which has less background than the muons, the background in the average values of $\sum p_\mathrm {T}$ and $N_\text {ch}$ is below $1\,\%$. The absence of any isolation requirement applied to the muons leads to significantly higher background levels in certain regions, with corrections ranging from as high as 6–8 $\%$ for the average values of $\sum p_\mathrm {T}$ in the toward region at high $p_\mathrm {{T}}^\mathrm {{Z}}$, to about $1\,\%$ for the average values of $N_\text {ch}$. The background correction is done after unfolding to avoid resolution issues present at the detector level.

### Combination of the electron and muon channels

Before combining the electron and muon channels, the analysis must correct for a bias over a limited region of the phase space which affects the measurements in the electron channel when one of the electrons is close to a jet produced in association with the $Z$ boson. This bias is observed at high $p_\mathrm {{T}}^\mathrm {{Z}}$, mostly in the toward region and to a lesser extent in the transverse region, and affects the $\sum p_\mathrm {T}$ distribution for high values of $\sum p_\mathrm {T}$, typically $\sum p_\mathrm {T} > 30$ GeV. It arises from the imperfect modelling of the electron shower shape variables in the simulation, which leads to an underestimate of the electron identification efficiency for electrons close to jets. The bias on the observable can be as large as $50\,\%$ for $\sum p_\mathrm {T} = 100$ GeV. Since it is not reproduced precisely enough by the simulation of the electron shower, in the relevant narrow regions of phase space a tightened isolation

**Fig. 4:**
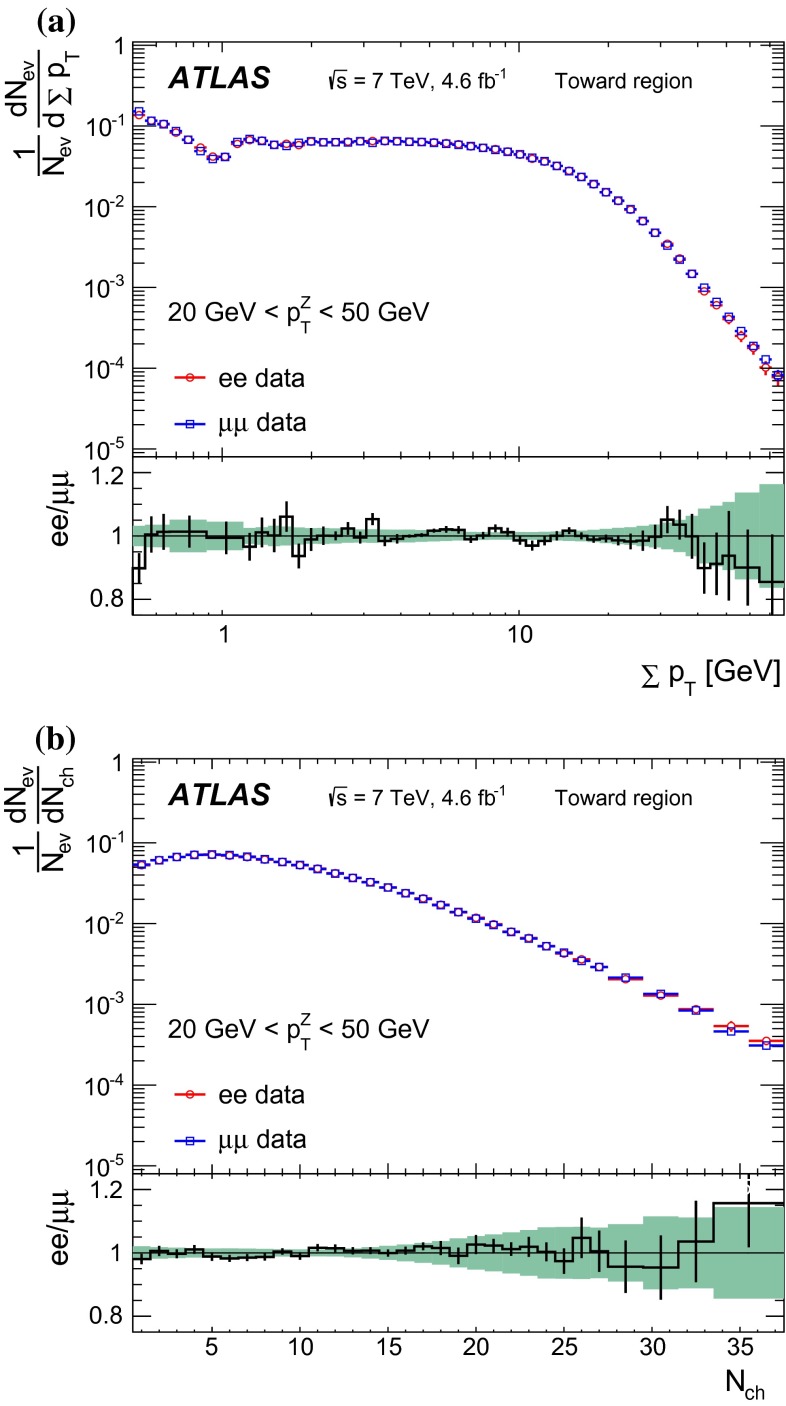
Unfolded and corrected distributions of charged particle $\sum p_\mathrm {T}$ (**a**) and $N_\text {ch}$ (**b**) for $20 < p_\mathrm {{T}}^\mathrm {{Z}} < 50$ GeV shown separately for the $Z \rightarrow e^+ e^-$ and $Z \rightarrow \mu ^+ \mu ^-$ samples after all corrections have been applied. The *bottom panels* show the ratios between the electron and the muon distributions where the *error bars* are purely statistical and the *shaded areas* represent the total uncertainty, including systematic, on the combined result

criterion was applied to electrons to exclude the mismodelled event configurations and the proper geometric correction was deduced from the muon channel unaffected by jet overlap. The combined results for electrons and muons in the affected bins are assigned a larger uncertainty, since the contribution of events from the electron-decay channel is significantly reduced leading to a larger overall uncertainty. The most significant effect is observed for the $\sum p_\mathrm {T}$ $>100$ GeV in the toward and transverse region.

As discussed in Sect. 2 and in Sect. 7.1, the electron and muon results are unfolded and then combined, both as Born-level lepton pairs and as dressed lepton pairs, and accounting for the uncorrelated and correlated terms in the systematic uncertainties between the channels (as described in Sect. 8). Combining the dressed electron and muon pairs induces $<0.1\,\%$ additional systematic uncertainty on the UE observables compared to the Born level results.

Figure 4 illustrates the excellent agreement between the fully unfolded and corrected UE observables for the electron and muon channels, once the specific correction procedure described above has been applied to the electron channel in the limited phase space regions where significant hadronic activity occurs close to one of the electrons. As shown for the specific region $20 < p_\mathrm {{T}}^\mathrm {{Z}} < 50$ GeV in Fig. 4, the differential distributions for $\sum p_\mathrm {T}$ and $N_\text {ch}$ agree within statistical uncertainties over most of the range of relevance, except for high values of $\sum p_\mathrm {T}$, where the electron bias has been corrected as described above, and where the total uncertainty on the combined measurement has been enlarged as shown by the shaded error band in the ratio plot. The shape of the $\sum p_\mathrm {T}$ distribution in the region around $1$ GeV reflects the $p_\mathrm {T}$ threshold of $0.5$ GeV applied in the track selection.

## Systematic uncertainties

The following sources of uncertainty have been assessed for the measured distributions after all corrections and unfolding. Table 3 summarises the typical sizes of the systematic uncertainties for the UE observables as a function of $p_\mathrm {{T}}^\mathrm {{Z}}$.

**Table 3:** Typical contributions to the systematic uncertainties (in %) on the unfolded and corrected distributions of interest in the toward and transverse regions for the profile distributions. The range of values in the columns 3–5 indicate the variations as a function of $p_\mathrm {{T}}^\mathrm {{Z}}$, while those in the last column indicate the variations as a function of $N_\text {ch}$. The column labelled *Correlation* indicates whether the errors are treated as correlated or not between the electron and muon channels

Observable	Correlation	$N_\text {ch}$ vs $p_\mathrm {{T}}^\mathrm {{Z}}$	$\sum p_\mathrm {T}$ vs $p_\mathrm {{T}}^\mathrm {{Z}}$	Mean $p_\mathrm {T}$ vs $p_\mathrm {{T}}^\mathrm {{Z}}$	Mean $p_\mathrm {T}$ vs $N_\text {ch}$
Lepton selection	No	0.5–1.0	0.1–1.0	${<}0.5$	0.1–2.5
Track reconstruction	Yes	1.0–2.0	0.5–2.0	${<}0.5$	${<}0.5$
Impact parameter requirement	Yes	0.5–1.0	1.0–2.0	0.1–2.0	${<}0.5$
Pile-up removal	Yes	0.5–2.0	0.5–2.0	${<}0.2$	0.2–0.5
Background correction	No	0.5–2.0	0.5–2.0	${<}0.5$	${<}0.5$
Unfolding	No	0.5–3.0	0.5–3.0	${<}0.5$	0.2–2.0
Electron isolation	No	0.1–1.0	0.5–2.0	0.1–1.5	${<}1.0$
Combined systematic uncertainty		1.0–3.0	1.0–4.0	${<}1.0$	1.0–3.5

- Lepton selection: systematic uncertainties due to the lepton selection efficiencies have been assessed using MC simulation. The data are first unfolded using the nominal MC samples, then with samples corresponding to a $\pm 1 \sigma$ variation of the efficiencies [43]. These uncertainties are assumed to be uncorrelated between the electron and muon channels. The resulting uncertainty is less than $1\,\%$ for all observables over most of the kinematic range.
- Track reconstruction: the systematic uncertainty on the track reconstruction efficiency originating from uncertainties on the detector material description is estimated as in Ref. [44] for particles with $|\eta | < 2.1$ and as in Ref. [40] for $|\eta | > 2.1$. The typical value for $|\eta | < 2.1$ is $\pm 1\,\%$ while it is approximately $5\,\%$ for $|\eta | > 2.1$. The effect of this uncertainty on the final results is less than $2\,\%$. This uncertainty is fully correlated between the electron and muon channels.
- Impact parameter requirement: the fraction of secondary particles (i.e. those originating from decays and interactions in the inner detector material) in data is reproduced by the MC simulation to an accuracy of $\sim$ 10–20 $\%$, obtained by comparing $d_0$ distributions in MC and in the data corrected for pile-up. To assess the corresponding systematic uncertainty, the track impact parameter requirements on $|d_0|$ and $|z_0|\mathrm {sin}\theta$ are varied from the nominal values of $1.5$ to $1.0$ and $2.5$ mm, resulting in fractions of secondaries varying between $0.5$ to $4.0\,\%$, and the resulting distributions are unfolded using MC samples selected with the same impact parameter requirements. The maximum residual difference of $2\,\%$ or less between these unfolded distributions and the nominal unfolded distribution is taken as the uncertainty arising from this requirement. This uncertainty is also fully correlated between the electron and muon channels.
- Pile-up correction: the pile-up correction uncertainty originates from the uncertainty in the pile-up density fitted along with the spatial distribution of tracks originating from pile-up, and the difference between the pile-up densities measured for $Z$-boson and for randomly triggered events. In addition to these, the stability of the correction method with respect to the instantaneous luminosity was estimated by performing the correction procedure independently on datasets with different average numbers of reconstructed vertices, as shown in Fig. 2. The total uncertainty due to the pile-up correction is taken to be the quadratic combination of the uncertainties from these sources, and it is at most $2\,\%$ for the average underlying event observables. The overall uncertainty is fully correlated between the electron and muon channels.
- Background correction: the uncertainty is evaluated by comparing the results of the linear fit to those obtained using a second-order polynomial. This uncertainty is at most $2\,\%$ for the maximum background uncertainty on $\sum p_\mathrm {T}$, which is the most strongly affected variable, and is assumed to be uncorrelated between the electron and muon channels. Any potential correlation arising from the common $t\overline{t}$ and diboson backgrounds is neglected because they become sizable only for $p_\mathrm {{T}}^\mathrm {{Z}} > 100$ GeV, where the total uncertainty is dominated by the statistical uncertainity on the background.
- Unfolding: the uncertainty due to the model-dependence of the unfolding procedure is taken from the degree of non-closure between the Pythia 8 initial particle-level distributions and the corresponding detector-level Pythia 8 distributions unfolded and corrected using the Sherpa sample, which was reweighted to agree with Pythia 8 at the detector level. This uncertainty varies between $0.5$ and $3\,\%$ for the profile distributions, and is assumed to be uncorrelated between the electron and muon channels.
- Bias due to implicit isolation: this uncertainty is estimated by varying the electron isolation requirement used to derive the correction discussed in Sect. 7.3. The uncertainty is assigned to the electron channel and does not exceed $\sim$1 % for the profile distributions.

**Fig. 5:**
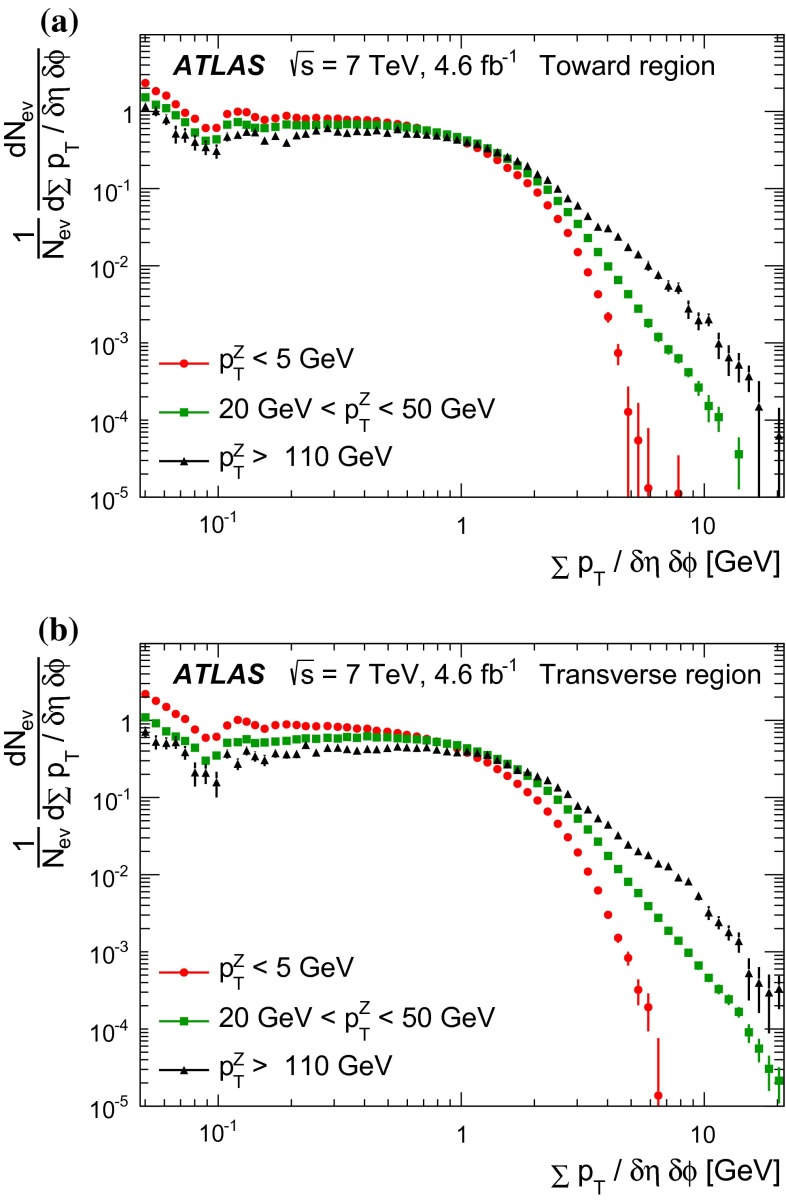
Distributions of the scalar $p_\mathrm {T}$ sum density of charged particles, $\sum \!p_\mathrm {T}/\delta \eta \,\delta \phi$, in three different $Z$-boson transverse momentum, $p_\mathrm {{T}}^\mathrm {{Z}}$, intervals, in the toward (**a**) and transverse (**b**) regions. The *error bars* depict combined statistical and systematic uncertainties

**Fig. 6:**
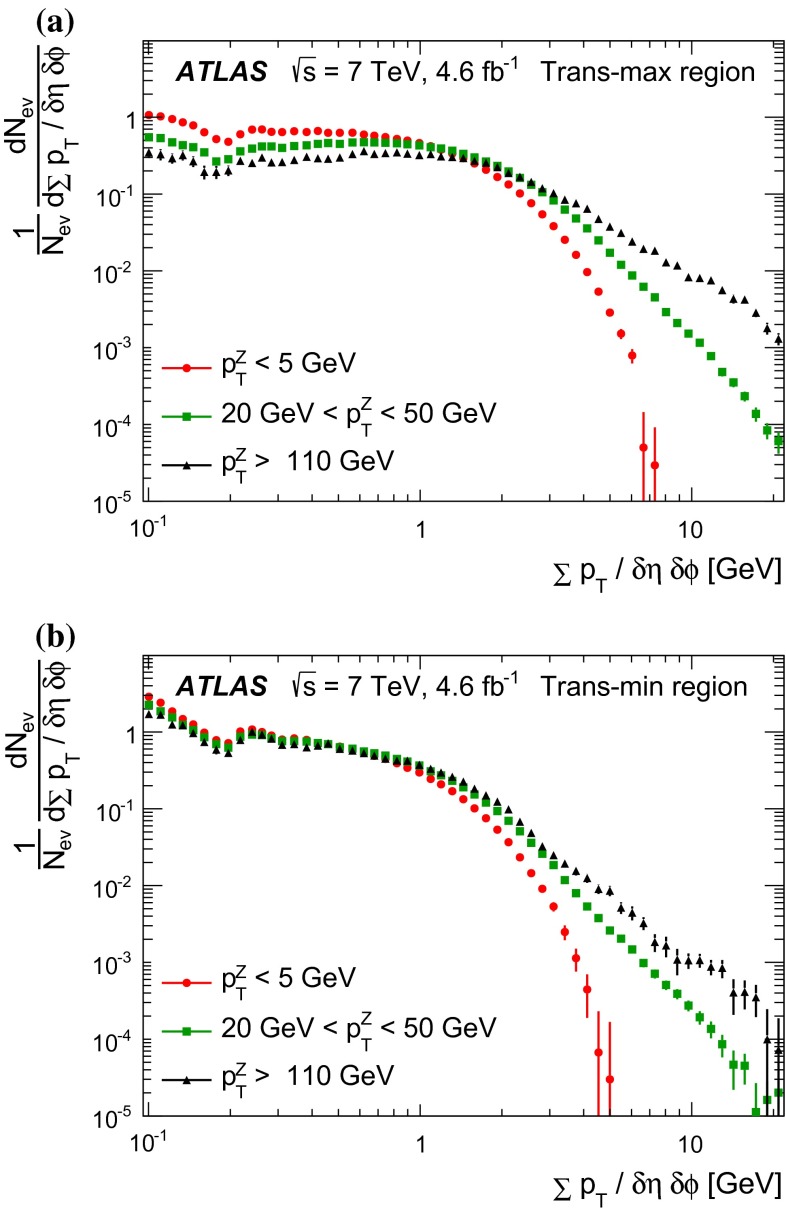
Distributions of the scalar $p_\mathrm {T}$ sum density of charged particles, $\sum \!p_\mathrm {T}/\delta \eta \,\delta \phi$, in three different $Z$-boson transverse momentum, $p_\mathrm {{T}}^\mathrm {{Z}}$, intervals, in the trans-max (**a**) and trans-min (**b**) regions. The *error bars* depict combined statistical and systematic uncertainties

**Fig. 7:**
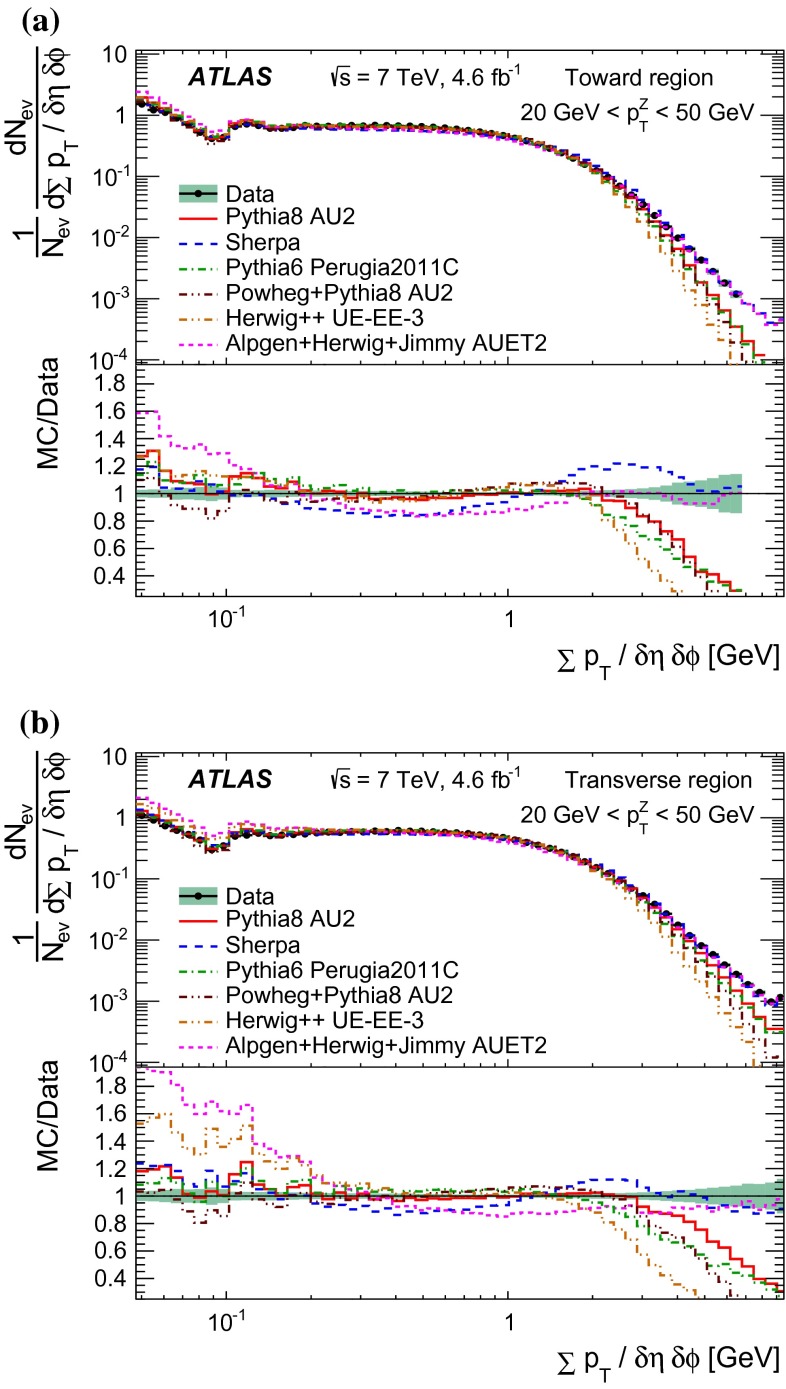
Comparisons of data and MC predictions for the scalar $p_\mathrm {T}$ sum density of charged particles, $\sum \!p_\mathrm {T}/\delta \eta \,\delta \phi$, for $Z$-boson transverse momentum, $p_\mathrm {{T}}^\mathrm {{Z}}$, in the interval 20–50 $\text {GeV}$, in the toward (**a**) and transverse (**b**) regions. The *bottom panels* in each plot show the ratio of MC predictions to data. The *shaded bands* represent the combined statistical and systematic uncertainties, while the *error bars* show the statistical uncertainties

**Fig. 8:**
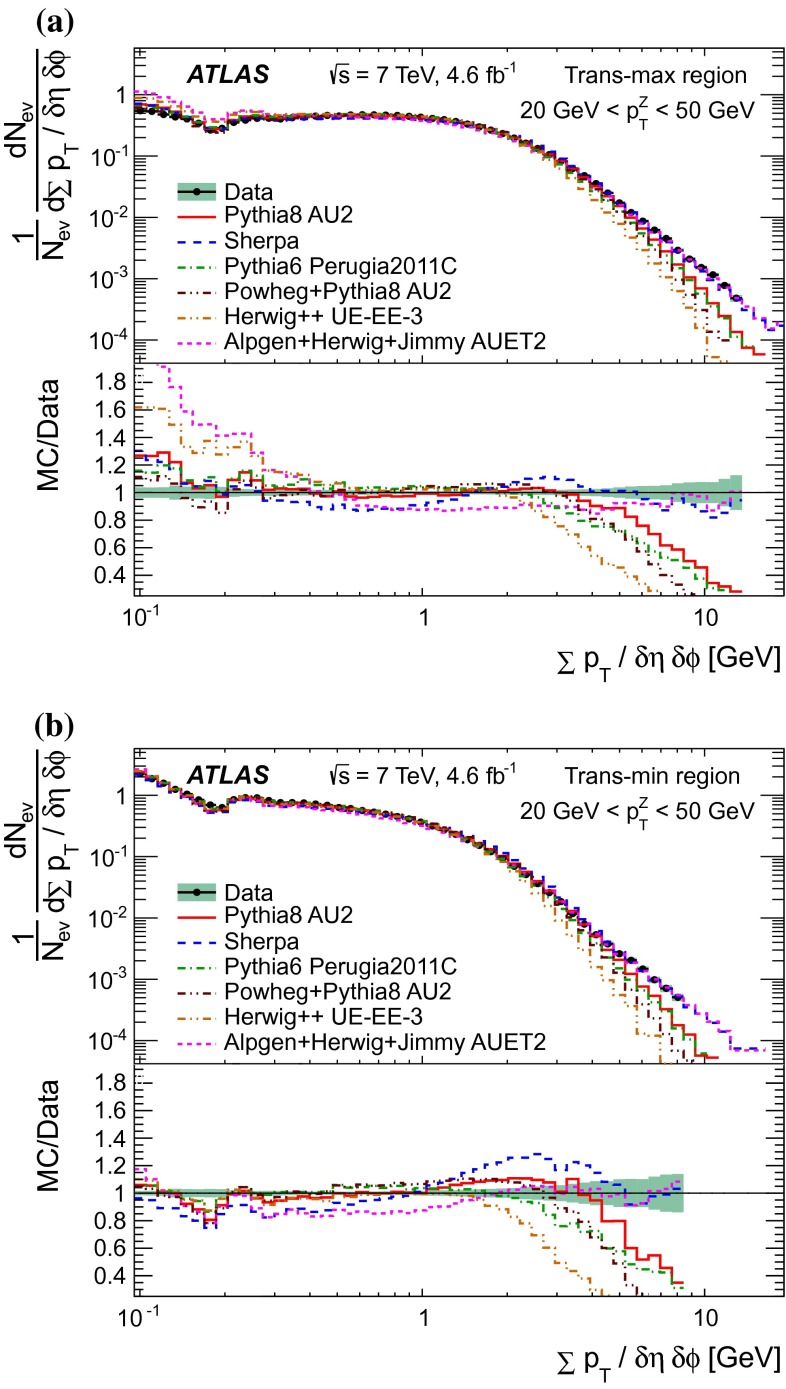
Comparisons of data and MC predictions for the scalar $p_\mathrm {T}$ sum density of charged particles, $\sum \!p_\mathrm {T}/\delta \eta \,\delta \phi$, for $Z$-boson transverse momentum, $p_\mathrm {{T}}^\mathrm {{Z}}$, in the interval 20–50 $\text {GeV}$, in the trans-max (**a**) and trans-min (**b**) regions. The *bottom panels* in each plot show the ratio of MC predictions to data. The *shaded bands* represent the combined statistical and systematic uncertainties, while the *error bars* show the statistical uncertainties

**Fig. 9:**
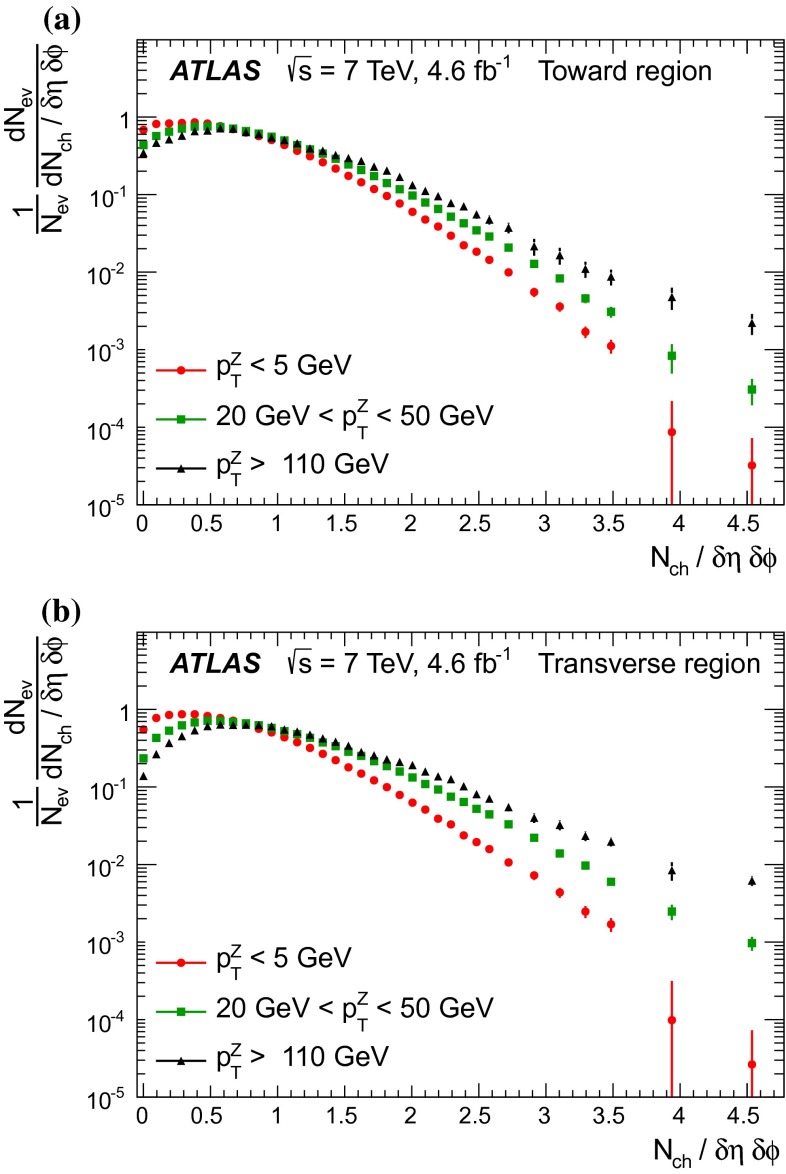
Distributions of charged particle multiplicity density, $N_\text {ch}/\delta \eta \,\delta \phi$ , in three different $Z$-boson transverse momentum, $p_\mathrm {{T}}^\mathrm {{Z}}$, intervals, in the toward (**a**) and transverse (**b**) regions. The *error bars* depict combined statistical and systematic uncertainties

Other potential sources of systematic uncertainty have been found to be negligible. The total uncertainty in each measured bin is obtained by propagating the systematic component of the error matrix through the channel combination. For the differential distributions in Sect. 9.2, the unfolding model dependent uncertainty increases to about $5\,\%$, resulting in slightly larger overall systematic uncertainties.

## Results

### Overview of the results

The results are shown in Sect. 9.2, first for the differential distributions of charged particle $\sum p_\mathrm {T}$ and $N_\text {ch}$ in intervals of $p_\mathrm {{T}}^\mathrm {{Z}}$, and then for the same distributions for a representative $p_\mathrm {{T}}^\mathrm {{Z}}$ range compared to MC model predictions. The normalised quantities, $N_\text {ch}/\delta \eta \,\delta \phi$ and $\sum \!p_\mathrm {T}/\delta \eta \,\delta \phi$, are obtained by dividing $N_\text {ch}$ or $\sum p_\mathrm {T}$ by the angular area in $\eta$–$\phi$ space. This allows for direct comparisons between the total transverse and trans-min/max quantities, and between the current result and experiments with different angular acceptances. The angular areas for the transverse, toward, and away region observables are $\delta \phi \, \delta \eta = (2\times \pi /3) \times (2 \times 2.5) = 10\pi /3$, while for trans-max/min/diff, $\delta \phi \, \delta \eta =5\pi /3$.

Since the away region is dominated by the jets balancing the $p_\mathrm {{T}}^\mathrm {{Z}}$  [43], the focus will be on the toward, transverse, trans-max and trans-min regions. In the transverse region, the extra jet activity is more likely to be assigned to the trans-max region. Assuming the same flat UE activity in trans-min and trans-max regions, the trans-diff region, the difference between the observables measured in trans-max and trans-min regions, is expected to be dominated by the hard scattering component. In  Sect. 9.3 profile histograms are shown. Finally, in Sect. 9.4, the results are compared to previous measurements from ATLAS where distributions sensitive to the underlying event were measured as a function of the kinematics of either the leading charged particle [1], or the leading jet [5].

### Differential distributions

The distributions of the charged-particle $\sum \!p_\mathrm {T}/\delta \eta \,\delta \phi$ and $N_\text {ch}/\delta \eta \,\delta \phi$ in intervals of $p_\mathrm {{T}}^\mathrm {{Z}}$ show the dependence of the event activity on the hard scale. The distributions of $\sum \!p_\mathrm {T}/\delta \eta \,\delta \phi$ in three different $p_\mathrm {{T}}^\mathrm {{Z}}$ ranges are shown in Fig. 5 and in Fig. 6. At values below $\sum \!p_\mathrm {T}/\delta \eta \,\delta \phi$ of $0.1$ GeV, the distributions exhibit a decrease, which is independent of $p_\mathrm {{T}}^\mathrm {{Z}}$. This is followed by a sharp increase at higher $\sum \!p_\mathrm {T}/\delta \eta \,\delta \phi$, which is an artifact of requiring at least two tracks with $p_\mathrm {T}$ of at least $0.5$ GeV in every event. Then a broad distribution can be seen extending to $\sum \!p_\mathrm {T}/\delta \eta \,\delta \phi$ of about $1$ GeV, followed by a steep decrease, the rate of which depends on the $p_\mathrm {{T}}^\mathrm {{Z}}$ interval. For lower $p_\mathrm {{T}}^\mathrm {{Z}}$ values, the decrease is faster. These features are fairly independent of the UE regions, with the exception of the trans-min region, in which the $\sum \!p_\mathrm {T}/\delta \eta \,\delta \phi$ distribution is approximately independent of $p_\mathrm {{T}}^\mathrm {{Z}}$ up to $\sum \!p_\mathrm {T}/\delta \eta \,\delta \phi$ of $1$ GeV. If there were no hard scattering contributions in the trans-min region and the remaining underlying event activity were independent of the hard scattering scale then this $p_\mathrm {{T}}^\mathrm {{Z}}$ independence of the $\sum \!p_\mathrm {T}/\delta \eta \,\delta \phi$ distribution would be expected [45].

**Fig. 10:**
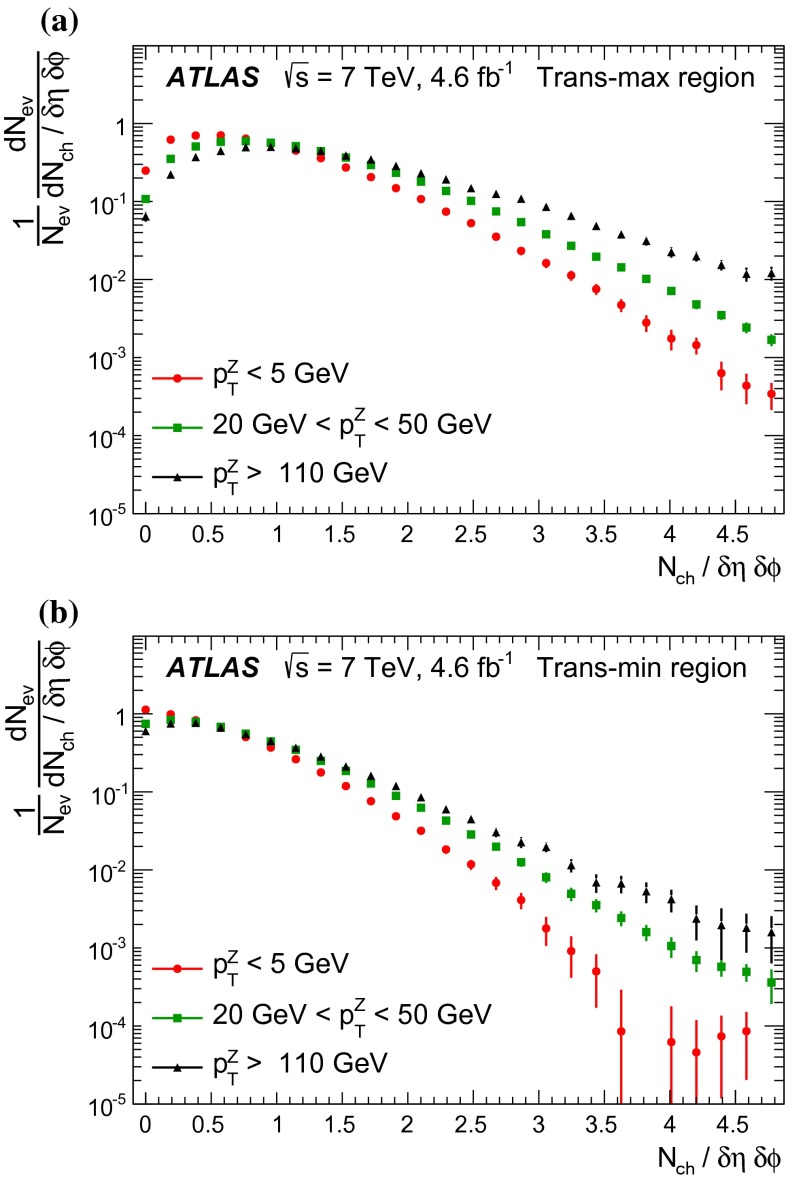
Distributions of charged particle multiplicity density, $N_\text {ch}/\delta \eta \,\delta \phi$ , in three different $Z$-boson transverse momentum, $p_\mathrm {{T}}^\mathrm {{Z}}$, intervals, in the trans-max (**a**) and trans-min (**b**) regions. The *error bars* depict combined statistical and systematic uncertainties

**Fig. 11:**
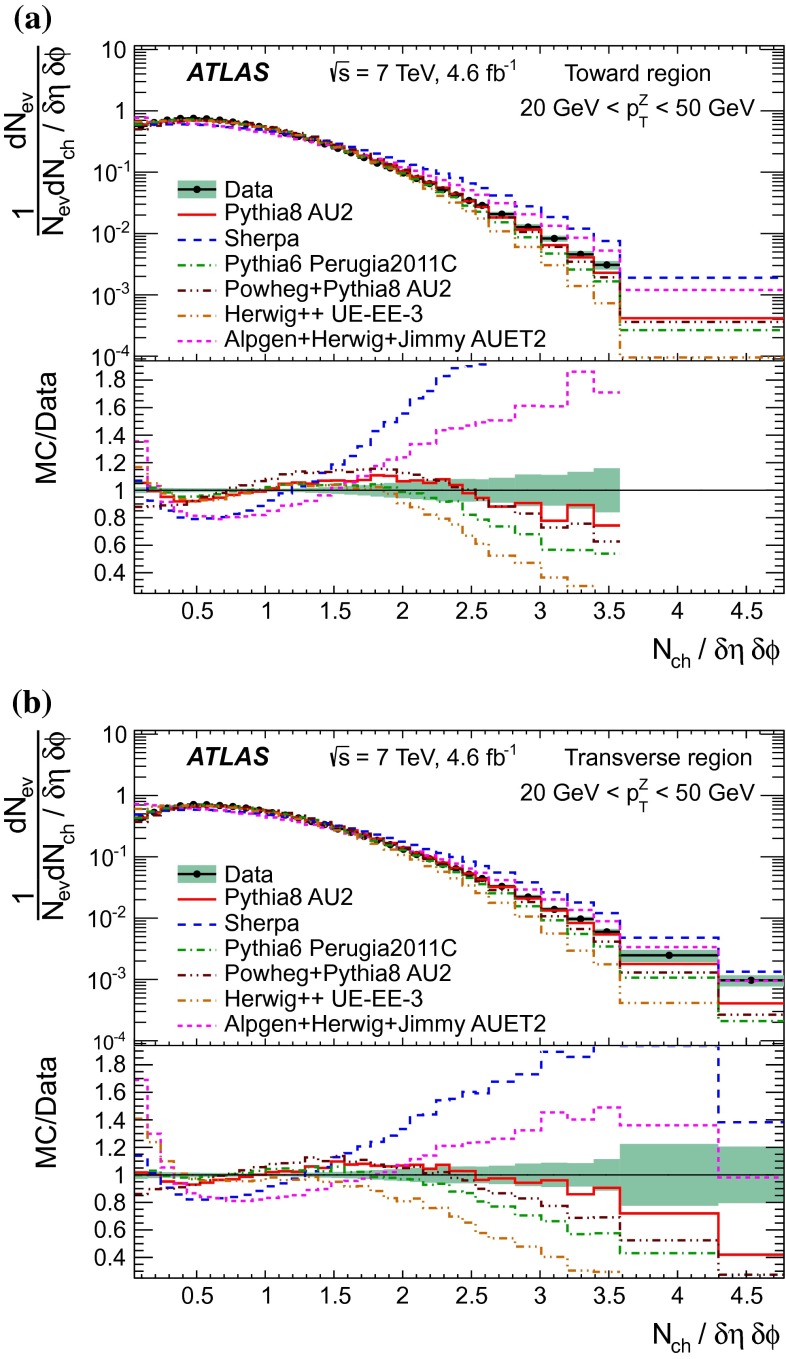
Comparisons of data and MC predictions for charged particle multiplicity density, $N_\text {ch}/\delta \eta \,\delta \phi$ ,  for $Z$-boson transverse momentum, $p_\mathrm {{T}}^\mathrm {{Z}}$, in the interval 20–50 $\text {GeV}$, in the toward (**a**) and transverse (**b**) regions. The *bottom panels* in each plot show the ratio of MC predictions to data. The *shaded bands* represent the combined statistical and systematic uncertainties, while the *error bars* show the statistical uncertainties

In Figs. 7 and 8, for a selected interval of $p_\mathrm {{T}}^\mathrm {{Z}}$, between 20–50 GeV, the $\sum \!p_\mathrm {T}/\delta \eta \,\delta \phi$ distributions in all the UE regions are compared to various MC model predictions (as described in Table 2). For $\sum \!p_\mathrm {T}/\delta \eta \,\delta \phi < 0.1$ GeV, there is a large spread in the predictions of the MC models relative to the data, with Powheg providing the best description. The intermediate region with $0.1 < \sum \!p_\mathrm {T}/\delta \eta \,\delta \phi < 1$ GeV, is well reproduced by most of the MC models. For the higher $\sum \!p_\mathrm {T}/\delta \eta \,\delta \phi$ ranges, most of the MC models underestimate the number of events, with the exception of Sherpa and Alpgen, which have previously been shown to provide good models of multi-jet produced in association with a $Z$-boson  [43]. This observation may indicate that even the trans-min region is not free of additional jets coming from the hard scatter.

**Fig. 12:**
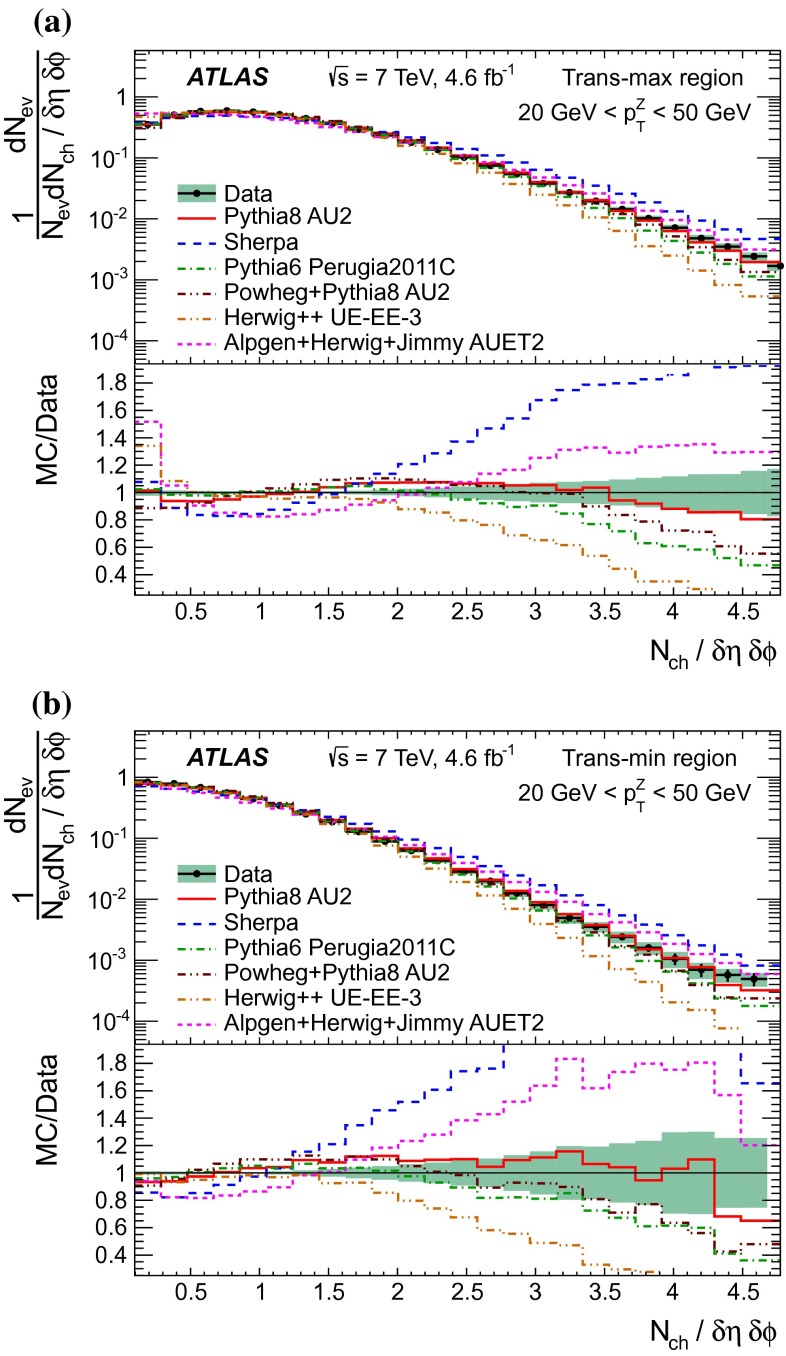
Comparisons of data and MC predictions for charged particle multiplicity density, $N_\text {ch}/\delta \eta \,\delta \phi$ ,  for $Z$-boson transverse momentum, $p_\mathrm {{T}}^\mathrm {{Z}}$, in the interval 20–50 $\text {GeV}$, in the trans-max (**a**) and trans-min (**b**) regions. The *bottom panels* in each plot show the ratio of MC predictions to data. The *shaded bands* represent the combined statistical and systematic uncertainties, while the *error bars* show the statistical uncertainties

**Fig. 13:**
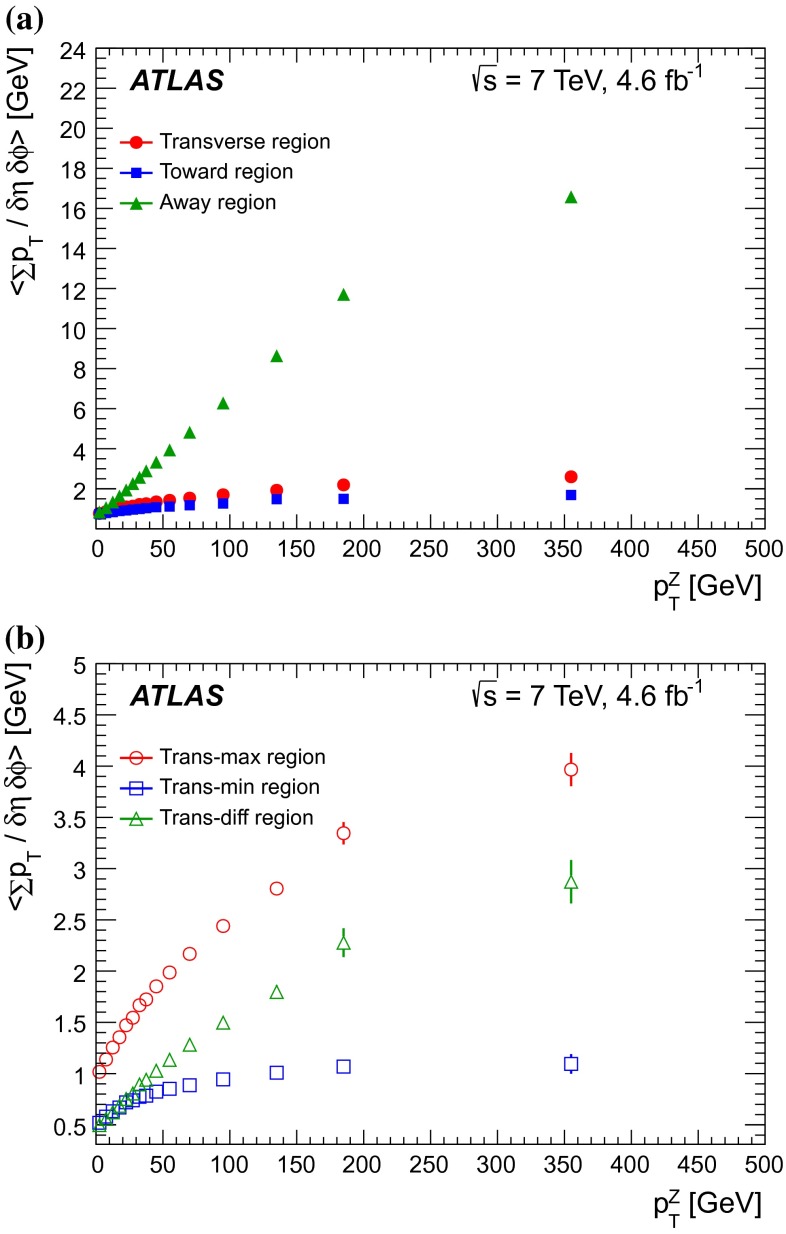
The average values of charged particle scalar $\sum p_\mathrm {T}$ density, $\langle \sum \!p_\mathrm {T}/\delta \eta \,\delta \phi \rangle$, as a function of $Z$-boson transverse momentum, $p_\mathrm {{T}}^\mathrm {{Z}}$, in the transverse, toward and away regions (**a**), and in the trans-max, trans-min and trans-diff regions (**b**). The results are plotted at the center of each $p_\mathrm {{T}}^\mathrm {{Z}}$ bin. The *error bars* depict combined statistical and systematic uncertainties

The distributions of the charged particle multiplicity density in the four UE regions are shown in Figs. 9 and 10 for the same $p_\mathrm {{T}}^\mathrm {{Z}}$ intervals used in Figs. 5 and 6, respectively. The distributions in the transverse, toward and trans-max regions exhibit similar features, with the exception of the largest multiplicities, which are suppressed in the trans-min region, compared to the trans-max one. In the trans-min region, as for the $\sum \!p_\mathrm {T}/\delta \eta \,\delta \phi$ distribution, limited dependence on $p_\mathrm {{T}}^\mathrm {{Z}}$ is observed at low multiplicity. The suppression of large multiplicities in the trans-min region is more pronounced in the lower $p_\mathrm {{T}}^\mathrm {{Z}}$ intervals. The comparison of these multiplicity distributions to various MC models, in the same $p_\mathrm {{T}}^\mathrm {{Z}}$ interval, between 20–50 GeV, is shown in Figs. 11 and 12 for all the UE regions. In contrast to the $\sum \!p_\mathrm {T}/\delta \eta \,\delta \phi$ distributions, none of the MC models, except Pythia 8, describes the data distributions, in particular for $N_\text {ch}/\delta \eta \,\delta \phi > 2$.

### Average distributions

The evolution of the event activity in the four UE regions with the hard scale can be conveniently summarised by the average value of the UE observables as a function of $p_\mathrm {{T}}^\mathrm {{Z}}$.

In Fig. 13 the dependence of $\langle \sum \!p_\mathrm {T}/\delta \eta \,\delta \phi \rangle$ on $p_\mathrm {{T}}^\mathrm {{Z}}$ is compared in different UE regions. The activity levels in the toward and transverse regions are both small compared to the activity in the away region. This difference increases with increasing $p_\mathrm {{T}}^\mathrm {{Z}}$. The away region density is large due to the presence in most cases of a jet balancing the $Z$-boson in $p_\mathrm {T}$. The density in the transverse region is seen to be systematically higher than that in the toward region, which can be explained by the fact that for high $p_\mathrm {{T}}^\mathrm {{Z}}$, additional radiated jets balancing $p_\mathrm {{T}}^\mathrm {{Z}}$ affect the transverse region more than the toward region [43]. The difference between the three regions disappears at low $p_\mathrm {{T}}^\mathrm {{Z}}$ due to the fact that the UE regions are not well defined with respect to the actual $Z$-boson direction.

**Fig. 14:**
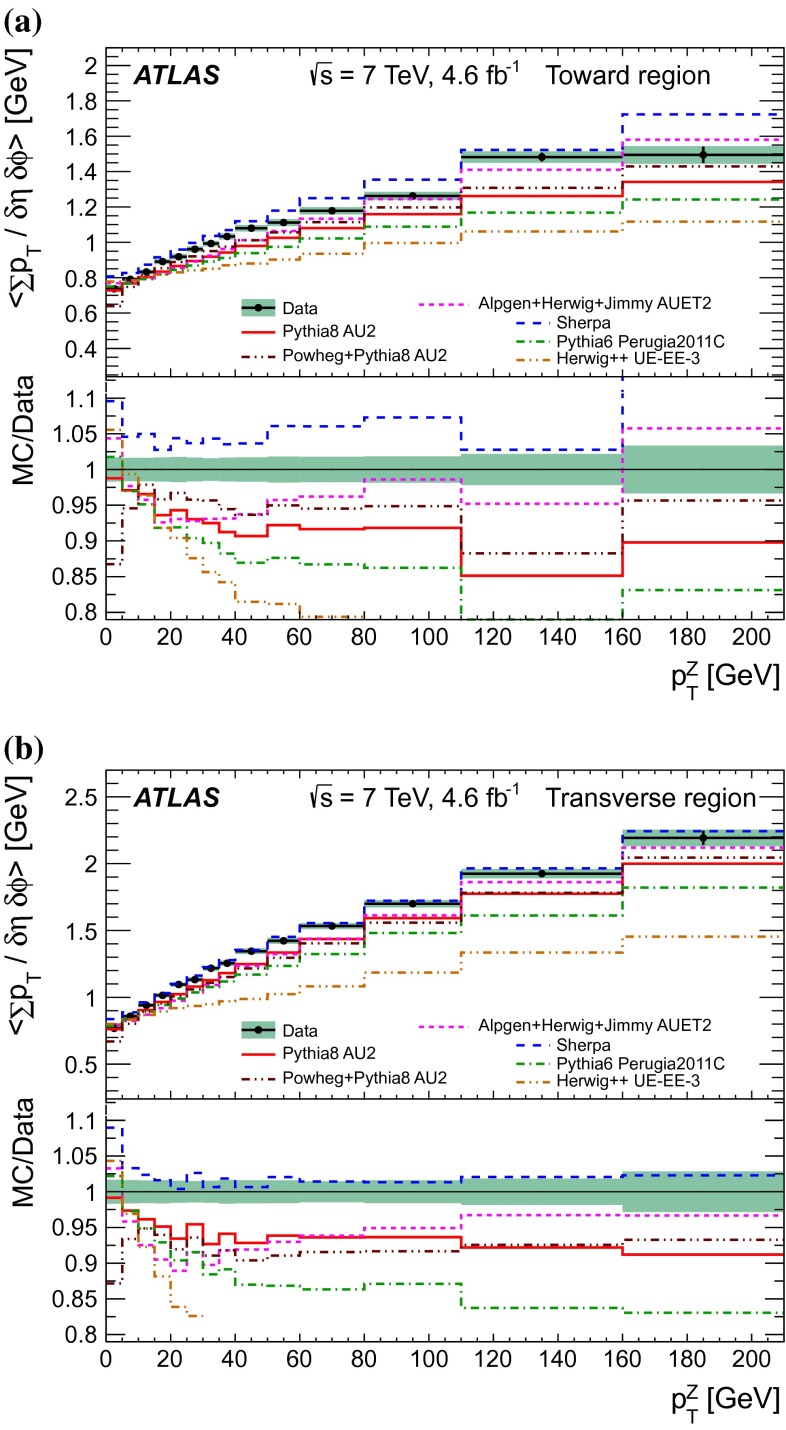
Comparison of data and MC predictions for charged particle scalar $\sum p_\mathrm {T}$ density average values, $\langle \sum \!p_\mathrm {T}/\delta \eta \,\delta \phi \rangle$, as a function of $Z$-boson transverse momentum, $p_\mathrm {{T}}^\mathrm {{Z}}$, in the toward (**a**) and transverse (**b**) regions. The *bottom panels* in each plot show the ratio of MC predictions to data. The *shaded bands* represent the combined statistical and systematic uncertainties, while the *error bars* show the statistical uncertainties

**Fig. 15:**
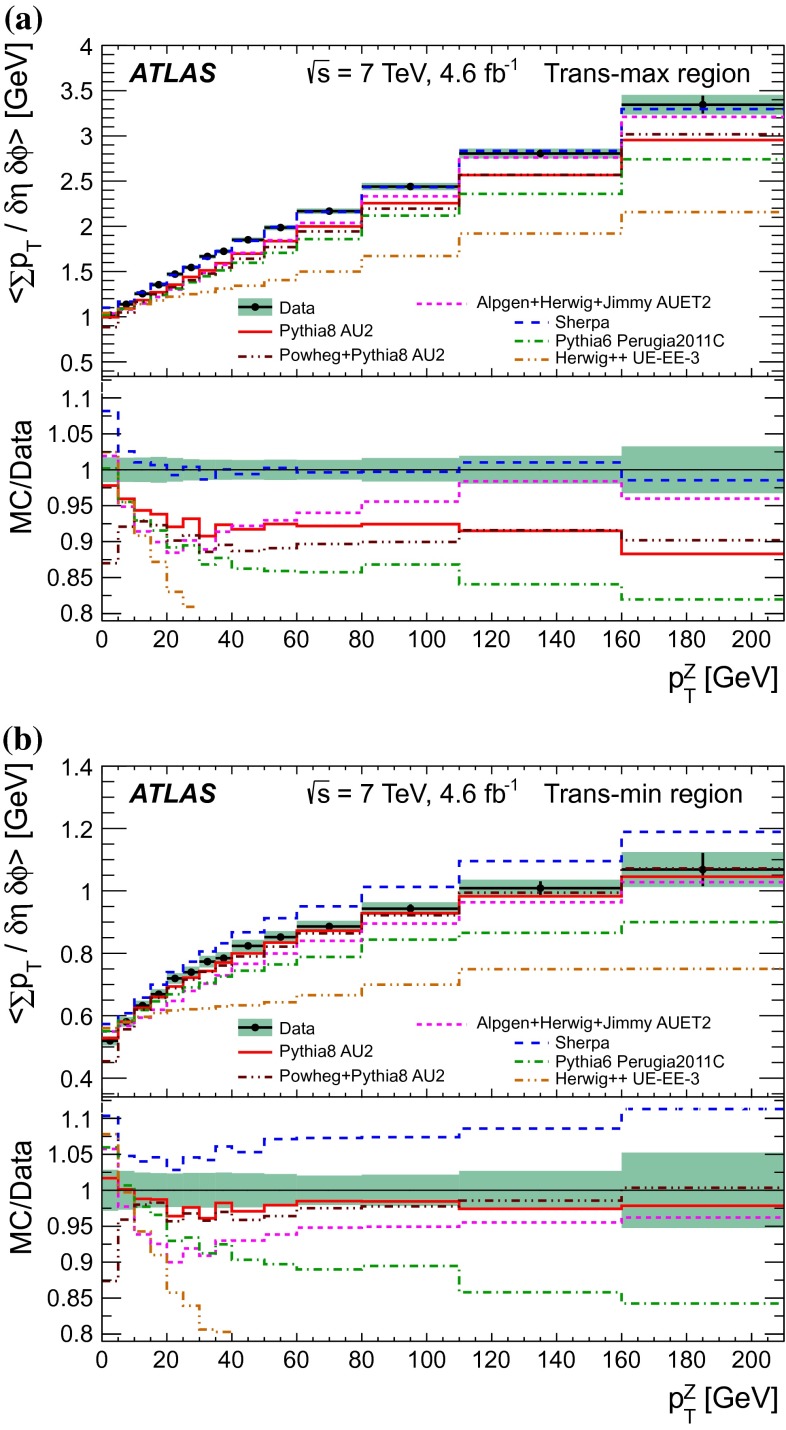
Comparison of data and MC predictions for charged particle scalar $\sum p_\mathrm {T}$ density average values, $\langle \sum \!p_\mathrm {T}/\delta \eta \,\delta \phi \rangle$, as a function of $Z$-boson transverse momentum, $p_\mathrm {{T}}^\mathrm {{Z}}$, in the trans-max (**a**) and trans-min (**b**) regions. The *shaded bands* represent the combined statistical and systematic uncertainties, while the *error bars* show the statistical uncertainties

In Fig. 13, $\langle \sum \!p_\mathrm {T}/\delta \eta \,\delta \phi \rangle$ is seen to rise much faster as a function of $p_\mathrm {{T}}^\mathrm {{Z}}$ in the trans-max region than in the trans-min region. The slowing down of the rise of $\langle \sum \!p_\mathrm {T}/\delta \eta \,\delta \phi \rangle$ at high $p_\mathrm {{T}}^\mathrm {{Z}}$ in the most UE-sensitive toward and trans-min regions is consistent with an assumption [46] of a full overlap between the two interacting protons in impact parameter space at high hard scales.

The comparison of the $\langle \sum \!p_\mathrm {T}/\delta \eta \,\delta \phi \rangle$ distribution as a function of $p_\mathrm {{T}}^\mathrm {{Z}}$ with the predictions of various MC models is shown in Figs. 14 and 15 in the UE regions sensitive to the underlying event characteristics. For clarity of comparison, the statistically least significant $p_\mathrm {{T}}^\mathrm {{Z}} > 210$ GeV bin is omitted. The variation in the range of predictions is quite wide, although less so than for the differential $\sum p_\mathrm {T}$ distributions. The best description of the transverse and trans-max regions is given by Sherpa, followed by Pythia 8, Alpgen and Powheg. The observation that the multi-leg and NLO generator predictions are closer to the data than most of the pure parton shower generators suggests that these regions are affected by the additional jets coming from the hard interaction. Jet multiplicities in events with a $Z$-boson have been studied by the LHC experiments [43], and they are well described by Sherpa and Alpgen.

The discrepancy between the Pythia 8 AU2 tune and the Pythia 6 Perugia tune possibly indicates the effect of using LHC UE data for the former in addition to the shower model improvement. In the trans-min region, which is the most sensitive to the UE, none of the models fully describe the data. Apart from Herwig++, and Sherpa, which predicts a faster rise of $\sum p_\mathrm {T}$ than observed in data, the other generators model the data better in the trans-min region than they do in the transverse or trans-max regions. This possibly indicates that in the LO shower generators the underlying event is well modelled but perturbative jet activity is not.

**Fig. 16:**
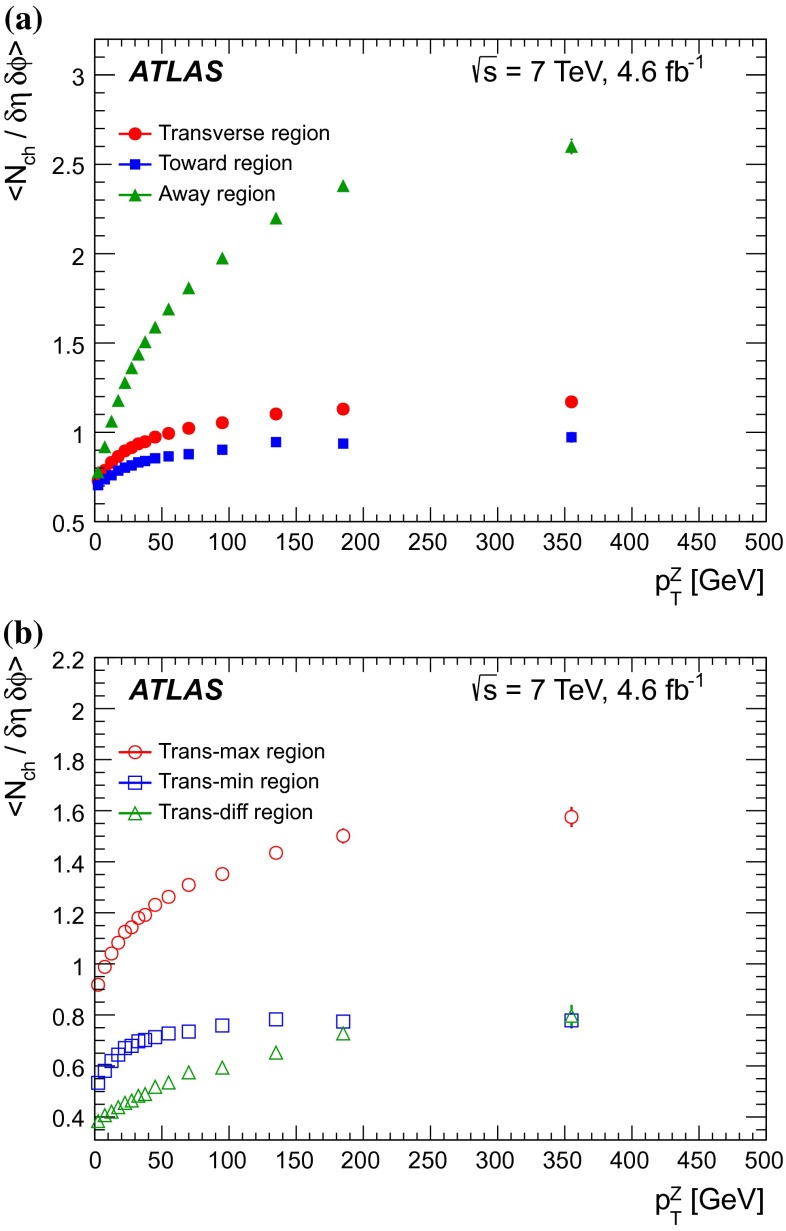
The average values of charged particle multiplicity density, $\langle N_\text {ch}/\delta \eta \,\delta \phi \rangle$ , as a function of $Z$-boson transverse momentum, $p_\mathrm {{T}}^\mathrm {{Z}}$, in the transverse, toward and away regions (**a**), and in the trans-max, trans-min and trans-diff regions (**b**). The results are plotted at the center of each $p_\mathrm {{T}}^\mathrm {{Z}}$ bin. The *error bars* depict combined statistical and systematic uncertainties

In Fig. 16, $\langle N_\text {ch}/\delta \eta \,\delta \phi \rangle$ is shown as a function of $p_\mathrm {{T}}^\mathrm {{Z}}$ in the different UE regions. The profiles behave in a similar way to $\langle \sum \!p_\mathrm {T}/\delta \eta \,\delta \phi \rangle$. However, the trans-diff $\langle N_\text {ch}/\delta \eta \,\delta \phi \rangle$ activity is lower than that for trans-min, while for $\langle \sum \!p_\mathrm {T}/\delta \eta \,\delta \phi \rangle$, it is the other way around. This indicates that the trans-diff region, which is a measure of extra activity in the trans-max region over the trans-min region, is populated by a few particles with high transverse momentum, as expected for the leading constituents of jets.

**Fig. 17:**
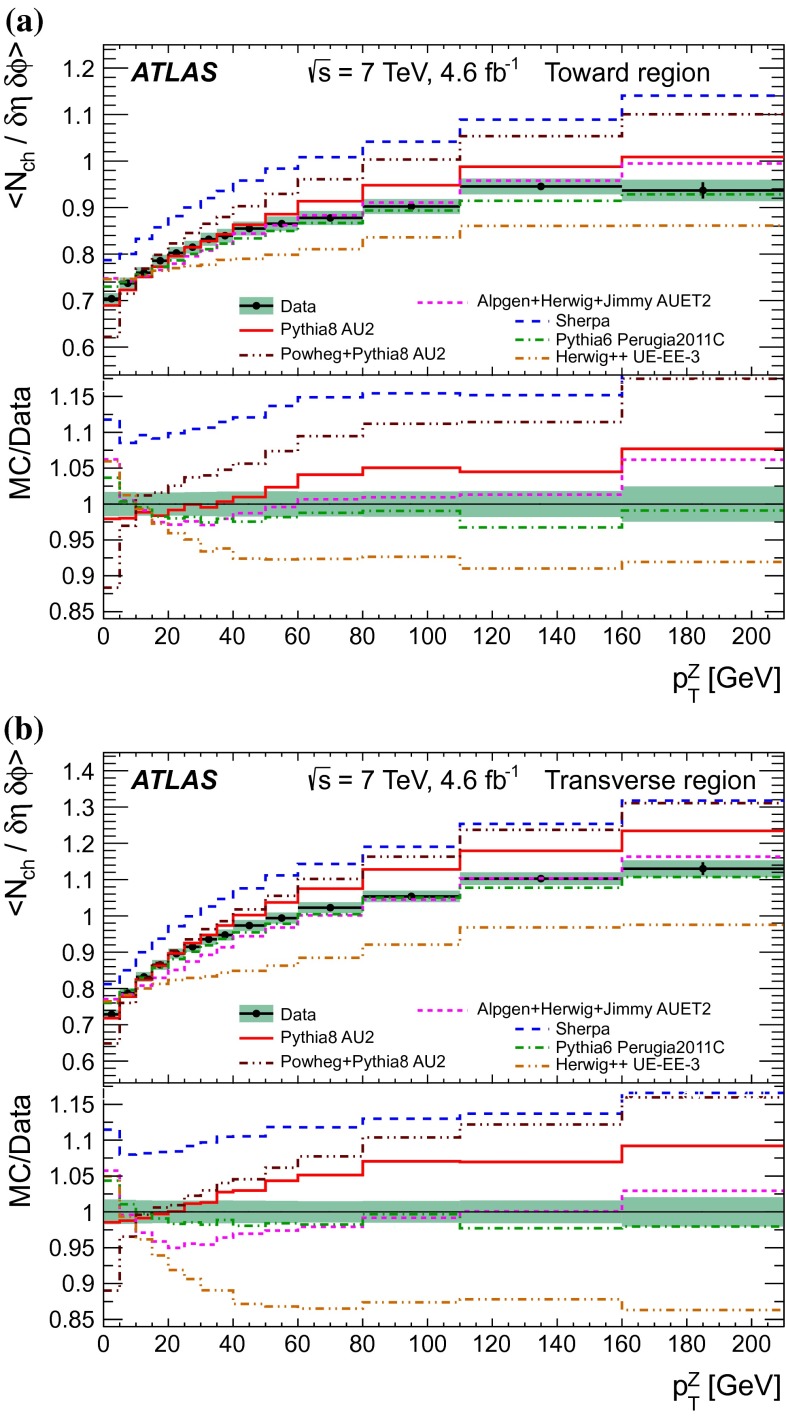
Comparison of data and MC predictions for charged particle multiplicity density average values, $\langle N_\text {ch}/\delta \eta \,\delta \phi \rangle$ , as a function of $Z$-boson transverse momentum, $p_\mathrm {{T}}^\mathrm {{Z}}$, in the toward (**a**) and transverse (**b**) regions. The *bottom panels* in each plot show the ratio of MC predictions to data. The *shaded bands* represent the combined statistical and systematic uncertainties, while the *error bars* show the statistical uncertainties

**Fig. 18:**
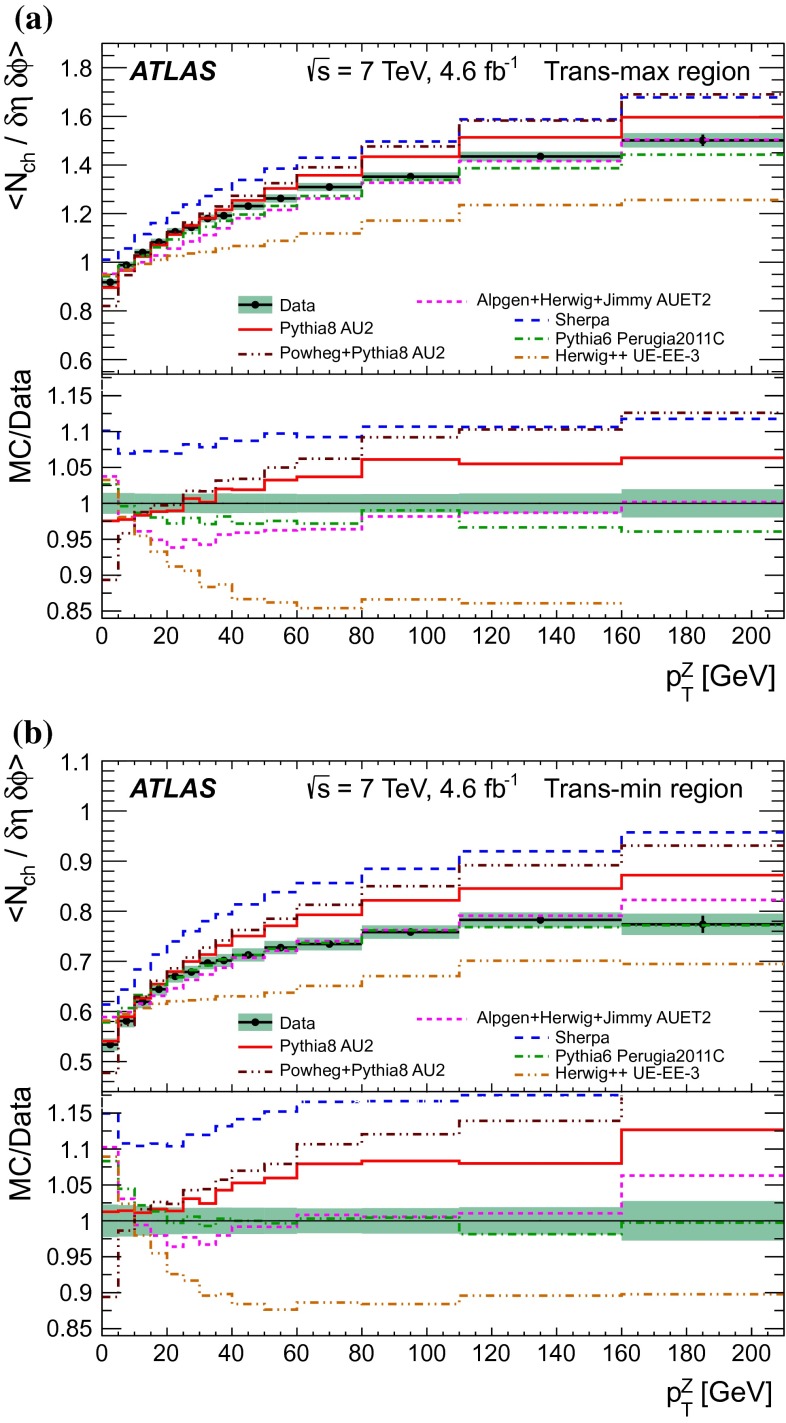
Comparison of data and MC predictions for charged particle multiplicity density average values, $\langle N_\text {ch}/\delta \eta \,\delta \phi \rangle$ , as a function of $Z$-boson transverse momentum, $p_\mathrm {{T}}^\mathrm {{Z}}$, in the trans-max (**a**) and trans-min (**b**) regions. The *bottom panels* in each plot show the ratio of MC predictions to data. The *shaded bands* represent the combined statistical and systematic uncertainties, while the *error bars* show the statistical uncertainties

In Figs. 17 and 18, in which various MC model predictions are compared to $\langle N_\text {ch}/\delta \eta \,\delta \phi \rangle$ as a function of $p_\mathrm {{T}}^\mathrm {{Z}}$, a different pattern from that of $\langle \sum \!p_\mathrm {T}/\delta \eta \,\delta \phi \rangle$ is observed. The Pythia 6 Perugia 2011C tune and Alpgen provide the closest predictions in all three regions. Sherpa, Pythia 8 and Powheg predict higher average multiplicities, with Sherpa being the farthest from the data. On the other hand, Herwig++ mostly underestimates the data.

**Fig. 19:**
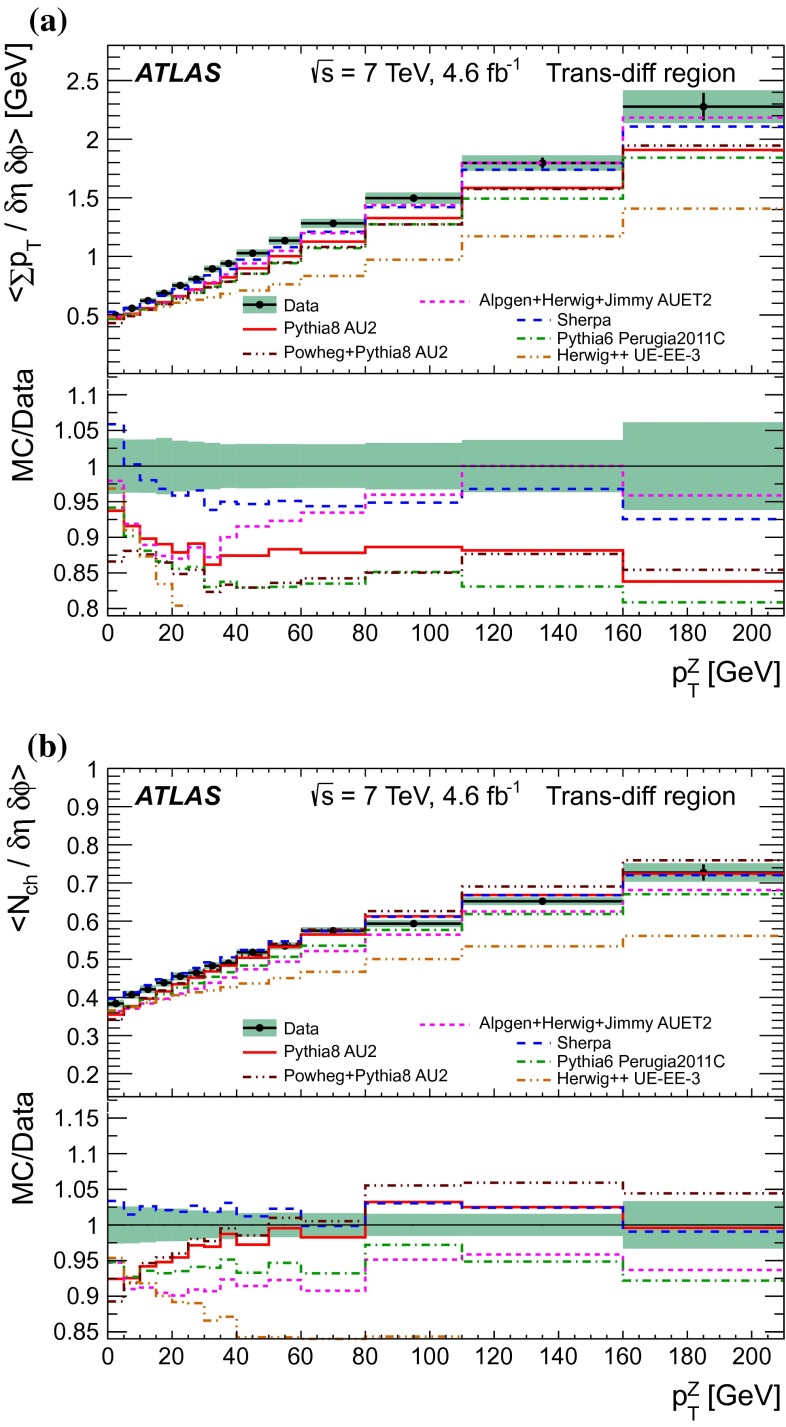
Comparison of data and MC predictions for charged particle scalar $\sum p_\mathrm {T}$ density average values, $\langle \sum \!p_\mathrm {T}/\delta \eta \,\delta \phi \rangle$ (**a**), and multiplicity average values, $\langle N_\text {ch}/\delta \eta \,\delta \phi \rangle$ (**b**) as a function of $Z$-boson transverse momentum, $p_\mathrm {{T}}^\mathrm {{Z}}$, in the trans-diff region. The *shaded bands* represent the combined statistical and systematic uncertainties, while the *error bars* show the statistical uncertainties

The $\langle \sum \!p_\mathrm {T}/\delta \eta \,\delta \phi \rangle$ and $\langle N_\text {ch}/\delta \eta \,\delta \phi \rangle$ distributions as functions of $p_\mathrm {{T}}^\mathrm {{Z}}$ in the trans-diff region are compared with the MC model predictions in Fig. 19. While all MC models, except for Herwig++ predict the multiplicity fairly well, only Sherpa and Alpgen predict the $\sum p_\mathrm {T}$ average values well in certain ranges. The better modelling of this region by MC models with additional jets coming from matrix element rather than from parton shower again confirms that the trans-diff region is most sensitive to the additional radiated jets.

**Fig. 20:**
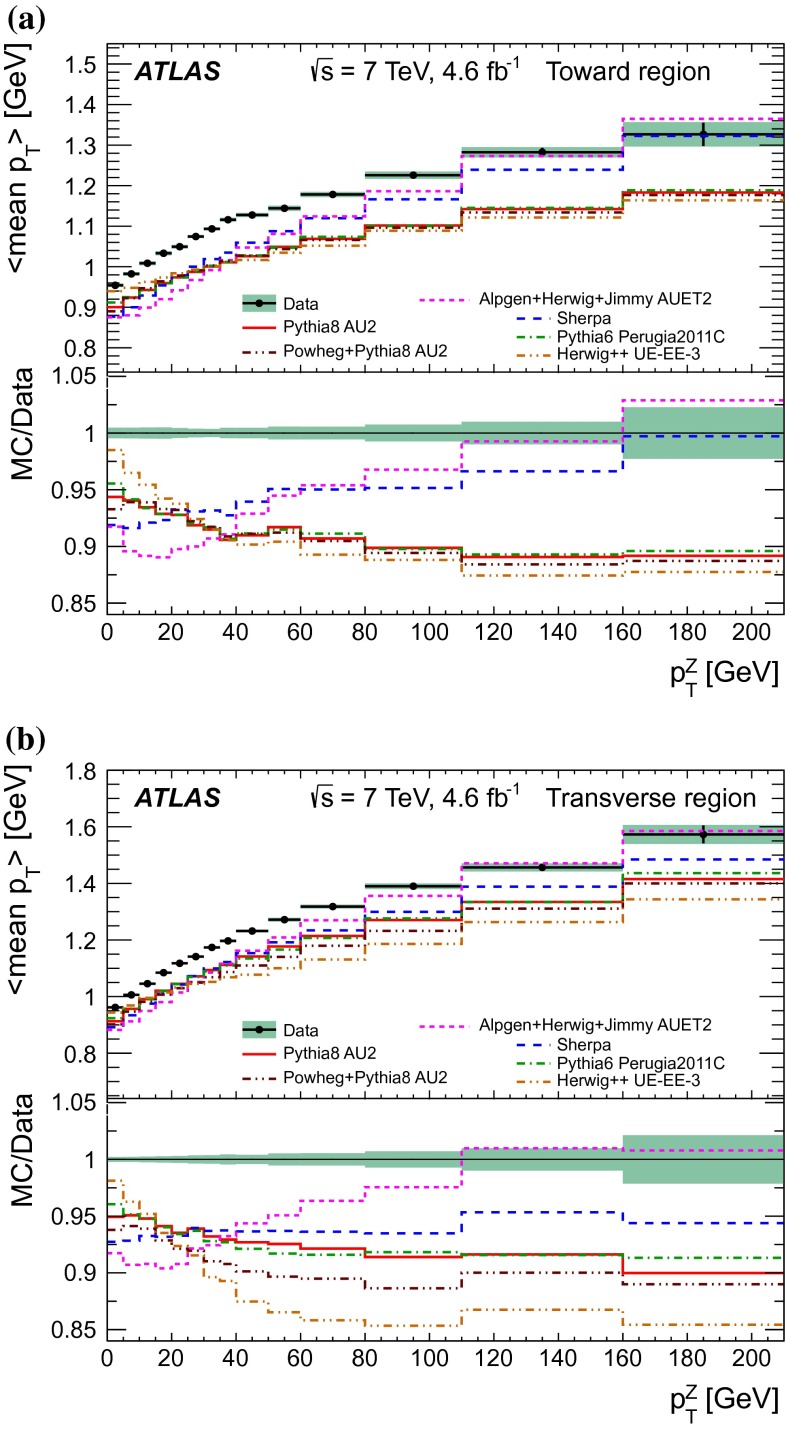
Comparison of data and MC predictions for charged particle mean $p_\mathrm {T}$ as a function of $Z$-boson transverse momentum, $p_\mathrm {{T}}^\mathrm {{Z}}$, in the toward (**a**) and transverse (**b**) regions. The *bottom panels* in each plot show the ratio of MC predictions to data. The *shaded bands* represent the combined statistical and systematic uncertainties, while the *error bars* show the statistical uncertainties

**Fig. 21:**
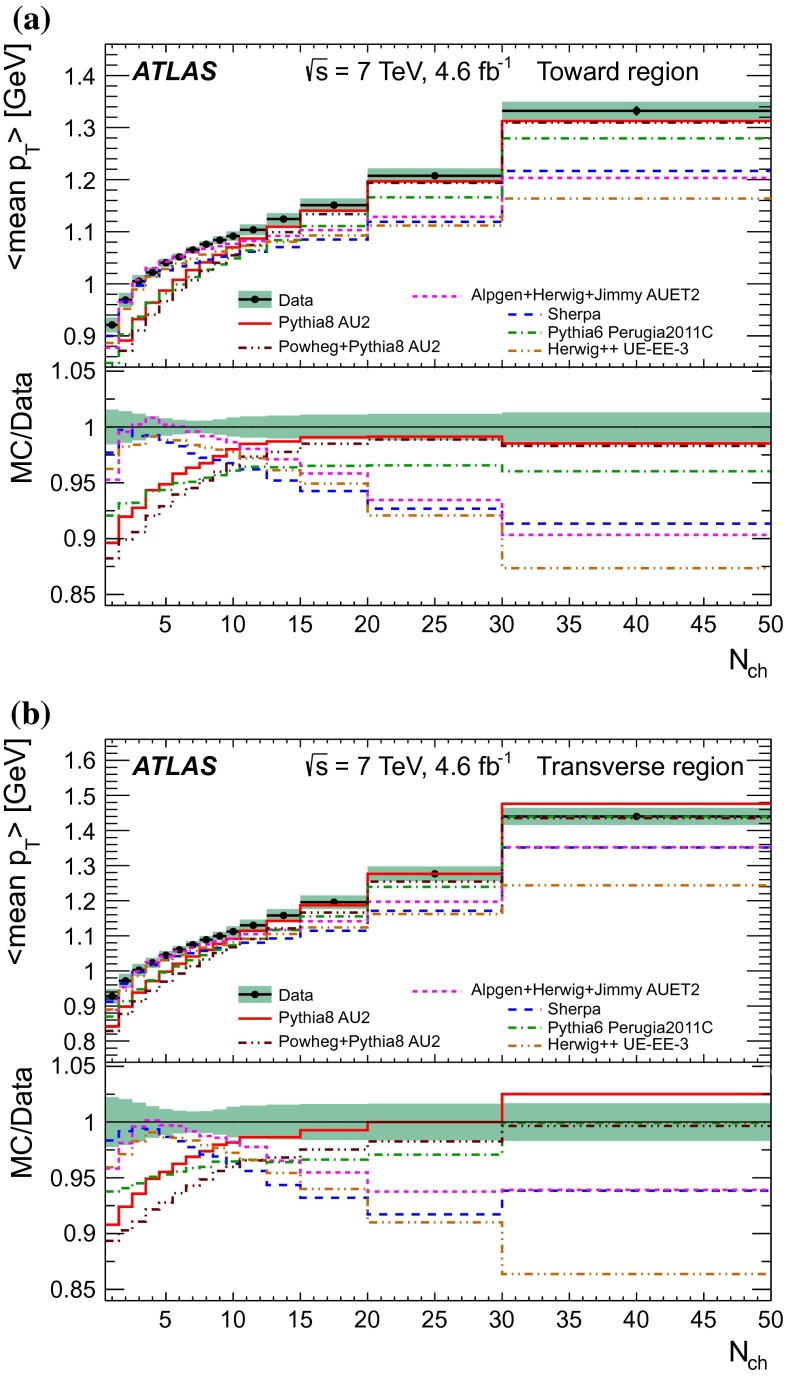
Comparison of data and MC predictions for charged particle mean $p_\mathrm {T}$ as a function of charged particle multiplicity, $N_\text {ch}$, in the toward (**a**) and transverse (**b**) regions. The *bottom panel* in each plot shows the ratio of MC predictions to data. The *shaded bands* represent the combined statistical and systematic uncertainties, while the *error bars* show the statistical uncertainties

The difficulty of describing the $\langle \sum \!p_\mathrm {T}/\delta \eta \,\delta \phi \rangle$ and $\langle N_\text {ch}/\delta \eta \,\delta \phi \rangle$ average values simultaneously in MC models is reflected in the comparison of data and MC model predictions for $\langle p_\mathrm {T} \rangle$ in Fig. 20. The $\langle p_\mathrm {T} \rangle$ as a function of $p_\mathrm {{T}}^\mathrm {{Z}}$ is reasonably described by Alpgen and Sherpa for high $p_\mathrm {{T}}^\mathrm {{Z}}$, while all the other models predict softer spectra. The correlation of $\langle p_\mathrm {T} \rangle$ with $N_\text {ch}$, shown in Fig. 21, follows the pattern established by previous experiments, with a slow increase in mean $p_\mathrm {T}$ with increasing $N_\text {ch}$. This observable is sensitive to the colour reconnection model in the MC generators. No MC model is able to predict the full shape in either region. Overall the Pythia 8 prediction is the closest to the data, followed by Pythia 6 and Powheg, although for $N_\text {ch} < 5$, all three have much softer distributions than the data. The other models do well in this low $N_\text {ch}$ region, but are then much lower than the data for high $N_\text {ch}$.

From all the distributions considered, it can be inferred that the jets radiated from the hard scatter will affect the underlying event observables and therefore these must be properly reproduced in order to obtain an accurate MC description of the UE. The UE region least affected by the presence of extra jets is the trans-min region.

### Comparison with other ATLAS measurements

The results from this analysis are compared to the results obtained when the leading object is either a charged particle [1] or a hadronic jet [5]. The underlying event analysis with a leading charged particle was performed with the early 2010 data, while the analysis using events with jets utilises the full 2010 dataset.

**Fig. 22:**
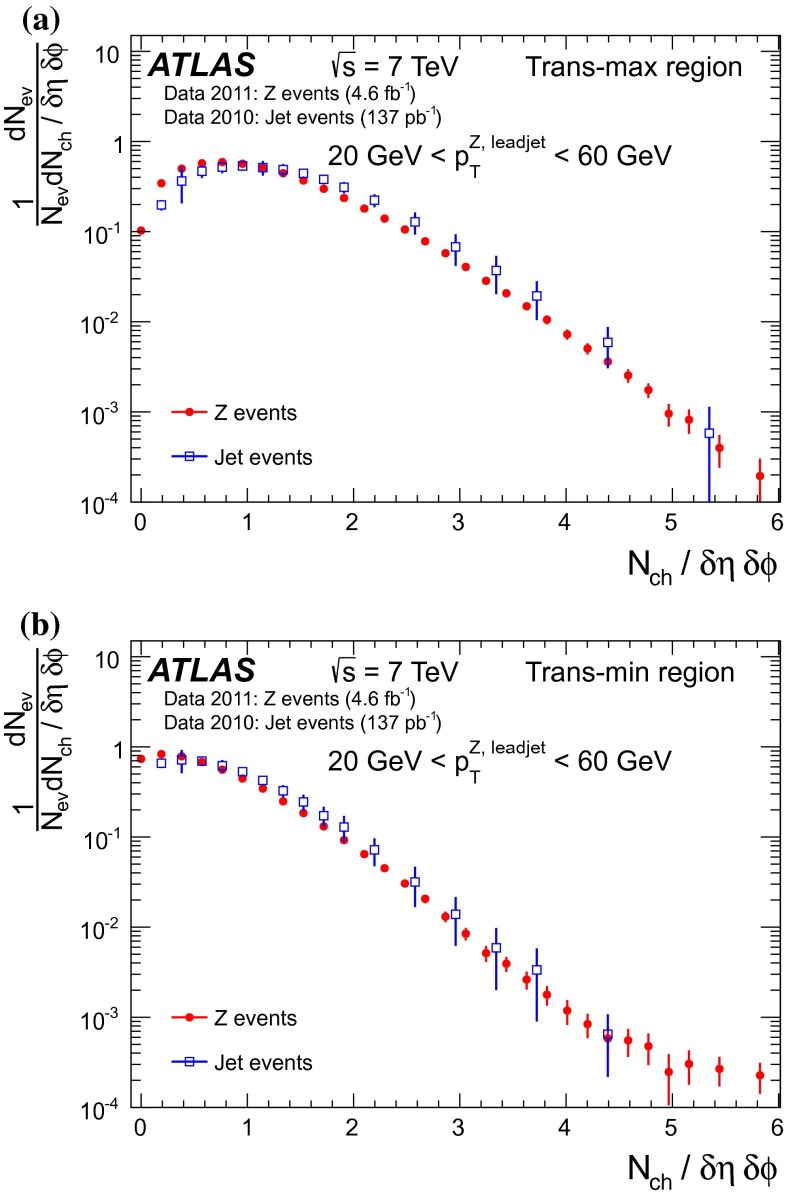
Distributions of charged particle multiplicity density, $N_\text {ch}/\delta \eta \,\delta \phi$ , compared between jet and $Z$-boson events, respectively in $Z$-boson transverse momentum, $p_\mathrm {{T}}^\mathrm {{Z}}$ and leading jet transverse momentum, $p_\mathrm{T }^\mathrm{leadjet }$ interval between 20–60 GeV, in the trans-max (**a**) and trans-min (**b**) regions. The *error bars* in each case show the combined statistical and systematic uncertainties

**Fig. 23:**
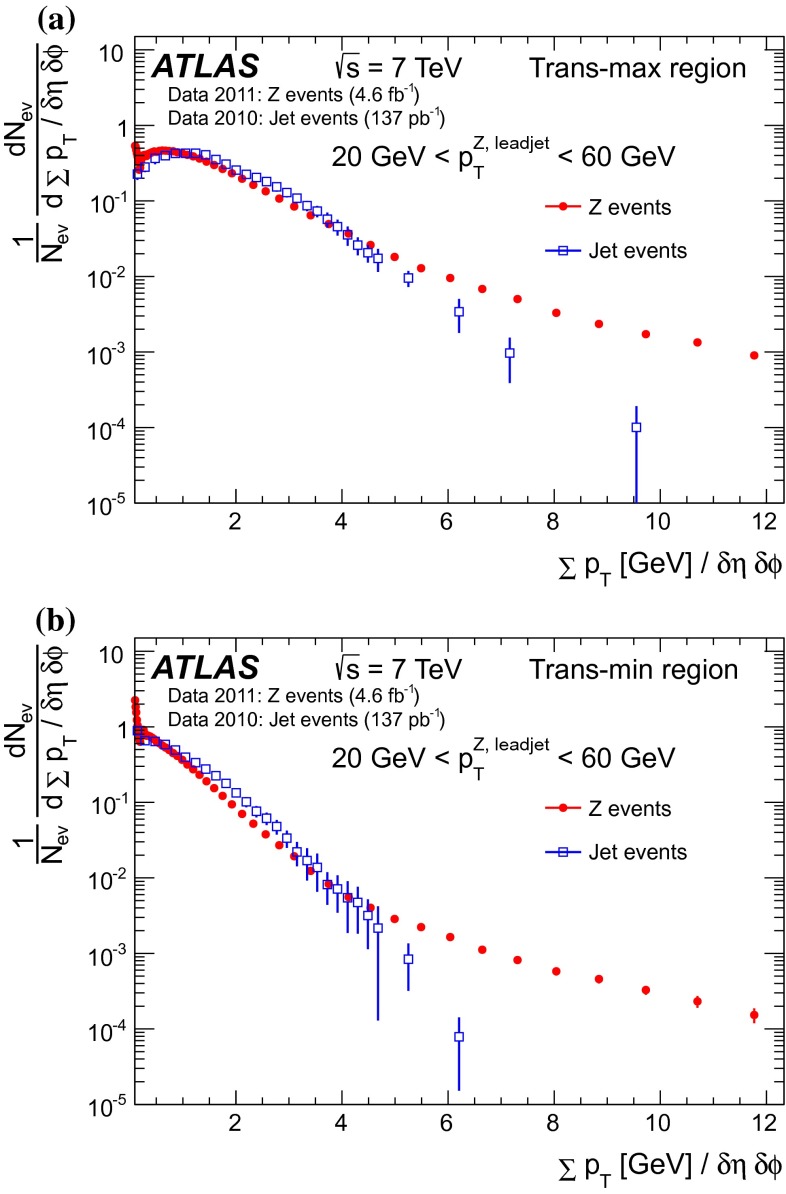
Distributions of charged particle scalar $p_\mathrm {T}$ sum density, $\sum \!p_\mathrm {T}/\delta \eta \,\delta \phi$, compared between jet and $Z$-boson events, respectively in $Z$-boson transverse momentum, $p_\mathrm {{T}}^\mathrm {{Z}}$ and leading jet transverse momentum, $p_\mathrm{T }^\mathrm{leadjet }$ interval between 20–60 GeV, in the trans-max (**a**) and trans-min (**b**) regions. The *error bars* in each case show the combined statistical and systematic uncertainties

The differential $N_\text {ch}/\delta \eta \,\delta \phi$ and $\sum \!p_\mathrm {T}/\delta \eta \,\delta \phi$ distributions for leading jet and $Z$-boson events are compared in Figs. 22 and 23 for the trans-max and trans-min regions. While the $N_\text {ch}/\delta \eta \,\delta \phi$ distributions are similar, a clear difference is observed in the high tails of the $\sum \!p_\mathrm {T}/\delta \eta \,\delta \phi$ distribution, which are more populated in $Z$-boson events than in jet events. This difference was traced to the definition of the leading object. In the case of jets, the accompanying activity can never contain jets with a $p_\mathrm {T}$ higher than that of the leading jet, whereas there is no such restriction for $Z$-boson events. As a test, the average $\sum p_\mathrm {T}$ was determined for $Z$-boson events after rejecting all events in which at the detector level there was a jet with $p_\mathrm {T}$ higher than the $p_\mathrm {{T}}^\mathrm {{Z}}$, with jets selected as in [5]. The average was found to be about 20–30 % lower than for the standard selection, and the average values in jet and $Z$-boson events are in close agreement in this case.

**Fig. 24:**
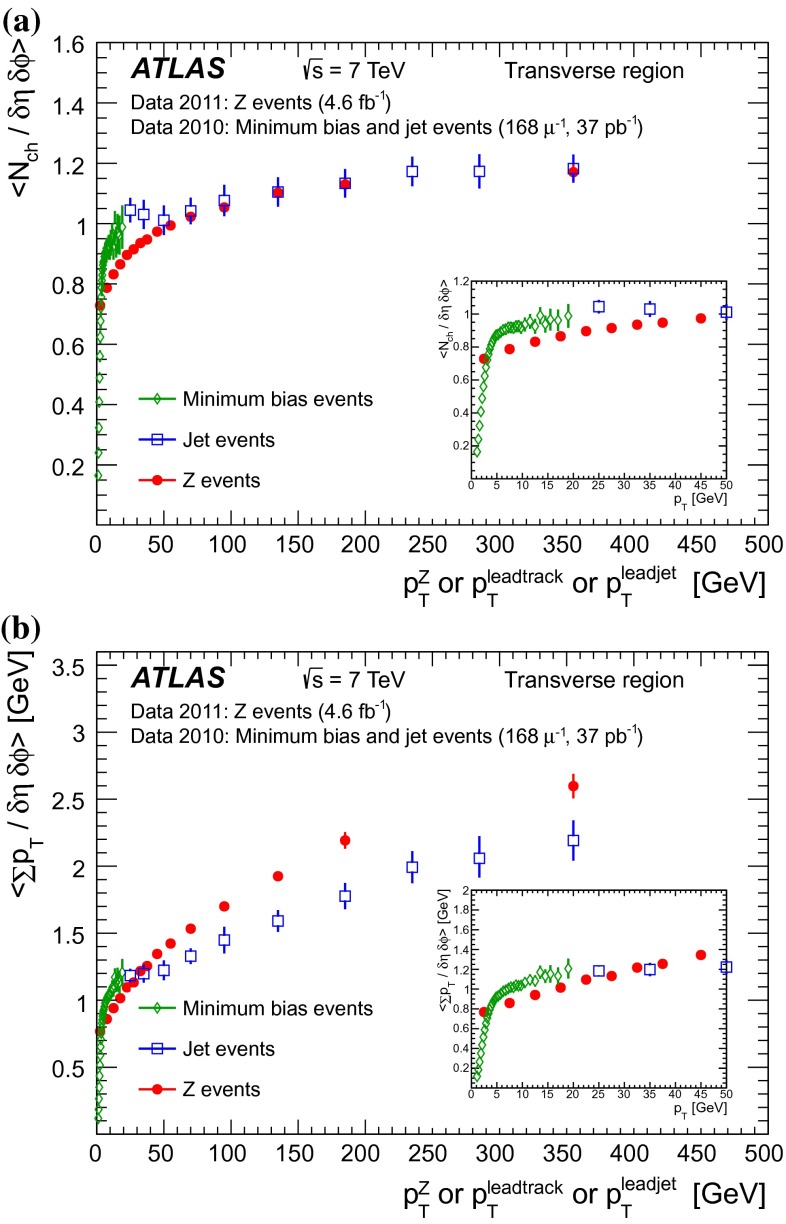
Charged particle multiplicity average values, $\langle N_\text {ch}/\delta \eta \,\delta \phi \rangle$ (**a**), and scalar $\sum p_\mathrm {T}$ density average values, $\langle \sum \!p_\mathrm {T}/\delta \eta \,\delta \phi \rangle$ (**b**), compared between leading charged particle (*minimum bias*), leading jet and $Z$-boson events, respectively as functions of leading track transverse momentum, $p_\mathrm{T }^\mathrm{lead }$, leading jet transverse momentum, $p_\mathrm{T }^\mathrm{leadjet }$ and $Z$-boson transverse momentum, $p_\mathrm {{T}}^\mathrm {{Z}}$, in the transverse region. The *error bars* in each case show the combined statistical and systematic uncertainties. The *insets* show the region of transition between the leading charged particle and leading jet results in more detail

**Fig. 25:**
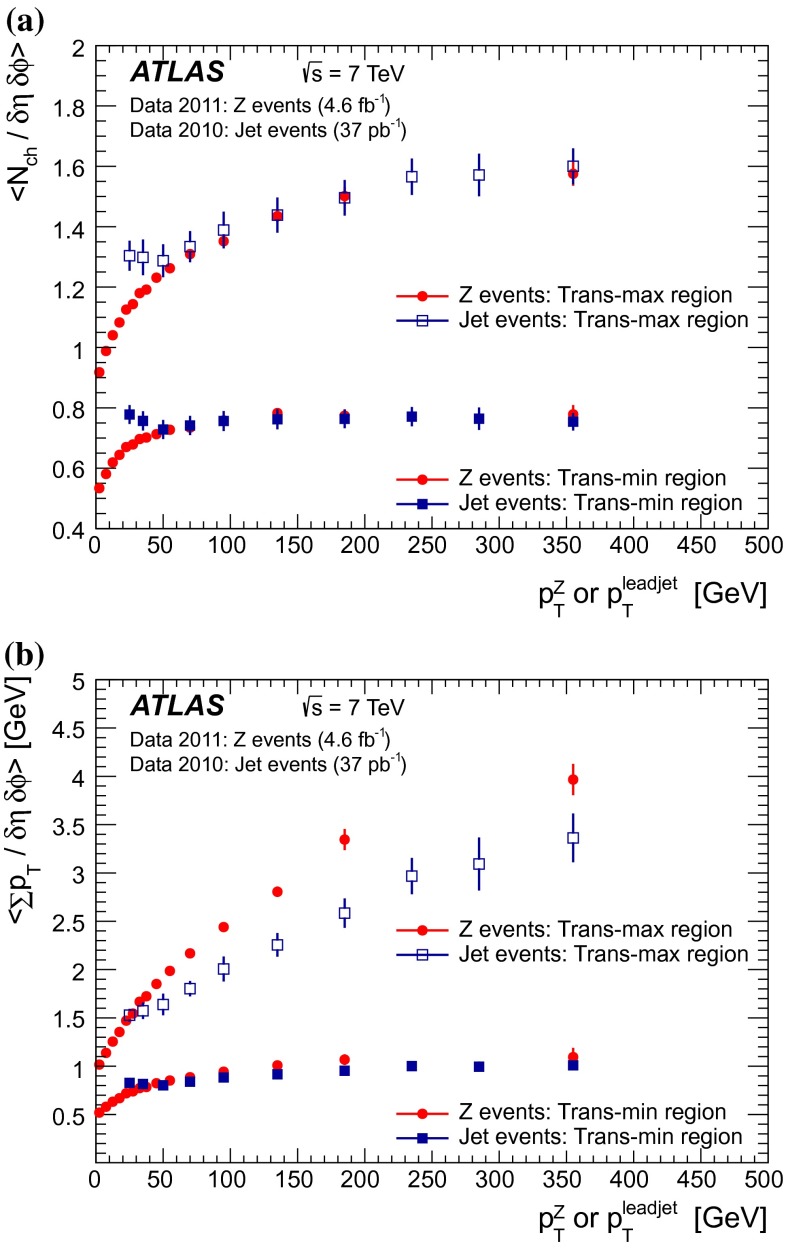
Charged particle multiplicity average values, $\langle N_\text {ch}/\delta \eta \,\delta \phi \rangle$ (**a**), and scalar $\sum p_\mathrm {T}$ density average values, $\langle \sum \!p_\mathrm {T}/\delta \eta \,\delta \phi \rangle$ (**b**), compared between leading jet and $Z$-boson events, respectively as functions of leading jet transverse momentum, $p_\mathrm{T }^\mathrm{leadjet }$ and $Z$-boson transverse momentum, $p_\mathrm {{T}}^\mathrm {{Z}}$, in the transverse, trans-max and trans-min-regions. The *error bars* in each case show the combined statistical and systematic uncertainties

The hard scales used for the analyses are different and the choice of the main observable used to assess the evolution of the underlying event reflects this to a certain extent in the figures. Nevertheless, certain common qualitative features can be observed by comparing $\langle \sum \!p_\mathrm {T}/\delta \eta \,\delta \phi \rangle$ and $\langle N_\text {ch}/\delta \eta \,\delta \phi \rangle$ as functions of the leading object $p_\mathrm {T}$ in the transverse region, and also separated into the trans-max/min regions as shown in Figs. 24 and 25. The measurements with a leading jet are complementary to the measurements with a leading track, and a smooth continuation at $20$ GeV is observed (in Fig. 24), corresponding to the lowest jet $p_\mathrm {T}$ for which the jet measurement could be performed and the highest leading track momentum included in the leading track analysis. Where the $p_\mathrm {T}$ of the leading object is less than $50$ GeV, a large difference is observed both for the $N_\text {ch}$ and $\sum p_\mathrm {T}$ average values between the jet and $Z$-boson measurements in Fig. 24; the increase of the associated activity as a function of the hard scale $p_\mathrm {T}$ is very different in track/jets events from the $Z$-boson events.

Although the $N_\text {ch}$ density is similar in the underlying event associated with a jet to that with a $Z$-boson for higher values of the hard scale (${\ge }50$ GeV), there are residual differences in the average $\sum p_\mathrm {T}$ densities. The activity in events with a $Z$-boson is systematically higher than that in events with jets. From the behaviour of the underlying event properties in the trans-max/min regions in Fig. 25, this difference originates mostly from the trans-max region, due to selection bias discussed previously in this section. The trans-min region is very similar between the two measurements, despite the different hard scales, indicating again that this region is least sensitive to the hard interaction and most sensitive to the MPI component.

## Conclusion

Measurements sensitive to the underlying event have been presented, using an inclusive sample of $Z$-boson decays, obtained from a data set collected in proton–proton collisions at the LHC corresponding to an integrated luminosity of $4.6\;\text {fb}^{-1}$. The transverse and toward regions with respect to the reconstructed $Z$-boson are most sensitive to the underlying event. The transverse region was further subdivided into trans-max and trans-min regions on an event-by-event basis depending on which one had a higher $\sum p_\mathrm {T}$; this subdivision provides additional power to discriminate between the different processes contributing to the underlying event models.

The results show the presence of a hard component in the $p_\mathrm {T}$ distribution of particles, presumably originating from extra jet activity associated with the $Z$-boson production. It is observed in all the investigated regions, with the trans-min region least affected by it. The average underlying event activity increases with $p_\mathrm {{T}}^\mathrm {{Z}}$, until it reaches a plateau, which is again most prominent in the trans-min region. The results have been compared to a number of MC models, using several tunes of commonly used underlying event models. MC model predictions qualitatively describe the data well, but with some significant discrepancies, providing precise information sensitive to the choices of parameters used in the various underlying-event models. Careful tuning of these parameters in the future may improve the description of the data by the different models in future LHC measurements and studies.

The study of such variables in $Z$-boson events provides a probe of the underlying event which is complementary to that from purely hadronic events. A comparison between them shows similar underlying event activity for the trans-min region.

## Footnotes

1 The ATLAS reference system is a Cartesian right-handed coordinate system, with the nominal collision point at the origin. The anti-clockwise beam direction defines the positive $z$-axis, while the positive $x$-axis is defined as pointing from the collision point to the center of the LHC ring and the positive $y$-axis points upwards. The azimuthal angle $\phi$ is measured around the beam axis, and the polar angle $\theta$ is measured with respect to the $z$-axis. The pseudorapidity is given by $\eta = -\ln \tan ( \theta /2 )$. Transverse momentum is defined relative to the beam axis.
